# Predicting schistosome transmission in rural Uganda using water contact data from wearable GPS devices

**DOI:** 10.1038/s44360-026-00118-w

**Published:** 2026-05-22

**Authors:** Fabian Reitzug, Melissa A. Iacovidou, Narcis B. Kabatereine, Betty Nabatte, Max T. Eyre, Benjamin Tinkitina, Anatol M. Byaruhanga, Renaud Lambiotte, Goylette F. Chami

**Affiliations:** 1 Big Data Institute, Nuffield Department of Population Health, University of Oxford, Oxford, United Kingdom; 2 Division of Vector Borne and Neglected Tropical Diseases, Ministry of Health, Kampala, Uganda; 3 London School of Hygiene and Tropical Medicine, London, United Kingdom; 4 Mathematical Institute, University of Oxford, Oxford, United Kingdom

**Keywords:** Risk factors, Parasitic infection, Environmental studies

## Abstract

Granular spatial data and models of human behaviour are currently lacking for schistosomiasis. We collected ten days of wearable GPS logger data from 452 individuals in rural Uganda to model water contact as a proxy indicator of usage at 69 georeferenced open-water sites and 32 public taps or boreholes. Among participants, 63.9 and 33.2% visited at least one water site and tap or borehole, respectively. Exponential spatial decay models accurately predicted site-specific open-water contact (area under the receiver operating characteristic curve = 0.87) and tap or borehole usage (0.92). There was no evidence of tap or borehole usage influencing open-water contact. Incorporating mobility terciles did not improve simple spatial decay models. Integrating spatial decay-based estimates of open-water-site usage into an individual-based transmission model produced realistic estimates of one-year *Schistosoma mansoni* reinfection and provided a ranking of water sites contributing to transmission. Our spatial decay models offer scalable tools for focal interventions for schistosomiasis.

## Abstract (web summary)

Using granular location data collected from 452 individuals in rural Uganda, this modelling analysis characterizes water contact behaviour among 69 open-water sites and 32 public taps and boreholes and reconstructs estimates of the yearly transmission of schistosomiasis.

## Main

Schistosomiasis is caused by a waterborne parasitic flatworm and estimated to currently be present in over 250 million people globally, with most cases in rural sub-Saharan Africa^1^. In endemic areas, households rely on unprotected, open-water sites due to the lack of safe water sources such as taps, boreholes or piped water^2^. Freshwater bodies, including rivers, streams and lakes, are used for swimming, drinking water collection and laundry, with each activity conferring different risks of infection and reinfection through varying cercarial exposure^3^. Mass drug administration (MDA) with praziquantel alone is recognized by the World Health Organization (WHO) and the WHO Regional Office for Africa as insufficient for reducing schistosome transmission^4,5^. Focal interventions to complement MDA are needed, yet granular spatial data are scarce and models that incorporate such information for targeting are unavailable.

Our current understanding of focality in human contact with open-water sites is limited by conventional assumptions that assign households to sites. Despite open-water contact being heterogeneous within villages and households^6,7^, it is commonly assumed that people use only the open-water site closest to their household^2,8^ or only those within their village^9^. Beyond this, several studies have suggested a direct correspondence between human mobility and open-water contact^10–12^, but it remains unclear how mobility shapes spatial patterns of site usage and whether more realistic assignment rules can be formulated beyond simply nearest distance alone.

The main focal intervention complementary to MDA and endorsed by the 2022 WHO schistosomiasis control guidelines^13^ is the provision of safe water, sanitation and hygiene (WASH). Evidence for WASH remains mixed; a meta-analysis of 39 observational studies suggested that access to safe drinking water was associated with 0.53 lower odds of schistosome infection^14^. However, the study authors noted inconsistent WASH definitions across studies and did not focus on reinfection, limiting the ability of the analysis to untangle WASH influences from other confounders, such as natural behavioural tendencies or participant characteristics, including socioeconomic status^14^. In contrast, a cluster randomized trial in Zanzibar reported no additional benefit of behaviour change (the provision of urinals and health education) and WASH provision (safe washing platforms), when combined with biannual MDA, compared with MDA alone^15^. As detailed measures of open-water and WASH usage were not collected, it remains challenging to determine why the intervention had no added benefits to biannual MDA. Most existing studies on water contact and WASH rely on self-reported information or household distance to sites and taps^2,8,16–18^ and therefore lack the objective, spatially granular information needed to characterize fine-scale water usage patterns and quantify the impact of WASH on water contact and (re)infection^19^.

Individual-based models (IBMs) have been developed to progress towards more focal interventions that account for heterogeneity in human behaviour. However, available IBMs for schistosome transmission, such as SCHISTOX^20^ and SchiSTOP^21^, typically use average, age-group-specific water contact rates without spatial structure, despite demonstrated heterogeneity by waterbody distance, gender and occupation^2,22,23^ and evidence that such heterogeneity drives variation in infection prevalence^24^. Existing IBMs therefore lack integration of human contact events at specific open-water sites with site-level environmental data, limiting their utility for targeting interventions to sites that sustain transmission.

To bridge these data and modelling gaps, we collected wearable global positioning system (GPS) logger data on 452 children and adults in the Pakwach and Buliisa districts of western Uganda and the Mayuge District in eastern Uganda, nested within the SchistoTrack cohort^25^, where *Schistosoma mansoni* is highly endemic (prevalence = 43%^2^). We studied open-water and tap or borehole usage over a ten-day period by combining GPS logger data with exhaustive mapping of all water sites used by the study communities, including those outside the study villages. The main aim was to model open-water contact and tap or borehole usage with fine-scale spatial data to develop assumptions about which individuals have contact with which open-water sites, and to validate these assumptions using spatially explicit IBMs of schistosome reinfection. A secondary aim was to evaluate whether models that incorporate WASH usage outperform models using spatial information alone in predicting human water contact at open-water sites.

## Results

### Patterns of open-water contact and tap or borehole usage

**Fig. 1:**
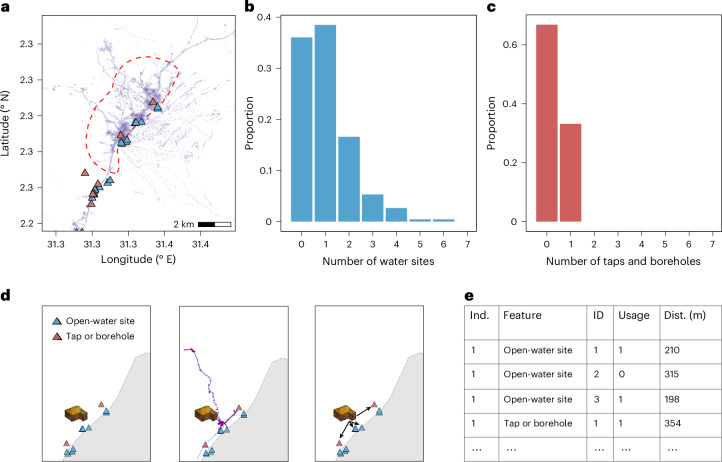
GPS logger data and processing. **a**, GPS logger data traces in Pakwach, aggregated across the study sample. GPS locations and movement are represented as purple lines). The blue triangles denote open-water sites mapped by the study team. The red triangles denote taps and boreholes. The red dashed line shows the study area. **b**, Number of distinct open-water sites visited by the study participants. **c**, Number of distinct taps and boreholes visited by the study participants. **d**, Processing steps to derive open-water-site and tap or borehole usage data from GPS data and household and site location data. **e**, Schematic of the data frame resulting from the processing, which was used to fit spatial decay models. The columns represent the unique individual identifier (Ind.), type of water feature (open-water site or tap or borehole), water feature ID, whether the individual had any contact with the feature during the observation period (1 = yes and 0 = no) and Euclidean distance (Dist.) to the feature from the household location of the individual. Uganda waterbodies shapefile from World Resources Institute (https://www.wri.org/)^70^. House icon adapted from Firkin/Openclipart.

We derived indicators of open-water contact and tap or borehole usage, as shown in Fig. 1. Within our study catchment, 143 mapped open-water sites (including adjacent sites within larger waterbodies) and 63 taps or boreholes (including distinct facilities located next to each other) were clustered into 69 distinct open-water sites and 32 tap or borehole locations. Open-water site usage or contact was defined as at least one GPS-inferred contact with mapped, shallow open-water sites (<2 m maximum depth), as a proxy for human schistosome exposure at locations with the highest parasite density^2,26^. Tap or borehole usage was defined as proximity (<20 m) to public taps or boreholes, comparable to buffer sizes used in other GPS logger studies (20–30 m)^27,28^.

A total of 8,249 open-water contact events were observed across the three districts. Overall, 63.9% (289/452) of participants visited at least one open-water site; the median daily duration was 2 min (interquartile range (IQR) = 0–12.3 min) across all participants and 9 min (IQR = 3–22 min) among the 289 individuals with any contact, who typically visited one distinct site (IQR = 1–2). The proportion visiting at least one open-water site varied substantially by district (*χ*^2^ = 75.9; d.f. = 2; *P* < 0.01), with 52.5% (96/183) in Pakwach, 89.6% (147/164) in Buliisa and 43.8% (46/105) in Mayuge. Diurnal patterns also differed by district; in Pakwach, open-water contact peaked at 08:00, whereas in Buliisa and Mayuge it peaked around 19:00 (Supplementary Fig. 1a).

We observed 1,181 tap or borehole usage events, 85.7% fewer than open-water contact events. Overall, 33.2% (150/452) of participants visited at least one tap or borehole. The proportion of participants using at least one tap or borehole was 49.4% (81/164) in Buliisa, 25.1% (46/183) in Pakwach and 21.9% (23/105) in Mayuge. The median daily duration of tap or borehole usage was 0 min (IQR = 0–1 min) across all participants. Among users, the median daily duration was 3 min (IQR = 1–10 min) and the median number of distinct facilities visited was 1 (IQR = 1–1). Tap or borehole usage peaked between 17:00 and 19:00 across all districts (Supplementary Fig. 1b). Costs were consistent across districts: borehole usage cost 1,000 Ugandan shillings (UGX; equivalent to ~US$0.27) per household per month and taps cost 100 UGX (~US$0.03) per jerrycan.

Overall, 24.3% (110/452) of participants visited both open-water sites and taps or boreholes, and 27.2% (123/452) visited neither. Usage of open-water sites and taps or boreholes was weakly positively correlated (Spearman’s rank correlation, *ρ*_s_ = 0.14; *P* < 0.01), as were usage durations (*ρ*_s_ = 0.16; *P* < 0.01). However, among participants with either open-water site or tap or borehole usage only (329/452), there was a negative correlation between the two durations (*ρ*_s_ = − 0.14; *P* < 0.01).

### Association of open-water contact with schistosome infection outcomes

At baseline, 47.6% (215/452) of participants were infected with *S. mansoni* based on Kato–Katz microscopy; after treatment with praziquantel, 17.0% (77/452) remained positive four to five weeks later, with 40.5% (183/452) reinfected at one-year follow-up. Median infection intensities among Kato–Katz-positive individuals were 96 eggs per gram (EPG; IQR = 36–264) at baseline, 36 EPG (IQR = 12–120) after treatment with praziquantel and 48 EPG (IQR = 24–126) at one-year follow-up. Full infection intensity data, including median EPG values at each time point, are reported in Supplementary Sections 1.1 and 1.2 and Supplementary Fig. 2. Duration of open-water contact was positively correlated with both baseline and one-year infection intensity (*ρ*_s_ = 0.18 (*P* < 0.01) and *ρ*_s_ = 0.20 (*P* < 0.01), respectively). Duration of tap or borehole usage was not associated with baseline infection intensity (*ρ*_s_ = 0.03; *P* = 0.46) and was only weakly positively correlated with one-year infection intensity (*ρ*_s_ = 0.10; *P* = 0.04).

### Associations of age, gender and household distance with open-water contact

Participants ranged in age from 5–82 years and were 50% female (226/452). Age and gender had no significant effect on open-water site or tap or borehole usage (Supplementary Table 1). Occupation was relevant as fishermen and fishmongers were more likely to use open-water sites. Household access to an improved drinking water source was positively associated with both open-water and tap or borehole usage. Open-water contact peaked around age 25 years, whereas tap or borehole usage was largely stable across age (Supplementary Fig. 3). Within households where both a child and an adult participated, open-water contact and tap or borehole usage were each correlated between the two individuals (*ρ*_s_ = 0.36 (*P* < 0.01) and *ρ*_s_ = 0.34 (*P* < 0.01), respectively; *n* = 191).

Households in these fishing villages had a median distance of 219 m (IQR = 88–406 m) to the nearest open-water site and 335 m (IQR = 181–663 m) to the nearest tap or borehole. Fishermen lived significantly closer to open-water sites (median = 84 m; IQR = 58–190; *n* = 41) than other adults (median = 233 m; IQR = 74–400; *n* = 186; Wilcoxon rank-sum test statistic, *W* = 2,599; *P* < 0.01). Individuals travelled slightly further to visit open-water sites (median = 229 m; IQR = 109–416 m) than taps or boreholes (median = 172 m; IQR = 123–320 m), although the difference was not statistically significant (*W* = 37,666; *P* = 0.15). The nearest open-water site accounted for 47% (219/466) of all distinct sites visited and 55% of total contact duration (43,619/79,296 min), whereas the nearest tap or borehole accounted for 99.3% (149/150) of all distinct facilities used and 99.8% of total usage duration (16,417/16,447 min).

### Association of human mobility with open-water contact

**Fig. 2:**
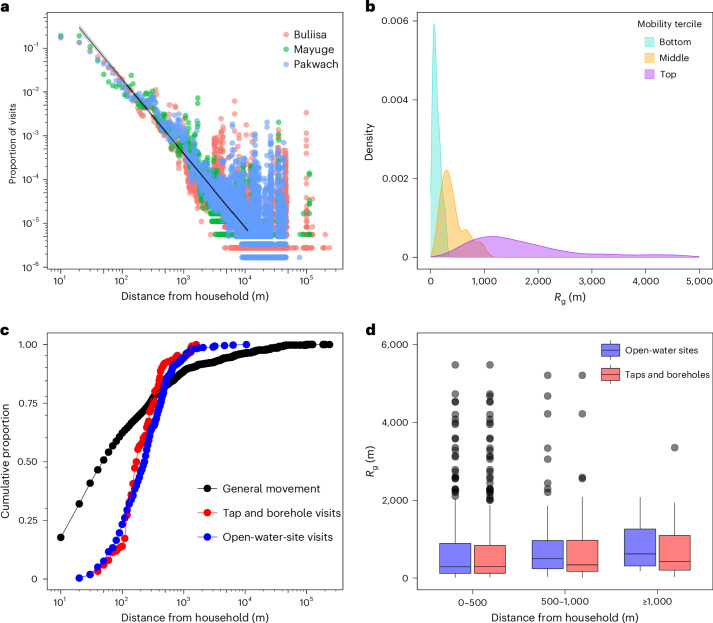
Association between mobility and usage of open-water sites and taps or boreholes. **a**, Proportion of visited locations over distance from the household. The black line represents a truncated power-law distribution fitted between 1.3 × 10^1^ and 1.1 × 10^4^ m with a 95% CI (shaded area). Limits were determined using the poweRlaw R package. **b**, Distribution of radii of gyration (*R*_g_; equation (1)) by district-specific mobility tercile. *R*_g_ measures human mobility relative to the distance from the household. **c**, Cumulative proportions of visits to open-water sites or tap and borehole locations, as well as all GPS locations (general movement), plotted against distance from the household. **d**, Relationship between *R*_g_ and household distance to the nearest open-water site or tap or borehole. Central horizontal lines and box limits represent median values and the IQR, respectively. The whiskers extend to the most extreme data points within 1.5× the IQR of each box. Outliers are plotted as individual points.

To capture the movement of individuals relative to their home location for visits to open-water sites or taps or boreholes, and for general purposes, we used the radius of gyration as our mobility metric (Methods). Aggregate mobility patterns were well approximated by a truncated power-law distribution (*R*^2^ = 0.73 within a range of 1.3 × 10^1^–1.1 × 10^4^ m), with a median radius of gyration of 356 m (IQR = 150–1,090 m; Fig. 2a). Although participants in Pakwach exhibited higher median movement than those in Mayuge or Buliisa, neither district-level nor occupational differences (for example, fishermen versus other adults) were statistically significant (Supplementary Section 1.3). We therefore grouped participants into district-specific mobility terciles for subsequent analyses (Fig. 2b). Tap or borehole visits were the most spatially focal (virtually all within 1 km), whereas open-water contacts extended to 10 km and general mobility to 100 km (Fig. 2c). Individuals living further from waterbodies (>1 km) appeared to have larger mobility radii, but this trend was not statistically significant (Fig. 2d and Supplementary Section 1.3).

### Conceptual framework of the spatial factors influencing open-water contact

**Fig. 3:**
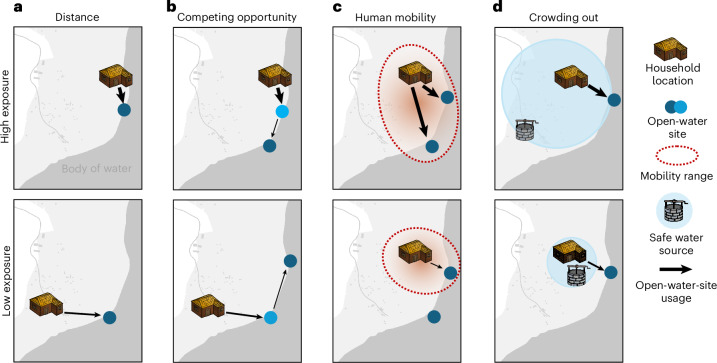
Schematic of different spatial factors influencing open-water-site usage. **a**, Open-water-site usage depends purely on household distance to the site. **b**, Open-water-site usage depends on household distance to the index site (dark blue) plus the distance to competing sites (light blue). **c**, Open-water-site usage depends on the range of human mobility. **d**, Open-water-site usage depends on the proximity to safe water infrastructure, such as boreholes, whereby boreholes are able to reduce (that is, crowd out) open-water contact. Uganda waterbodies shapefile from World Resources Institute (https://www.wri.org/)^70^ and base maps from OpenStreetMap (https://www.openstreetmap.org). House icon adapted from Firkin/Openclipart.

To identify key factors influencing open-water contact, we evaluated four spatial decay models of increasing complexity (Fig. 3). The baseline model hypothesized that open-water contact is solely determined by household distance to open-water sites (Fig. 3a). We sequentially expanded this framework to account for competing opportunities (where site distance rank influences choices between competing sites; Fig. 3b), individual mobility (Fig. 3c) and a crowding-out effect whereby tap or borehole usage reduces open-water contact (Fig. 3d). All spatial decay models were fitted as Bayesian nonlinear regression models predicting the probability of an individual using a specific open-water site or tap or borehole (equation (2) and Methods), with model selection performed using expected log pointwise predictive density (ELPD) differences.

### Spatial decay models of open-water usage

An exponential spatial decay model (Supplementary Fig. 4), corresponding to Fig. 3a, best approximated open-water-site usage, outperforming a simple logistic regression using household distance (ELPD difference = 23.7; *P* < 0.01), particularly at small spatial scales (<100 m; Supplementary Fig. 5). Distance-based models also outperformed logistic regression models using sociodemographic and behavioural proxy variables (Supplementary Table 2).

**Fig. 4:**
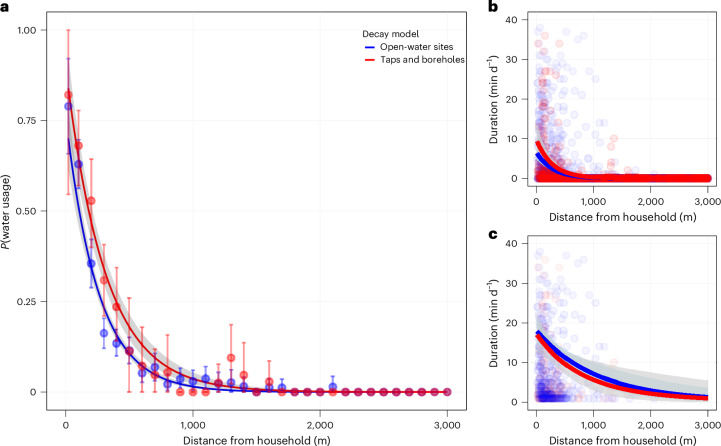
Spatial decay baseline models. **a**, Individual-level spatial decay models (referred to as global models) predicting the probability of open-water site and tap or borehole usage over household distance. The dots represent the data, with error bars indicating 95% CIs obtained via non-parametric bootstraps with replacement. The error bars represent the 2.5th and 97.5th percentile ranges, calculated using 2,000 replicates and plotted in 100-m increments. The points represent the respective means. **b**,**c**, Spatial decay models predicting the duration of open-water-site contact and tap or borehole usage over household distance among all individuals (**b**) and restricted to individuals using either open-water sites or taps and boreholes, respectively (**c**). The solid lines and shaded grey areas indicate posterior means and 95% CrIs, respectively. In **b** and **c**, the points represent the raw data.

The global spatial decay model of open-water-site usage (Fig. 4a) had a stratified fivefold cross-validated area under the receiver operating characteristic curve (AUROC) of 0.86 (95% confidence interval (CI) = 0.85–0.88). For individuals living close to open-water sites (20 m), the predicted probability of usage was 70% (95% credible interval (CrI): 61–78%), decreasing to 51% (95% CrI = 46–56%) at 100 m, 11% (95% CrI = 9–12%) at 500 m and 1% (95% CrI = 0.7–1.4%) at 1,106 m (Fig. 4a).

Tap or borehole usage exhibited a broadly similar spatial decay, although the decrease was somewhat steeper (Fig. 4a), with a higher AUROC of 0.92 (95% CI = 0.90–0.94). At 20 m, the predicted probability of usage was 84% (95% CrI = 72–93%), 14 percentage points higher than for open-water sites, decreasing to 65% (95% CrI = 57–71%) at 100 m, 18% (95% CrI = 15–21%) at 500 m and 1% (95% CrI = 0.5–1.7%) at 1,413 m, compared with 1,106 m for open-water-site contact (Fig. 4a).

We additionally used exponential spatial decay models to predict the duration of open-water contact and tap or borehole usage (Fig. 4b,c), with moderate predictive performance (*ρ*_s_ = 0.36 for open-water contact and *ρ*_s_ = 0.47 for tap or borehole usage) and similar decay rates for the duration and probability of any usage (both decay rate parameters *b*_1_ = 0.004). Given the substantial number of non-users, we refit the duration model restricted to participants with any use (*n* = 275/452 for open-water sites; *n* = 135/452 for taps or boreholes). For these individuals, decays were less pronounced and predicted durations remained positive, even at 2,000 m (Fig. 4c).

### Spatial decay models of open-water contact with additional covariates

We tested whether including additional covariates improved the ability of spatial decay models to predict open-water contact and tap or borehole usage. District-specific distance decays performed best, outperforming the respective global models (ELPD differences of 13.0 (*P* = 0.04) and 10.1 (*P* = 0.09); Supplementary Table 3), with AUROCs of 0.87 (95% CI = 0.85–0.89) and 0.92 (95% CI = 0.90–0.94) for open-water contact and tap or borehole usage, respectively (Supplementary Fig. 6). The performance of the district-specific models was similar to that of models including gender, activity match, age or safe water type as covariates (all ELPD difference *P* values > 0.05; Supplementary Table 3). We used the district-specific model (Supplementary Fig. 7) as our benchmark for all subsequently introduced models. The ability of this model to predict population-wide open-water contact patterns and site-level traffic using building footprints as a proxy for population density is demonstrated in Supplementary Section 1.9 and Supplementary Fig. 8.

### Spatial decay models of open-water contact incorporating competing opportunities, human mobility and crowding out

**Fig. 5:**
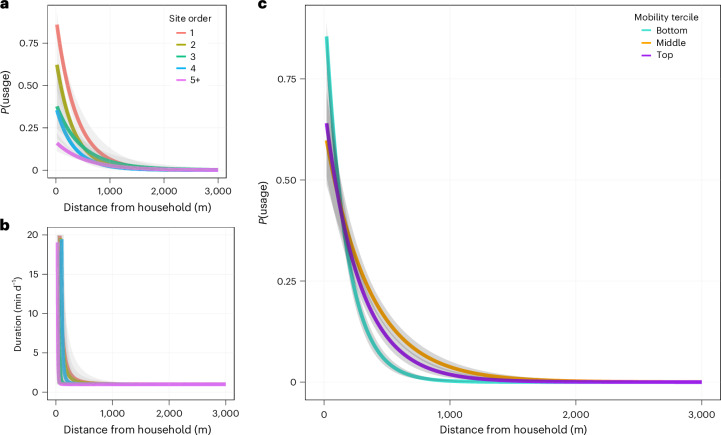
Spatial decay models of competing opportunities and human mobility. **a**, Spatial decay model of open-water-site usage based on relative distance to competing open-water sites. For each individual, all open-water sites were ranked by increasing distance from the household, where 1 is the nearest and 5 is any site farther than the fourth-nearest. A separate distance-decay function was then fitted for each rank category. **b**, Spatial decay model predicting open-water-site duration based on site order. **c**, Spatial decay model predicting open-water-site usage probability based on district-specific mobility tercile. The solid lines indicate posterior means and the shaded grey areas represent 95% CrIs.

To further refine the spatial decay model, we evaluated whether incorporating site competition, human mobility and crowding out (hypotheses shown in Fig. 3b–d) improved performance over the distance-based benchmark. The competing opportunities model used relative distance ranks alongside absolute distance, so that comparatively closer sites reduced the usage of more distant sites. This significantly improved the predictive performance over the benchmark (ELPD difference = 52.1; *P* < 0.01; AUROC = 0.87; 95% CI = 0.86–0.89), with the monotonic decay in Fig. 5a confirming that the usage probability decreased as the number of closer competing sites increased, even after accounting for absolute household distance. A simpler rank-only model without absolute distance performed substantially worse (ELPD difference = −111.6; *P* < 0.01; Supplementary Fig. 9).

We also examined whether higher mobility was associated with visiting more distant open-water sites (Fig. 3c). Individuals in the middle mobility tercile maintained the highest open-water contact probabilities at greater distances, whereas those in the high and low terciles showed steeper decays (Fig. 5c). The mobility-adjusted model did not significantly outperform the benchmark for either open-water contact (ELPD difference = 14.2; *P* = 0.20) or taps or boreholes (ELPD difference = −11.6; *P* = 0.07).

**Fig. 6:**
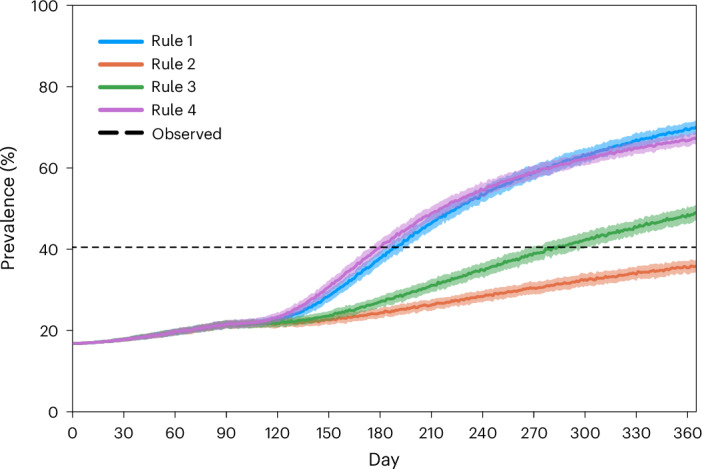
Predicted infection prevalence based on four different open-water-site visitation rules. The approximate start of the simulations is the post-treatment infection prevalence (16.9%). The dashed black line indicates the reinfection prevalence (40.5%) observed one year after baseline. The shaded areas represent 95% CIs, calculated based on 100 replicates for each decision rule for 365 days. The main text provides detailed information on each rule.

The crowding-out model failed to outperform the benchmark for either site usage probability (ELPD difference = −2.4; *P* = 0.22) or duration (ELPD difference = −0.02; *P* = 0.99). Even in a counterfactual scenario assuming this unsupported model held true, we estimated that <2% of individuals (95% CrI = 0–5%) would cease open-water contact due to tap or borehole usage, with a maximum population-level reduction in open-water contact duration of 23% (95% CrI = 19–37%) under universal adoption, and no significant variation in crowding out by contact type (occupational, domestic or recreational; Supplementary Section 1.6 and Supplementary Figs. 10 and 11).

### Model robustness checks

We assessed the minimal data needs and robustness of our main (district-specific open-water contact) model to GPS recording frequency and buffer size. As few as 15 individuals recorded over ten days were sufficient to estimate the spatial decay (Supplementary Fig. 12a), and the decay parameters *b*_0_ and *b*_1_ were robust to removing contact events of <5 or <10 min duration, respectively (Supplementary Section 1.7 and Supplementary Fig. 13). Using buffer sizes of 20 and 30 m yielded similar results (Supplementary Fig. 14). Males appeared somewhat less compliant in wearing the GPS loggers than females (*P* = 0.01; Supplementary Section 1.8 and Supplementary Table 5) and the district-specific model was modestly less accurate for fishermen and adults (*P*< 0.01 and *P* = 0.04, respectively; Supplementary Section 1.10 and Supplementary Table 6). Despite this, the global model, although not the best-performing model, showed high transportability across districts when each was held out in turn (AUROC = >0.82; Supplementary Fig. 12c).

### Using model-based open-water site contact probabilities to inform open-water contact in IBMs

We incorporated a spatial decay function of open-water contact into an IBM (Methods), having confirmed that model-based estimates of open-water contact were significantly correlated with baseline infection intensity (*ρ*_s_ = 0.18; 95% CI = 0.09–0.26) and one-year infection intensity (*ρ*_s_ = 0.20; 95% CI = 0.11–0.27; for validation of GPS-derived against self-reported open-water contact; Supplementary Section 1.4).

Using district-specific decay model parameters, we investigated four visitation rules governing individual contact with open-water sites within the IBM to understand how the choice of visitation rule influenced predictions of infection one year after treatment (Fig. 6). The rules, spanning a range of assumptions about how individuals distribute contacts across sites, were: (1) visit the nearest site with 100% probability; (2) visit the nearest site with probability *P* from the spatial decay model; (3) apply Bernoulli trials with probability *P* across all sites and visit one success at random; and (4) follow the same rule as (3) but visit all sites assigned a success.

**Extended Data Fig. 1:**
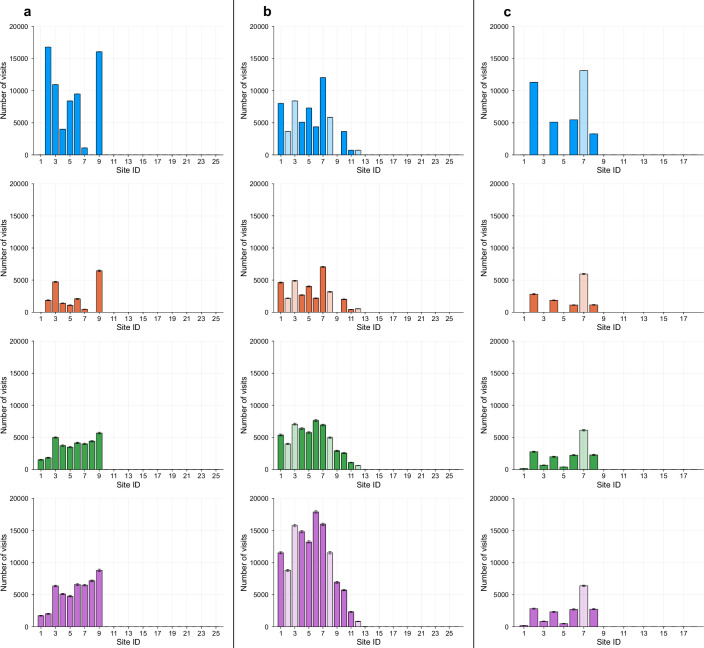
Predicted total number of visits per open-water site over 365 days for each district and visitation rule. The panels show the median predicted total number of open water site visits over 365 days and 100 replicates across the districts of Pakwach (**a**), Buliisa (**b**), and Mayuge (**c**) under the four different visitation rules (blue-rule (1), orange-rule (2), green-rule (3), and purple-rule (4)). Each vertical bar represents one open water site with the x-axis label denoting its site ID. Lighter-coloured bars correspond to open water sites where no snails were observed during snail sampling, and which consequently do not contribute to transmission. Error bars represent the 2.5th and 97.5th percentile range across replicates. The narrow intervals indicate low between-replicate variability in predicted site visitation. Note that rule (1) is deterministic, so between-replicate variability for this rule is zero, and no error bars are shown.

The IBM yielded the total number of visits for each site over a one-year simulation period (Extended Data Fig. 1). There was between-district heterogeneity in the percentage of used open-water sites with a presence of *Biomphalaria* intermediate hosts ranging from 63.6% (Buliisa) to 100% (Pakwach), with no obvious relationship between the number of contacts and snail presence. Rules (3) and (4) predicted more sites being used than rules (1) and (2), which restricted visitation to the nearest site only. More than half of all mapped sites were predicted to be unused under all rules (Supplementary Section 1.5).

We quantified the human-to-snail transmission input by estimating cumulative miracidial contamination introduced through human water contact and expressed this as a percentage of the most contaminated site per district (Supplementary Fig. 15). Contamination distributions broadly mirrored visit distributions, although site rankings occasionally differed between the two metrics, indicating that high visit counts do not always correspond to the highest transmission input.

The IBM predicted a one-year post-treatment infection prevalence of 70.1% for rule (1), 35.8% for rule (2), 49.0% for rule (3) and 67.2% for rule (4), with rules (2) and (3) closest to the observed prevalence of 40.5% (183/452). Sensitivity analyses on the aggregation parameter (Supplementary Fig. 16) showed that the ordering of rules was robust, although differences between rules narrowed at lower parameter values, which are more appropriate for low-prevalence settings than ours.

## Discussion

The WHO schistosomiasis guideline emphasizes the need for spatially focal interventions to reduce schistosome exposure in endemic areas^13^. We studied open-water site contact and tap or borehole usage patterns of 452 children and adults in rural Uganda over a ten-day period, identifying sites with the highest probability of usage and frequency of contact. Spatial decay models of open-water contact, constructed from wearable GPS logger data, showed high predictive performance and informed more realistic assumptions in IBMs of schistosome transmission.

Individual-level spatial decay models informed by household distance and district alone predicted open-water contact and tap or borehole usage well (AUROCs of 0.87 and 0.92, respectively), and a simpler purely distance-based model still had high transportability across diverse Ugandan districts (AUROC > 0.82). This contrasts with previous studies using aggregate cell-phone mobility data, which found that gravity models (spatial decay models similar to ours) performed poorly in rural settings^29,30^. Previous work predicting open-water contact used coarse mobile phone data^10,12^, no mobility data^11,19^ or small GPS logger samples^27,31^. Our models demonstrate that simple exponential spatial decay functions can accurately approximate fine-scale water contact patterns relevant to schistosomiasis. A similar decay has been proposed for water usage in trachoma^32^ but not rigorously evaluated, highlighting the need for future research on whether spatial decay models generalize to other environmentally mediated infectious diseases.

Integrating spatial decay into schistosome transmission models reflected the biological realism of complex human water contact^2^. Previous theoretical work has shown that schistosome transmission depends not only on the total number of open-water contacts, but also on their distribution across sites^33^. We demonstrated that this distribution can be approximated using simple household-to-site assignment rules learnt from wearable GPS loggers. Rules informed by spatial decay probabilities and restricted to a single site visit per day produced one-year post-treatment infection prevalence estimates closest to the observed prevalence, outperforming the most common spatial assumption of assigning all contacts to the nearest open-water site^8,16^. Incorporating space explicitly therefore captures the heterogeneity and focality of human exposure, providing a more reliable foundation for understanding district-level infection patterns.

Our transmission model provides the information needed for a localized decision framework targeting focal interventions at both humans and snails. Combining site-specific visit counts with data on snail presence and miracidial contamination allows for directly inferring transmission potential at each site and identifying priority sites for intervention depending on the degree of heterogeneity. Highly skewed contamination distributions support focal mollusciciding^34^, other biocontrol methods^35^ or intensified snail surveillance, whereas more uniform distributions suggest the need for broader coverage. A mismatch between visit distributions and miracidial contamination at the site level can identify high-burden individuals for test-and-treat strategies^36^ and facilitate a comparison of human and snail forces of infection to determine whether the same sites drive both sides of the transmission cycle.

The special decay model results suggest that safe water provision alone is unlikely to eliminate open-water contact. Crowding-out models did not outperform purely distance-based models, and even under counterfactual scenarios assuming crowding out existed, fewer than 2% of participants would completely divert their open-water contact to taps or boreholes. This aligns with Knopp et al.^15^ and the WHO guideline^13^, which anticipate only modest WASH effects, rather than the larger reductions implied by observational meta-analyses^14^. Further experimental work on WASH delivery models (household versus public provision)^37^, infrastructure types (boreholes, standpipes, water kiosks and so on) and cost structures is needed to identify conditions under which WASH might meaningfully divert high-risk water contact activities, even if total open-water contact is not substantially reduced.

Despite interest in identifying mobile individuals who may be missed by MDA programmes^38,39^, we found few clear sociodemographic correlates of human mobility. Adults had larger movement ranges than children, although median mobility did not differ significantly, and fishermen were not found to be more mobile than other adults, in contrast with previous suggestions^40^. Spatial decay models incorporating mobility did not outperform simpler distance-based models, indicating that mobility patterns cannot serve as a direct proxy for open-water contact^10,11^.

Spatial decay models have practical applications across multiple elements of schistosomiasis control^13^. As essentially all open-water contact and tap or borehole usage occurred within 1 km of the homes of individuals, exposure reduction should focus locally, limited to home and neighbouring villages. Our models can predict the number of people using each open-water site, guiding focal mollusciciding of the most visited sites^41^. Notably, radius control should extend to 1 km from households, larger than the 500 m suggested by the WHO^41^, to cover all locally relevant sites. GPS logger data from as few as 15 individuals over ten days were sufficient to estimate spatial decay, demonstrating logistical feasibility. We also showed that publicly available building footprints or WorldPop data^42,43^ can approximate population-level open-water contact, although generalizability will depend on local infrastructure, social norms and the representativeness of the GPS sample and observation season.

The strengths of our study include: the large-scale collection of wearable GPS logger data; extensive model investigation, including transportability across districts; proposed theories of change for spatial decay models; and validation with reinfection data and spatially explicit IBMs. Our study has several limitations. We focused on the spatial dynamics of open-water contact and did not model temporal patterns beyond descriptive reporting of diurnal usage. The ten-day observation window may not be representative of general usage patterns and models may not generalize to the rainy season when open-water site distributions and accessibility differ^26^. However, the dry season focus captures a key transmission period when contact is highest^2,16^. As open-water contact is inferred from GPS logger positions and not directly observable, there is a risk that our estimates of open-water contact have noise (although we have no reason to believe they are systematically biased). From the qualitative data we collected, we did not receive any reports (although these were not elicited in a structured manner) that individuals changed their routine behaviours while wearing the loggers. We removed the first day of observation to guard against Hawthorne effects and found that decay parameters were robust to the exclusion of short-duration contact events (<5 min). Our 20 m buffer was stringent relative to previous studies^27,31^, yielding conservative contact estimates. Nonetheless, the observed median daily contact duration of 3 min is consistent with an observational study of 120,000 open-water contacts in Senegal (4.3 min)^22^ and another study from Kenya showing that the average number of open-water sites typically visited by people was less than one^23^. As our study mapped open-water sites up to 26 km away, and with >99.5% of all open-water contact occurring within 3,000 m, unmapped distant sites are unlikely to have affected the findings, although some local contacts may have been missed if sites were not captured during mapping. All study communities were within 2 km of waterbodies and our findings may thus not apply to communities further from the shoreline. Spatial decay models assign identical predictions to all household members, limiting the investigation of intra-household dynamics, but incorporating sociodemographic covariates did not improve model performance over a purely spatial model. Due to the nature of the GPS logger data, we had no information on specific activities; activity-level data are better captured through surveys or direct observations^2,16^. Validation of GPS-derived against self-reported open-water contact yielded a weak positive correlation, partly reflecting imperfect alignment between the GPS observation period and survey recall window. Future studies should use directly comparable measurement periods.

In conclusion, we provide individual-level spatial decay models of human water contact relevant for modelling schistosome transmission. Future research should assess whether similar models generalize to other waterborne diseases, recognizing pathogen-specific exposure pathways, and should collect GPS logger data across diverse geographical settings to establish the broader generalizability of observed spatial decays. With further evaluation, our models could serve as planning tools for targeting focal interventions, including WASH, mollusciciding and MDA prioritization.

## Methods

### Ethical approval

Data collection and use were reviewed and approved by the Oxford Tropical Research Ethics Committee (509-21), Vector Control Division Research Ethics Committee of the Uganda Ministry of Health (VCDREC146) and Uganda National Council for Science and Technology (HS 1664ES). Written informed consent was obtained for all study participants, with adults consenting on behalf of children and children providing verbal assent.

### Study context

In sub-Saharan Africa—the region with the lowest access to safe drinking water—408 million people rely on unsafe sources such as rivers, lakes and ponds^44^. This study focuses on Uganda, where <10% of people in rural areas are estimated to have access to safely managed drinking water^44^ and where the national prevalence of schistosomiasis has been estimated to be 26% using point-of-care circulating cathodic antigen tests^18^. Despite over 13 rounds of MDA since 2003, repeated MDA in Uganda has failed to achieve the WHO targets of morbidity control and elimination as a public health problem^45^. The 2022 WHO schistosomiasis guideline highlights the need for multifaceted control, such as combining MDA with behaviour change and WASH, to achieve the WHO 2030 control targets^13^. SchistoTrack collected comprehensive environmental, behavioural and spatial data to inform more integrated, focal schistosomiasis control programmes. The study focused on moderate- and high-endemicity villages in western and eastern Uganda, where the prevalence of schistosomiasis, as measured by stool microscopy, was 43%^2^. The study took place across three Ugandan districts, Pakwach, Buliisa and Mayuge, which are located along the River Nile, Lake Albert and Lake Victoria, respectively^25^. These areas were selected to represent diverse climates (tropical rainforest and tropical savannah climates), waterbody types (lake and river settings) and tribal and religious groups. All villages were <2 km away from waterbodies, and people engaged primarily in subsistence farming or agriculture, with 47% reporting open-water contact in the past week^2^.

### Study design and participant sampling

The GPS logger study was nested within the SchistoTrack cohort study baseline, which took place between January and February 2022 and enrolled 1,459 households across 38 villages (*n* = 2,885), approximating 40 households per village^2^. Participants were randomly selected from village registers or MDA records (see Puthur et al.^25^ for details). Of those 38 villages, 12 (four in each district) were selected for the GPS logger study based on their levels of open-water contact and occupational fishing, determined predominantly by the presence of a beach or landing site. None of the selected villages had piped water (that is, individuals had no access to safe drinking water within their households). A maximum of 50 participants from 25 households per village were selected among SchistoTrack participants, with a target of one adult–child pair (with adults defined as people ≥18 years of age and children defined as those 5–17 years of age) per household^25^. This study excluded children aged <5 years, as there was no approved paediatric praziquantel formulation for this age group at the time of the study. For this nested cross-sectional study, SchistoTrack participants were sampled to ensure a balance of age and gender, and fishermen were oversampled to ensure a diverse representation of open-water contact and mobility patterns. We excluded participants who were physically immobile, as defined by the participant, and thus unable to have open-water contact. We also excluded children who were sent away to boarding school and were unavailable during the GPS logger study period. All participants were unaware of their schistosome infection status when they entered the GPS logger study.

### Household survey data

Before the GPS logger study, questionnaires to collect sociodemographics, biomedical variables, WASH and environmental variables and self-reported open-water contact patterns were administered to the household head of all 1,459 households that were part of the larger SchistoTrack study. Questionnaires were translated into local languages and administered digitally by two surveyors (one recruited centrally from Kampala and one recruited from within the district) trained by G.F.C. and M.T.E. The data were collected via a tablet device (Lenovo TB-8505F) and the Open Data Kit platform (using Open Data Kit Collect application version 2022.4). To ensure the quality of the survey data, questionnaires were piloted using field tests and mock interviews; response constraints were implemented to avoid implausible response values as far as possible; and standardized interview protocols were used to ensure consistent interview procedures were followed by surveyors. At the end of the household interview, one adult and one child per household were selected for clinical assessments by the household head. From these clinical participants, a subset was subsequently selected for inclusion in the GPS logger study.

### Community sensitization, consent and remuneration

G.F.C., M.T.E. and F.R. conducted community workshops in each village to explain the study objectives and address any questions or concerns. As part of the standard written informed consent for recruitment into SchistoTrack, consent was obtained from adult participants and parents or guardians for children under 18 years in the local language, specifically for the GPS logger study. Children provided verbal assent and, where possible, written assent was also collected. Participants received a nominal remuneration equivalent to about one day of wages (10,000 UGX; ~US$2.73) in addition to any incentive in the study upon returning their GPS loggers at the end of the observation period.

### GPS logger settings and procedures

Wearable GPS loggers (i-gotU GT-600; Mobile Action Technology, Taiwan) were used to record the location data of participants at two-minute intervals between 05:00 and 20:00 local time over approximately ten consecutive days. This time window was chosen to capture activities during the day. Loggers were turned off from 20:00–05:00 to save battery, using scheduled control, as loggers were not recharged in the field by participants or the study team. Also to save battery, all loggers were set to motion activation such that loggers turned off when participants did not move. Button controls on devices were disabled, so participants were unable to switch them off. Devices were placed in waterproof pouches and worn around the neck using a lanyard (Supplementary Fig. 17). Children and adults in the same household always received different-coloured lanyards to minimize the risk of accidentally mixing up the loggers. Adults were asked to help children with the day-to-day wear of the loggers. Participants were instructed to wear the devices during all daily activities, including during open-water contact, and to remove them only at night.

### Participant flow

A participant flowchart is shown in Supplementary Fig. 18. A total of 215 loggers were purchased for the study, with the aim of distributing 200 loggers across three districts sequentially (that is, 600 in total).The total number of recruited participants was 585—slightly below the target of 600. This was mainly due to device failure and unreturned loggers in Pakwach. After excluding participants with fewer than two complete days of GPS data—excluding the first day of recording for all participants to reduce the Hawthorne effect^46^—we included a final analytic sample of 452 participants in the study.

### GPS logger testing and validation

We tested the accuracy of GPS loggers in situ. During fieldwork, ten loggers were placed next to each other on the ground for 10 min in an unobstructed area in Buliisa to estimate GPS position error in the field. Based on this experiment, we estimated that GPS loggers were accurate within 8.6 m. GPS precision was estimated using the uere function in the ctmm package in R^47^. Our estimate of 8.6 m aligned with a previous study, which estimated the accuracy of i-gotU GT-120 under different levels of obstruction and found that the mean location error was <10 m and that substantial cover moderately increased the location error by 2.2 m^48^.

### GPS logger data retrieval

For 94 participants, no GPS data could be retrieved as loggers suffered water damage despite the use of protective pouches and the manufacturer’s indication that devices were fully waterproof. Multiple retrieval strategies were employed: metal contacts were cleaned with ethanol in the field; loggers were transported to Oxford for a second cleaning attempt; and 36 devices with persistent data loss were opened at the University of Oxford Materials Science Department, where a research technician resoldered the GPS chips onto functioning logger boards. This recovered data from 24 of the 36 affected loggers.

### Self-reported wear compliance

Wear comfort was assessed when participants returned GPS loggers. As not all devices were returned immediately following the end of the study period by the participants themselves, these data were available for 87% of participants (395/452). Among those participants, 83% (328/396) reported that wearing the logger was comfortable. Other participants self-reported that loggers caused perceived chest pain (34), body weakness (5), nausea (1) or headaches (1), which were addressed through sensitization.

### Mapping of village infrastructure, open-water sites and taps and boreholes

As part of the study, F.R., M.T.E. and (initially) G.F.C. comprehensively mapped the locations of all public taps and boreholes used by the study communities that were relevant for schistosome transmission, assisted by the village chairman or a village health team member. As part of this mapping, the type (tap or borehole), GPS location and state of each facility were assessed. Trained malacologists from the Division of Vector-Borne and Neglected Tropical Diseases at the Uganda Ministry of Health mapped all open-water sites used by study communities, including sites outside village perimeters but accessed by community members with the help of local guides, such as village chairmen or community health workers. This comprehensive mapping approach ensured the inclusion of all relevant open-water sites used by the study population, regardless of their proximity to residential areas, as is described in detail by Iacovidou et al.^26^.

### Deriving open-water contact and tap or borehole usage patterns

A contact event with an open-water site and tap or borehole was defined as being within a 20 m buffer of the GPS location of an open-water site or tap or borehole. We chose 20 m to account for possible GPS position errors while minimizing overestimation of open-water contact. This buffer was conservative compared with previous GPS logger studies; Eyre et al.^27^ used a 30 m buffer around the shoreline, whereas Seto et al.^31^ used a 100 m buffer around the shoreline. We used a larger buffer of 30 m as a robustness check, with the results reported in Supplementary Fig. 14. GPS locations of sites and taps or boreholes were recorded via tablet (Lenovo Tab M8 (3rd Gen); Lenovo Group; China) with 5–10 m manufacturer-reported accuracy, and GPS logger points were reported with 8.6 m accuracy, as described above. All contact events with open-water sites and taps or boreholes were identified using recursion analysis—a well-established method in movement ecology^49,50^—and implemented in the recurse package in R^51^. The output from the recursion analysis was a list of all distinct open-water contact and tap or borehole usage events with an associated timestamp, number of revisits, contact duration and open-water site and tap or borehole identifier (Fig. 1e). Compared with simply counting the number of points within each buffer area, recursion analysis has the advantage of reconstructing an approximate linear trajectory. It can therefore identify visits with durations shorter than the GPS logger sampling frequency. This is particularly important given that water contacts are typically short^22^.

As the SchistoTrack protocol did not allow sites to be larger than 15 m, even when they were directly adjacent, we grouped such nearby locations into a single open-water site (or a single tap or borehole for adjacent taps and boreholes) for the analysis, following the methods used by Iacovidou et al.^26^. The density-based spatial clustering of applications with noise algorithm was used and the optimal number of clusters was determined based on the gap statistic, separately by district^52^. This collapsed the 143 open-water sites and 63 taps and boreholes into 69 and 32 clusters, respectively, to which we then assigned the mean GPS cluster coordinates.

### Sanitation infrastructure

This analysis did not focus on the use of public latrines, even though it would have been possible to derive this information from the GPS data using the same methods as for open-water site and tap or borehole usage. There were two reasons for this. First, field visits to all public latrines by M.T.E. and F.R., and initially by G.F.C., revealed that most were locked (usually a community member held the keys) and not regularly used by the community. Second, among the 452 GPS logger participants, 343 (75.9%) lived in households that had a private latrine (that is, a flush or pour-flush toilet, a covered pit latrine with or without privacy or a composting toilet). Thus, most households could rely on private latrines, which provide more privacy and convenience compared with public latrines^53,54^. However, private latrine usage events could not be derived from GPS logger data due to the unknown precise location of these latrines.

### Dataset and variables

#### Dataset

The dataset used to estimate spatial decay models was generated by calculating the Euclidean household distance to all open-water sites and taps or boreholes (both used and unused) within the same district (Fig. 1e). This covered distances of up to 11, 17 and 26 km for Pakwach, Buliisa and Mayuge, respectively.

#### Outcomes

For the models estimating any usage of open-water sites or taps or boreholes, the outcome was a binary indicator of whether an individual used an open-water site or a tap or borehole. For the model estimating duration of usage, the outcome was a continuous variable indicating the number of minutes per day each individual had contact with each mapped open-water site or tap or borehole. Duration, in minutes per day per individual, was calculated by dividing the total duration of contact with each open-water site or tap or borehole by the number of distinct calendar days with GPS data for each individual.

#### Covariates

The main predictor variable was household distance to each open-water site or tap or borehole. We selected additional predictor variables based on their relevance for open-water contact. The following individual-level variables were used: age, gender, occupation, self-reported open-water contact, water contact activity match and mobility tercile. Age was coded as a binary variable (<15 or ≥15 years). This cut-off was chosen based on our previous analysis of the SchistoTrack data, which demonstrated that open-water contact levels were low in individuals aged <15 years and increased substantially thereafter, suggesting that 15 years of age was a natural cutoff^2^. We also used gender (male or female), reported by the household head, as a covariate. Individual-level occupation was also used as a covariate because occupation is an important determinant of open-water contact^2^. The household head (aged ≥18 years) reported the occupation of all household members. The occupation categories were fishing, fishmongering, farming and other. A self-reported open-water contact variable, described in detail in Reitzug et al.^2^, was also included. A binary open-water contact activity match variable was generated for each individual–site pairing. Whenever an individual conducted any of the 11 domestic, recreational or occupational open-water contact activities, and whenever the same activity was reported to be performed at a specific site, based on reports by the local guide, this was defined as an activity match. The 11 open-water contact activities included: collecting drinking water; washing clothes with soap; washing clothes without soap; bathing with soap; bathing without soap; washing jerry cans or household items; collecting papyrus; fishing; fishmongering; collecting shells; and swimming or playing. All individuals who reported collecting drinking water were also assigned to having an activity match with taps or boreholes, as this activity was amenable to being conducted at a tap or borehole. Apart from the activity match, open-water contact activities of an individual were not considered in this analysis, as the GPS data did not contain any definitive information on the specific open-water contact activity an individual engaged in. We also used an individual’s mobility tercile as a covariate (see below). At the household level, aside from distance to the closest sites and taps or boreholes, a self-reported variable for whether the household used a safe drinking water source was also used. Taps and boreholes were counted as safe, whereas open freshwater sources such as swamps or lakes were counted as unsafe. To account for geographic, cultural, behavioural and environmental factors that differed across study locations, a district-level categorical variable was included in some models. Households paid different prices for tap usage compared with boreholes (see results); therefore, we also generated a variable indicating whether each public water supply was a tap or a borehole.

#### Schistosome reinfection

To test associations between water contact and (re)infection intensity, we relied on schistosome infection data from the SchistoTrack study. All participants were tested for schistosome infection using Kato–Katz microscopy at baseline, four to five weeks later following treatment with praziquantel and at one-year follow-up. Baseline testing took place before the GPS logger handout and the study team was unaware of the Kato–Katz results. Treatment with praziquantel occurred irrespective of infection status and participants remained unaware of their infection status throughout the study. Infection intensity was measured in EPG of stool. The one-year post-treatment infection measurement was chosen as this is the interval for MDA in endemic areas and it is frequently used as a time frame to assess reinfection^13,55,56^. Among participants, 0.4% (2/452) were missing Kato–Katz results at baseline, 3.1% (14/452) were missing them at treatment follow-up and 20% (91/452) were missing them at one-year follow-up. For those individuals, we imputed the reinfection intensity based on the baseline infection intensity using the multivariate imputations by chained equations technique, implemented in the mice package in R^57^.

### Human mobility measures

We aimed to compare human mobility patterns across different activity types: visits to open-water sites or taps or boreholes and overall movement. To do so, we selected the radius of gyration *R*_g,*i*_ as our metric, as it measures the Euclidean typical displacement of individual *i* from their household location and is widely used in human mobility modelling^58–60^. This choice ensured consistency with our use of Euclidean distances for calculating household distances to visited open-water sites and taps or boreholes. *R*_g,*i*_ was computed using the following formula:

1 $${R}_{{\rm{g}},i}=\sqrt{\frac{1}{N}{\sum }_{l=1}^{N}{({\vec{r}}_{l}-{\vec{r}}_{{\rm{h}}})}^{2}}$$

where ${({\vec{r}}_{l}-{\vec{r}}_{h})}^{2}$ represents the squared Euclidean distance from location *l* to the household location *h*.

### Spatial decay models

We modelled open-water site and tap or borehole usage as a function of Euclidean household distance, motivated by evidence that open-water contact and schistosome infection are waterbody distance dependent^2,17,23^. Our previous work using self-reported open-water contact and closest site assignment suggested that the water contact over household distance was approximately linear^2^. The modelling framework proposed here differs from previous studies in that it aims to estimate the usage of each open-water site and tap or borehole, as opposed to just the closest site. Our spatial decay models take the general form:

2 $$P({\mathrm{response}}_{i,k}=1)={b}_{0}\times f({d}_{i,k})$$

where *P* is the probability that individual *i* uses a specific open-water site and tap or borehole *k*. Here *f*(*d*_*i*,*k*_) represents the spatial decay function with *d* being the household distance to *k*. The binary outcome (response_*i*,*k*_) is modelled using a Bernoulli distribution with an identity link function to represent probabilities directly. All spatial decay models were fitted as Bayesian nonlinear regression models.

The sequential model-building process we used was as follows. First, we determined a suitable spatial decay function *f*(*d*) in equation (2). We tested whether an exponential decay or a power-law decay—two decay functions used commonly in human mobility modelling^58^—or a Hill function (which includes more parameters to control the curvature) best fit the data (Supplementary Table 7). As models were fitted as Bayesian nonlinear models, the best decay function was determined via ELPD—a Bayesian leave-one-out measure of out-of-sample predictive fit^61^ (see below for more details). Second, we tested whether the inclusion of covariates, added one variable (*j* in equation (3)) at a time (from district, gender, age (<15 or ≥15 years), occupation category or activity match), improved the model fit compared with the base model without additional covariates (global model; equation (2)).

3 $$P({\mathrm{response}}_{i,j,k}=1)={b}_{0,j}\times f_{j}({d}_{i,j,k}).$$

We then extended equations (2) and (3) to evaluate the influence of spatial distance, human mobility and usage of safe water infrastructure on human contact with open-water sites, as shown in Fig. 3. For competing opportunities (Fig. 3b), we drew up Stouffer’s intervening opportunity framework^62^ to develop a spatially explicit model estimating the decay separately for the first, second, third, …, *n*th closest site. As <1% (4/452) of individuals visited more than five distinct open-water sites, all sites beyond the fifth nearest were collapsed into a single category. For human mobility, the effects (Fig. 3c) were assessed by estimating decay separately per mobility tercile, represented by *j* in equation (3). Crowding out (Fig. 3d) was assessed using equation (5).

Analogously to the model predicting the probability of open-water-site usage in equation (2), we used the same modelling approach to predict tap or borehole usage, with *P*(response = 1) representing the probability of using a tap or borehole. We also used spatial decay models to predict the duration of open-water site and tap or borehole usage. These models took the following form:

4 $$\,E({t}_{i,k})={b}_{0}\times f({d}_{i,k}).$$

We modelled equation (4) using a zero-inflated negative binomial model:

$${t}_{i,k} \sim \left\{\begin{array}{cc}0, & \,\mathrm{with\; probability}\,{\pi }_{i,k},\\ \,\mathrm{NegBin}\,({\mu }_{i,k},\phi ), & \,\mathrm{with\; probability}\,1-{\pi }_{i,k},\end{array}\right.$$

where *μ*_*i*,*k*_ = *b*_0_ × *f*(*d*_*i*,*k*_) is the mean duration for individual *i* at site *k*, and *ϕ* is the dispersion parameter, which allows the negative binomial to model strong overdispersion and a heavy right tail in visit durations. The zero-inflation probability *π*_*i*,*k*_ accounts for excess zeros beyond those produced by the negative binomial component. Due to the disproportionate influence that a very small number of extreme duration values can have on the dispersion parameter *ϕ*, and to obtain parameter estimates that were more representative of typical open-water contact behaviour, we capped all durations above the 99.9th percentile at that percentile value (>139 min d^−1^ set to 139). This transformation affected only 0.1% of observations and preserved the rank order of almost all data while limiting the undue influence of extreme values on the zero-inflated negative binomial fit. For exponential duration decay models, we used an uninformative prior, Unif(0, 1), for *b*_1_ and a normal prior, Normal(1, 9), truncated at zero and 30, for *b*_0_. This prior was chosen to match the mean and standard deviation of the data.

We also built spatial decay models in which the usage of taps or boreholes reduces the probability and frequency of open-water-site usage (crowding-out models) and compared the predictive performance with that of models without crowding-out effects. For the probability of open-water-site usage with crowding out, the model took the following form:

5 $$\begin{array}{l}P(\mathrm{water}\,\mathrm{site}\,{\mathrm{usage}}_{i,k}=1)\\ ={b}_{0}\times f_{j}({d}_{i,k})\times \left(1-\alpha \times P(\mathrm{tap}\,\mathrm{or}\,\mathrm{borehole}\,{\mathrm{usage}}_{i})\right)\end{array}$$

where the probability of open-water-site usage is inversely related to the probability of tap or borehole usage and where *α* quantifies the magnitude of crowding out. Again, this probability is estimated for each individual *i* and open-water site *k* pair. A model analogous to equation (5) is used for the duration of open-water-site usage.

Supplementary Table 7 provides a summary of all of the relevant spatial decay functions and models presented in this study.

### Estimating spatial decay models

We used Bayesian nonlinear regression models, implemented using the brms package^63^ in R version 4.1.0, to fit spatial decay models. Bayesian modelling was used because it allowed us to estimate distributions of the decay parameters and quantify the uncertainty of these parameter estimates by taking posterior draws from these distributions to obtain 95% CrIs. Due to convergence issues in the Bayesian modelling, we restricted our data to all sites within 3,000 m of the households, which captured over 99% of all open-water sites and taps or boreholes used. For all Bayesian models estimated via brms, a Hamiltonian Monte Carlo sampling algorithm via the Stan backend, employing the No-U-Turn Sampler, was used^63,64^. Four Markov chains with 2,000 iterations per chain (1,000 warm-up iterations and 1,000 sampling iterations) were employed. Convergence was assessed based on whether the potential scale reduction factor ($\widehat{R}$) was close to 1. Within brms, the plot function was used to assess chain mixing visually and the pp_check function was used to assess agreement between predicted and observed response values.

As far as possible, we used non-informative priors. For instance, in the exponential decay model, we used uniform priors Unif(0, 1) for *b*_0_ and *b*_1_, the most conservative priors possible, as *b*_0_ and *b*_1_ can only take values between 0 and 1. Model fitting enforced *P*(response_*i*,*j*,*k*_ = 1) ∈ [0, 1] throughout. Where model outputs were used to assign individuals to open-water sites, predicted probabilities were converted to binary outcomes using the optimal cut-point probability determined by the cutpointr package with the F1 score as the criterion^65^.

### Selecting the best-fitting models

To determine which decay function best fit the data, we used Bayesian leave-one-out cross-validation. The best fit was identified based on ELPD for a new dataset—a Bayesian leave-one-out measure of out-of-sample predictive fit^61^. When comparing a model against a reference model, negative ELPD values signify lower predictive density, indicating that a model performs worse than the reference model. For the decay function, ELPD was compared between global Hill, exponential and power-law decay models (Supplementary Table 7). We additionally report AUROCs and their 95% CIs from fivefold cross-validation to evaluate the ability of the models to correctly discriminate between used and unused open-water sites or taps or boreholes for each individual. When computing AUROCs, stratification based on district was performed when the folds were constructed to ensure proper representation of each district. In models where one district at a time was held out to evaluate predictive performance on data from this district using a model trained on the other two districts (Supplementary Fig. 12), the training data comprised 50 open-water sites per individual, sampled with replacement from the full data to ensure a more balanced dataset across districts when calculating AUROC.

Importantly, we evaluated the predictive performance of the spatial decay models at the individual level, which is a substantially more stringent approach compared with how gravity models (which are akin to our spatial decay models) are conventionally evaluated. As gravity models are not at the individual level, Pearson correlation coefficients or *R*^2^ values between predicted and observed aggregate mobility flows are typically used for evaluation^66–68^. For fine-scale predictions, gravity models have performed poorly, with *R*^2^ values for predicting commuting behaviour in London of between 7–22%^67^. Here, we are not primarily interested in measures of aggregate performance but in the more difficult problem of predicting individual-level usage patterns. This problem is more challenging because we aim to predict not whether any open-water contact occurs but the specific open-water site(s) that individuals visit. Consequently, if an individual visits a different water site than the one predicted by the model, this would not be counted as a classification mistake when the goal is to predict the aggregate number of individuals with open-water contact. In our case, however, this case would introduce two classification mistakes (one open-water site incorrectly predicted as not being visited and another being incorrectly predicted as visited).

### Out-of-sample predictions of open-water contact

The ability of spatial decay models to predict population-wide patterns of open-water contact was demonstrated using the publicly available Global Google–Microsoft Open Buildings Dataset (V3), which has mapped over two billion structures globally^42^. Footprints of all buildings in a 10 km × 10 km area around the study villages in Pakwach were extracted. As there were few commercial or public buildings in this rural area, we assumed that each building represented a household.

To generate population-wide predictions of open-water contact, we calculated the centroid location of each building and input the centroid GPS coordinates into equation (3), using the district-specific decay model for Pakwach. This produced out-of-sample predictions of open-water-site usage for all households in the study villages in Pakwach.

### Sensitivity and robustness analyses

Several sensitivity and robustness analyses were conducted to account for the potential for open-water contact misclassification, systematic differences in compliance across demographic groups, and differential logger failure across districts.

As most open-water contact events are brief (in the range of minutes), there is a risk of misclassification, for instance due to passing by an open-water site without having actual open-water contact. To evaluate the influence of such brief, potentially misclassified contact events, we successively increased the threshold below which we removed events (0–10 min). We then recalculated the spatial decay model predicting open-water-site usage, including only open-water contact events above these thresholds, to understand how the model coefficients *b*_0_ and *b*_1_ changed. This thresholding exercise also informed on the minimum GPS logger sampling frequency needed to reproduce the patterns observed with GPS data at two-minute intervals.

Comparisons were made between the demographic characteristics of individuals with sufficient GPS data (≥2 days), those with insufficient data (<2 days or completely missing data) and the broader SchistoTrack study population. This analysis provided insights into potential selection biases arising from sampling or the distribution of loggers and participant non-compliance. Among those with GPS data, terciles of the number of GPS points per individual per day (calculated within each district to account for deteriorating battery life due to device reuse) were used as an indicator to examine differences in non-compliance with wear between demographic groups.

This study was conducted across three different districts; behaviour patterns from one district may not be generalizable. To address this, data from two districts at a time were used to predict water site usage in the held-out district using the global spatial decay model of open-water contact (equation (2)). The numbers of individuals and observation days were downsampled to determine the minimum numbers of individuals and observation days required to achieve good model performance, which was assessed based on AUROCs. Although participants were required to contribute a minimum of two days of GPS logger data to be included in this analysis, we assessed for included participants whether even a single day of GPS logger data from all participants yielded model performance based on the AUROC.

### Spatially explicit transmission model

We constructed a modular, spatially explicit IBM to simulate the dynamics of reinfection in our study population using Julia (version 1.11.3). We informed our model from existing IBMs, adapting snail dynamics from the SchiSTOP model^21^, incorporating them at the site level rather than aggregated and adapting human infection dynamics from the SCHISTOX model^20^. Parameter values were derived from these two models or based on biological realism, but were not directly fitted to the data. Initial snail states were informed by Iacovidou et al.^26^, with aggregated information for the water site clusters, and initial worm burdens in humans were informed by the EPG data of the 452 individuals who participated in the GPS logger study.

Spatial explicitness in the model was introduced using weighted bipartite networks (one for each district), where nodes represent households and open-water sites and edges were weighted according to the probability, *P*, of site usage, as calculated using the spatial decay function (equation (2)).

We considered four decision rules regarding site usage, where each rule represents an independent visitation decision per individual per time step:

- Visit the nearest open-water site with 100% probability.
- Visit the nearest open-water site with probability *P*.
- Each site is given a success (1) or failure (0) score based on Bernoulli trials with probability *P*. Individuals visit a single site with a success score chosen at random. If no successes are recorded, individuals stay home.
- Same as the previous rule, but individuals visit all sites that return a success score from the Bernoulli trials.

The first three rules only allow individuals to visit a maximum of one site per day, with the first two rules restricting visitation to their nearest site. All rules, except the first one, are informed by the spatial decay function.

We simulated 100 replicates for each decision rule for 365 days, considering the study population of 452 individuals. The aims were to investigate whether information from the spatial decay models was informative for predicting infection prevalence one year after treatment and to assess the site-specific visitation distributions from the different rules. Site visits were calculated by counting, for each replicate, how many times individuals visited a given site, then summarizing these counts across all replicates to obtain average visit frequencies per site. By taking the cumulative miracidial input at the site level, we computed the relative contamination for each site as a percentage of the most contaminated site within each district.

We performed sensitivity analysis on the aggregation parameter (as was also done in Graham et al.^20^ when using the SCHISTOX model) by showing the predicted prevalence results using lower values. However, we believe a higher value is more consistent with the observed prevalence in our study area.

### Inclusion

This study was co-designed and implemented by researchers based in Uganda and the United Kingdom. Ugandan investigators from the Division of Vector-Borne and Neglected Tropical Diseases at the Ministry of Health and local partners contributed to the study design, community engagement, field implementation, data curation and manuscript preparation, and all listed authors meet journal authorship criteria, with additional contributors acknowledged. Characterizing fine-scale open-water contact behaviours and identifying key transmission sites emerged as a local priority through local community engagement activities as part of SchistoTrack, and the research results are shared with communities during annual sessions. Research ethics approval was obtained from the Oxford Tropical Research Ethics Committee, Vector Control Division Research Ethics Committee of the Uganda Ministry of Health and Uganda National Council for Science and Technology. All participants received remuneration (10,000 UGX, equivalent to ~US$2.73 or one day of wages) upon enrolment into SchistoTrack and an equal amount upon completion of the GPS logger sub-study. Those with confirmed *S. mansoni* infection received praziquantel in line with Ugandan national guidelines. No biological specimens, cultural artefacts or associated traditional knowledge were exported outside the country. Training and capacity-building workshops for household survey data collection, environmental sampling and spatial mapping using geographical information software were conducted as part of SchistoTrack.

### Reporting summary

Further information on research design is available in the Nature Portfolio Reporting Summary linked to this article.

## Supplementary information

Supplementary Information Supplementary text, Figs. 1–19 and Tables 1–7.

Reporting Summary

Peer Review File

## Data Availability

Due to the highly sensitive nature of the GPS logger data, restrictions from the data protection impact assessment and the ongoing nature of the SchistoTrack cohort, these data are not publicly available. Researchers wishing to access the data may submit a written request to the corresponding author. We will consider requests from researchers with a legitimate scientific purpose, subject to the execution of a data sharing agreement with the University of Oxford that specifies permitted uses and any applicable data protection conditions. Extensive metadata have been provided in the manuscript and supplementary materials.

## Code availability

The computer code used to produce all of the figures and tables in the main manuscript and supplementary materials is available on FigShare at 10.25446/oxford.31573027 (ref. ^69^).
