# Supplementary material for: Predicting schistosome transmission in rural Uganda using water contact data from wearable GPS devices
Source: Nat Health. 2026 May 22;1(8):850–64. doi: 10.1038/s44360-026-00118-w (PMC13433293; doi:10.1038/s44360-026-00118-w)
Supplement: Supplementary file 1 [file 44360_2026_118_MOESM1_ESM.pdf]

nature health

Supplementary information https://doi.org/10.1038/s44360-026-00118-w

Predicting schistosome transmission in rural Uganda using water contact data from wearable GPS devices

In the format provided by the authors and unedited

Contents

S1 Supplementary text 3

S1.1 - Association of self-reported and GPS-derived open water contact with one-year schistosome infection outcomes . . . 3

S1.2 - Association of tap/borehole usage with (re)infection . . . 3

S1.3 - Association of human mobility with open water contact and usage . . . 3

S1.4 - Comparison of self-reported and GPS-derived open water contact patterns . 4

S1.5 - Predicted number of site visits from the individual-based model . . . 4

S1.6 - Reductions in open water contact due to safe water infrastructure . . . 5

S1.7 - Minimum recording frequency and days of GPS logger data . . . 6

S1.8 - GPS data missingness and logger wear non-compliance . . . 6

S1.9 - Applying spatial decay models to predict population-wide open water contact 7

S1.10 - Biases in spatial decay model predictions by demographic characteristics . . 7

S2 Supplementary figures 9

Fig. S1 - Diurnal patterns of open water site and tap/borehole usage . . . 9

Fig. S2 - Infection intensities pre-treatment, post-treatment, and at one-year follow-up 10

Fig. S3 - Open water site and tap/borehole usage over age . . . 11

Fig. S4 - Comparison between different spatial decay functions . . . 12

Fig. S5 - Comparison between exponential spatial decay and logistic regression models 13

Fig. S6 - Assessing spatial decay model performance . . . 14

Fig. S7 - Spatial decay models of open water site usage incorporating district-specific intercepts and decay functions . . . 14

Fig. S8 - Applying spatial decay models to predict population-level water site usage . 15

Fig. S9 - Open water site usage by sequence of sites . . . 16

Fig. S10 - The influence of taps/boreholes on open water site usage probability . . . 17

Fig. S11 - The influence of taps/boreholes on open water site usage duration . . . 18

Fig. S12 - Minimum data needs for estimating the spatial decay models . . . 19

Fig. S13 - The impact of removing short-duration open water site and tap/borehole usage events . . . 19

Fig. S14 - Robustness to buffer size choice . . . 20

Fig. S15 - Relative miracidial contamination by site, district, and visitation rule . . . 21

Fig. S16 - Sensitivity analysis for infection dynamics . . . 22

Fig. S17 - GPS logger in waterproof pouch and with attached lanyard . . . 23

Fig. S18 - Participant flowchart 24
Fig. S19 - Crowding out of open water site usage duration over household distance . . 25

S3 Supplementary tables **26**

Table S1 - Summary of key participant characteristics. 26
Table S2 - Logistic regression models predicting open water site usage. 27
Table S3 - Model selection for spatial decay models. 27
Table S4 - Comparison of participants with sufficient GPS data versus those with missing data and the wider SchistoTrack study population. 28
Table S5 - Comparison of participants by number of recorded GPS points, grouped into terciles. 28
Table S6 - Differences in spatial decay model predictive performance across sociodemographic factors. 29
Table S7 - Overview of spatial decay models presented in this paper. 30

S1 Supplementary text

S1.1 Association of self-reported and GPS-derived open water contact with one-year schistosome infection outcomes

We compared relationships between self-reported and GPS-derived open water contact and one-year infection following treatment of all individuals after baseline. Based on GPS data, median one-year infection intensity among infected individuals with any open water contact was 48 (IQR 24 to 156) EPG compared to 36 (IQR 27 to 93) EPG for people without open water contact during the observation period (Wilcoxon rank-sum test, $p = 0.42$). Based on self-reported behaviour, median one-year reinfection intensity among infected individuals with open water contact was 48 (IQR 24 to 120) EPG compared to 48 (IQR 24 to 123) EPG for people without any self-reported open water contact ($p = 0.78$). We also found that among people who were classified as having no open water contact based on GPS data and were not found to be infected after treatment, 24.6% (35/142) became reinfected after one year. Similarly, among people who were classified as having no open water contact based on self-reports, 37.0% (67/181) became reinfected after one year.

S1.2 Association of tap/borehole usage with (re)infection

We assessed relationships between GPS-derived tap/borehole usage and one-year reinfection patterns. Note, no measure of self-reported individual-level tap/borehole usage was collected in SchistoTrack. Based on GPS data, median one-year reinfection intensity among infected individuals with tap/borehole usage was 48 (IQR 24 to 111) EPG compared to 48 (IQR 24 to 132) EPG for people without any tap/borehole usage during the observation period ($p = 0.90$).

S1.3 Association of human mobility with open water contact and usage

There was high variability of movement within districts; participants in Pakwach had the greatest area of movement with a median radius of gyration of 607 m (IQR 228 to 1407 m, $n = 183$), followed by individuals in Mayuge (median 281 m, IQR 136 to 682 m, $n = 105$) and Buliisa (median 253 m, IQR 89 to 1082 m, $n = 164$). Fishermen (median 658 m, IQR 187 to 1757 m, $n = 41$) were not substantially more mobile than other adults (median 506 m, IQR 134 to 1478 m, $n = 186$). Individuals who lived >1000 m from water sites had greater median radii of gyration (median 621 m, IQR 311 to 1260 m, $n = 12$) than those living within 0 to 500 m (median 349 m, IQR 143 to 1083 m, $n = 440$, Fig. 2d), though not statistically significant due to the high level of individual variation.

S1.4 Comparison of self-reported and GPS-derived open water contact patterns

Here, we compare GPS-derived open water contact with self-reported open water contact from the household survey. Among study participants, 63.9% (289/452) used one or more open water sites, based on the GPS data over the 10-day observation period. In the survey data, 51.1% (231/452) among those same participants reported they had any contact with open waterbodies in the past week. The correlation between those two measures of open water contact was $\rho_s = 0.10$ ($p = 0.03$). The median duration of open water site usage from GPS among participants was 2 min per day (IQR: 0 to 12.3 min), whereas the median duration of self-reported open water contact was 2.1 min (IQR: 0 to 25.7 min). The correlation between these two measures of open water contact duration was $\rho_s = -0.17$ ($p < 0.01$). In terms of age-dependent patterns of open water contact, we found that the trend shown for the GPS data in Fig. S3 was similar to the age-dependent trends of self-reported open water contact from SchistoTrack reported in Reitzug et al. [1]. We note that GPS-derived and self-reported open water contact are not expected to be in perfect agreement, as they were collected one week apart, self-reports were elicited from the household head for all household members and GPS-derived open water site usage focused on contact with specific shallow open water sites. In contrast, self-reported open water contact also included contact with deep water away from the shoreline.

S1.5 Predicted number of site visits from the individual-based model

The distribution of the predicted number of contacts for each site throughout a one-year simulation period (standard period for reinfection) is presented in Extended Data Fig. 1. This section provides more detailed statistics of the results presented in Extended Data Fig. 1. In Pakwach, 100% of visited sites (7/7 based on rules (1) and (2) and 9/9 based on rules (3) and (4)) had snail presence. Using rule (1), a median of 9490 visits (IQR [6205, 13505]) were predicted per site, compared to 1859 (IQR [1238, 3411]) using rule (2), 3993 (IQR [3480, 4425]) using rule (3), and 6364 (IQR [4776, 6593]) using rule (4). In Buliisa, 63.6% of visited sites (7/11) based on rules (1) and (2) had snail presence and 69.2% (9/13) based on rules (3) and (4). The median number of predicted visits per site was 5110 (IQR [3650, 7665]) using rule (1), 2678 (IQR [2099, 4321]) using rule (2), 4967 (IQR [2569, 6376]) using rule (3), and 11527 (IQR [5715, 14826]) using rule (4). Lastly, in Mayuge, 80% of visited sites (4/5) had snail presence based on rules (1) and (2), whereas 87.5% (7/8) had snail presence based on rules (3) and (4). Simulations using

rule (1) predicted a median of 5475 (IQR [5110, 11315]) visits per site, 1862 (IQR [1140, 2818]) using rule (2), 2110 (IQR [596, 2397]) using rule (3), and 2521 (IQR [763, 2773]) using rule (4).

S1.6 Reductions in open water contact due to safe water infrastructure

This section provides more details on the analysis outlined in the corresponding section of the main text. Although model selection using expected log pointwise predictive density (ELPD) indicated that crowding out models did not outperform standard models in predicting open water site usage or duration, we present these findings to quantify a counterfactual scenario. Specifically, we explore the implications of a scenario where WASH interventions successfully reduce open water contact. Within such models the crowding-out parameter α had a value of 0.33 (95% CrI: 0.06 to 0.57), suggesting that taps/boreholes might reduce the probability of open water site usage by up to 33%. The crowding-out model estimated that tap/borehole availability diverted open water contact for <2% of individuals (median = 8/452, 95% CrI: 0 to 24 individuals) based on comparing predicted open water contact under observed tap/borehole usage with a counterfactual scenario without any taps/boreholes. The 33% crowding out ultimately translated into a much more modest effect because the 33% reduction was only achieved for individuals with open water contact who also lived close to taps/boreholes (20 m). Reductions in open water contact were unevenly distributed across the population. Individuals with the highest predicted reductions in open water site usage probabilities tended to have the highest tap/borehole usage probabilities, which resulted in the largest crowding-out effects (Fig. S10d).

We repeated the crowding-out analysis for open water site usage duration (Fig. S11). We found again that the crowding-out model did not perform better compared to a model without crowding out (ELPD difference = −0.02, p = 0.99). For duration, the model suggested that existing taps/boreholes had a crowding-out effect of 40% (95% CrI: 0.03 to 0.82), which was larger than the effect on the probability of any open water contact (33%). The average duration reduction was 0.77 min (duration of 4.08 min [95% CrI: 0.90 to 6.57] without taps/boreholes and 3.31 min [95% CrI: 0.78 to 3.68] with taps/boreholes). Almost all reductions (>98%) were concentrated among the individuals in the top three deciles of duration of open water site usage (Fig. S11b). Even under universal tap/borehole usage and crowding out, the upper limit of reductions in open water site usage duration was 23% (95% CrI: 19 to 37%) (Fig. S11c). As people with the highest durations of open water contact tended to have high levels of tap/borehole usage (Fig. S11c), the potential to further crowd out their open water contact with safe water supplies was limited and concentrated in proximity to open water sites (Fig. S19).

In addition to the global crowding out coefficient α, we used equation (5) to also estimate the probability of crowding out α_{Ac} separately for different types of open water contact activities Ac, namely domestic, occupational, and recreational activities. To this end, we restricted the data to individuals who self-reported engaging in these specific activities and estimated the activity-specific crowding out parameter α_{Ac}, which we compared to the global α to investigate whether some types of activities are more amenable to crowding out than others. The results suggested that estimated crowding α_{Ac} among people engaging in domestic open water contact (n=171) was 39% (95% CI: 4 to 72%). For individuals engaging in occupational open water contact ($n = 98$), the estimated crowding was 35% (95% CI: 2 to 72%). For individuals who reported engaging in recreational open water contact ($n = 4$), the estimated crowding was 35% (95% CI: 1 to 91%). Thus, compared to the overall α of 33%, these results did not provide evidence that any particular type of open water contact activity was substantially more amenable to crowding out than overall open water contact.

S1.7 Minimum recording frequency and days of GPS logger data

We assessed the impact of GPS logger recording frequency (2 min) on the parameter estimates in the global spatial decay model predicting open water site usage. To this end, we successively removed all open water site usage events of less than the cut-off c for $c \in \{0, \ldots, 10\}$. By estimating b_0 and b_1 for the restricted dataset based on c, we obtained the minimum sampling frequency needed to obtain parameters within the 95% CrIs we observed with the full data. The results are shown in Fig. S13. Among study participants, who all had a minimum of two days of GPS logger data, though the additional number of days varied, we assessed how many days of data were required for optimal model performance. Using the district-specific spatial decay model, we show that even when selecting just one randomly selected day of GPS logger data from all participants, performance already plateaued based on auROC (Fig. S12b).

S1.8 GPS data missingness and logger wear non-compliance

Sufficient data from GPS loggers were retrieved for 452/584 participants who were offered wearable GPS loggers. To investigate differences between individuals with and without missing data, their demographic characteristics were compared in Table S4. Additionally, comparisons were made between individuals selected for the GPS logger study ($n = 584$) and SchistoTrack participants from the same villages who were not chosen for the GPS logger study ($n = 379$). There were differences regarding district and gender, as gender composition and missingness varied

by district. No significant differences were observed between included and non-included participants regarding age, self-reported open water contact, occupation, or distance to the nearest open water site (Table S4).

As the mean number of GPS observation days varied by district (Pakwach = 13, Buliisa = 12, Mayuge = 11), wear non-compliance was investigated using district-specific terciles of the number of GPS points per person per day (Table S5). This allowed us to understand whether relative levels of compliance varied based on sociodemographic characteristics. We found no significant differences across any of the included variables, except for age. The proportion of those aged ≥ 15 years was higher (64.7%) in the tercile with the highest number of points compared to the bottom tercile (51.0%, $p = 0.04$), indicating that adults may have been more compliant. There was no evidence that open water contact led to higher rates of non-compliance as those with self-reported open water contact were somewhat more well-represented in the top tercile in terms of GPS logger points (58.0%) than the bottom tercile (43.7%, $p = 0.04$; Table S5).

S1.9 Applying spatial decay models to predict population-wide open water contact

Given that our model was highly predictive of the true number of individuals visiting each open water site ($\rho = 0.75$, Fig. S10a,b), we applied this model for out-of-study predictions, focusing on Pakwach by extracting footprints of all 5177 local structures in the catchment around the study villages under the assumption that each building represented one household (see Methods). The results in Fig. S8 suggested that there were substantial differences in open water site usage, even across nearby sites, with one site predicted to be used by 256 households and another by 657 households.

S1.10 Biases in spatial decay model predictions by demographic characteristics

We found that the district-specific spatial decay model outperformed all other decay models in Table S3, including those with different decays based on sociodemographic, behavioural, and WASH factors. Nonetheless, we wanted to understand if there were any systematic biases in the ability of spatial decay models to predict open water site usage across demographic groups. To this end, we compared differences between individuals $<25^{\text{th}}$ percentile of predictive accuracy, 25th–75th, and $\geq 75^{\text{th}}$ percentile (Table S6). Based on this comparison, there were no significant differences across gender ($p = 0.19$), with some suggestion of differences in performance based on age ($p = 0.04$). We also found that fishermen were somewhat more represented in the lower

terciles of predictive accuracy compared to the other groups (14.8% in the $<25^{\text{th}}$ percentile versus 10.7% in the 25th–75th percentile and 4.9% in the $\geq 75^{\text{th}}$ percentile, $p < 0.01$). The results in Table S6 also reveal that predictions were less accurate for people in households using improved drinking water sources ($p < 0.01$). There were no differences in predictive accuracy across mobility levels. Distance also influenced predictive accuracy as households with people in the lowest quartile were a median of 102.8 m and 193.2 m away from open water sites and taps/boreholes, respectively, while these same statistics were 335.2 m and 398.8 m for people in the top quartile (both $p < 0.01$).

S2 Supplementary figures

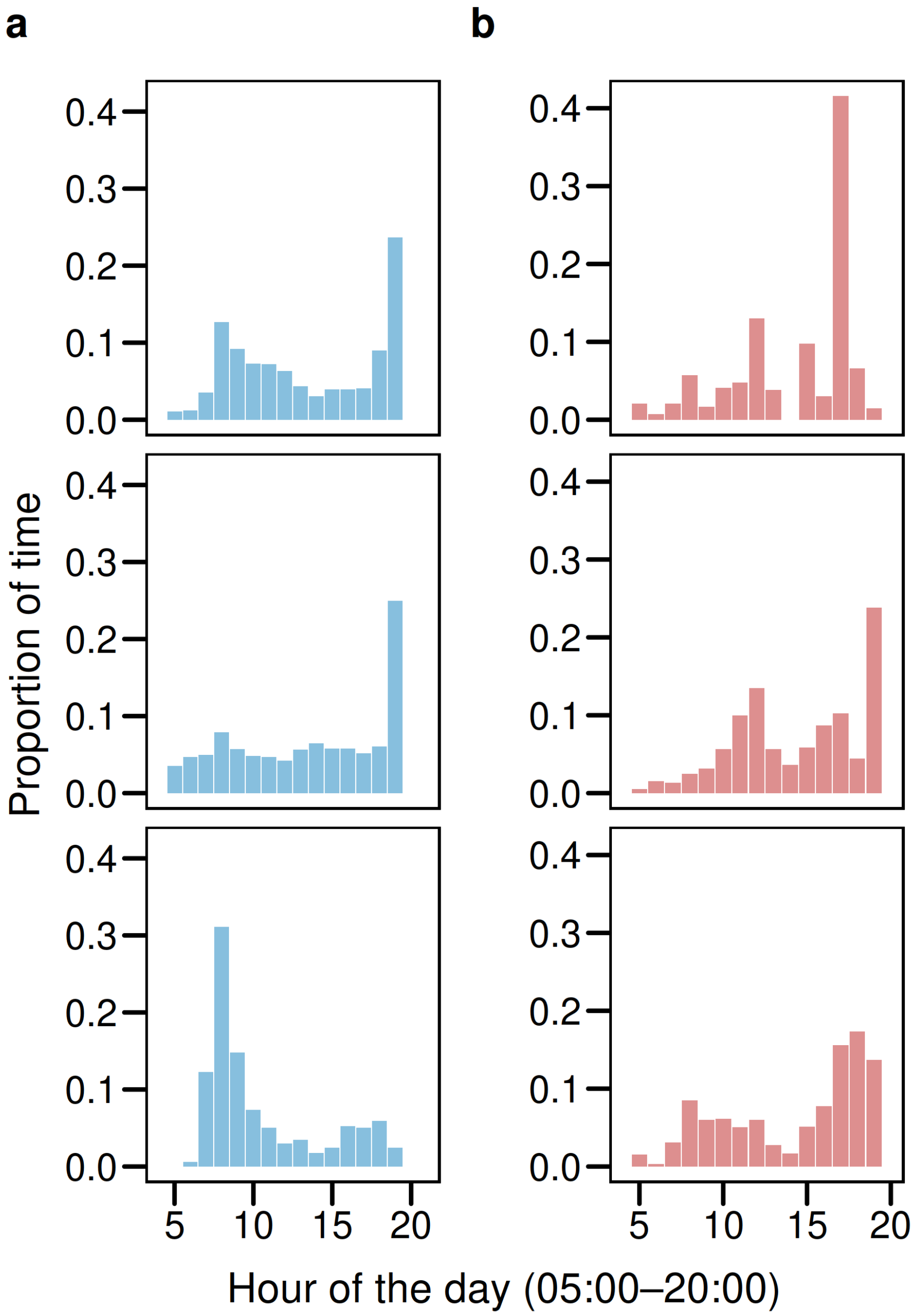

Fig. S1: **Diurnal patterns of open water site and tap/borehole usage. a** Open water site usage by hour of the day. **b** Tap/borehole usage by hour of the day. Both panels are shown for GPS logger observation hours (5:00 a.m.–8:00 p.m.), with each row representing the districts of Mayuge, Buliisa, and Pakwach, respectively.

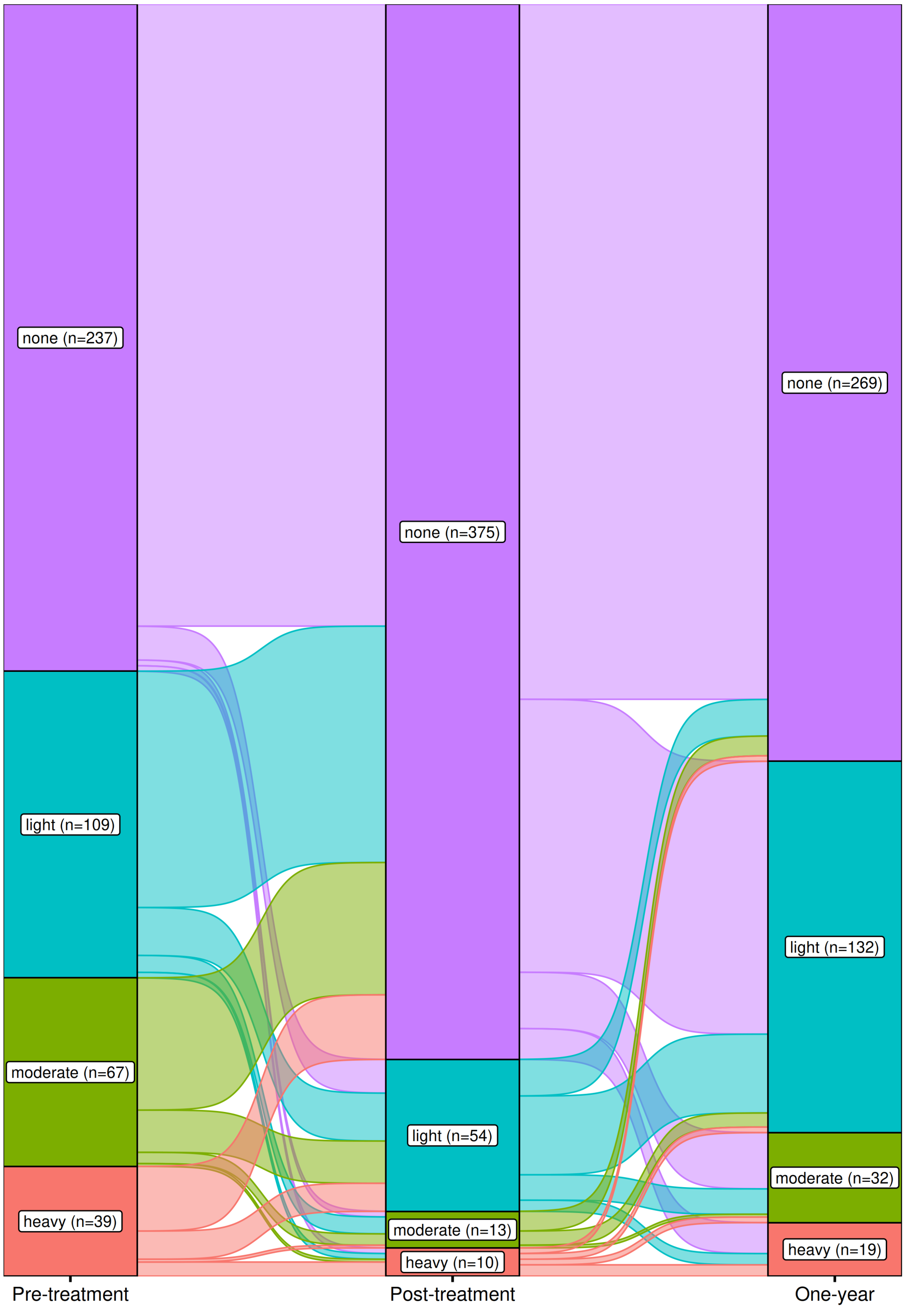

Fig. S2: **Infection intensities pre-treatment, post-treatment, and at one-year follow-up.** Infection intensities as defined by the WHO [2]: none = 0 EPG, light = 1 to 99 EPG, moderate 100 to 399 EPG, and heavy ≥400 EPG.

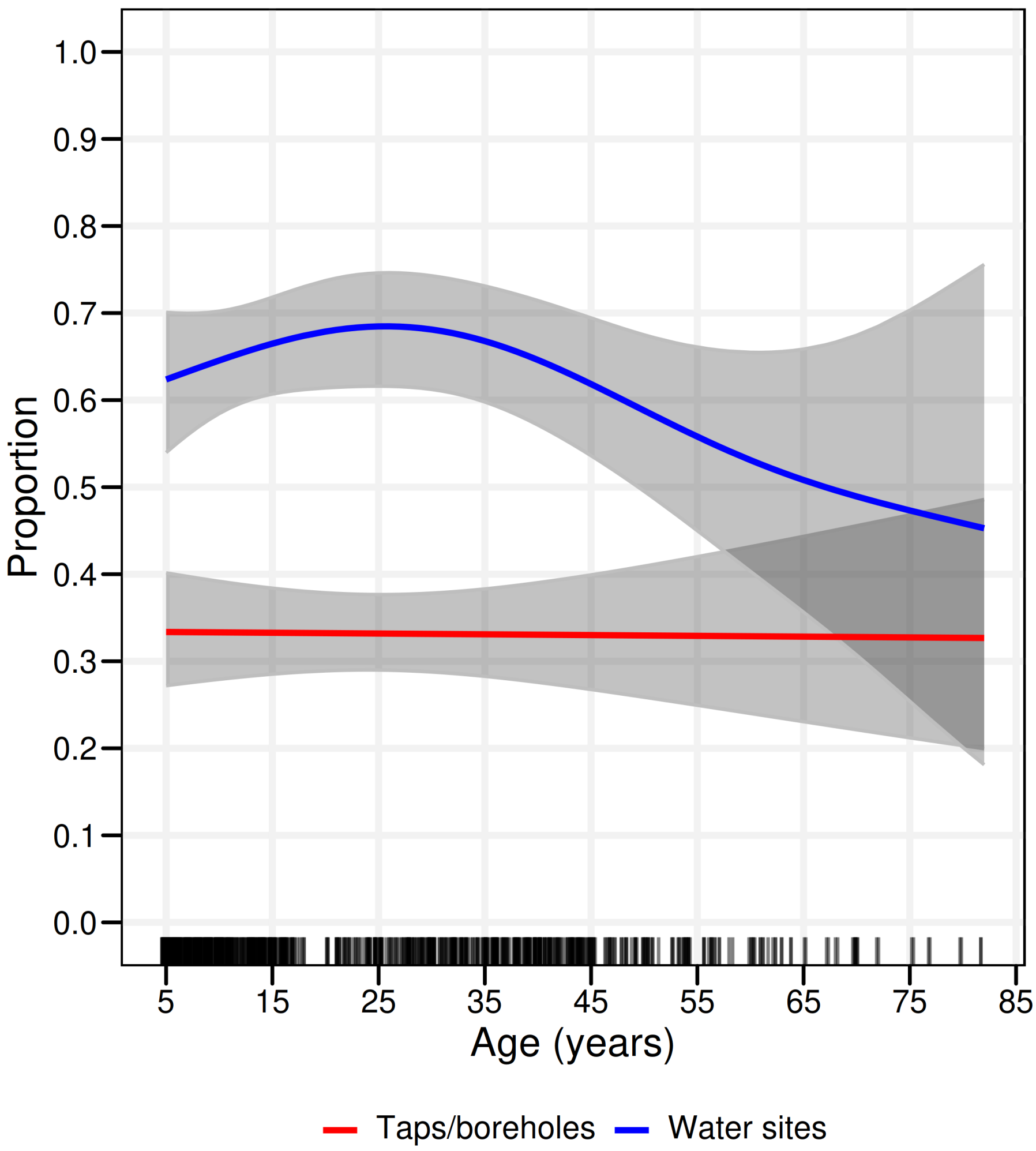

Fig. S3: **Open water site and tap/borehole usage over age.** Generalised additive models showing the proportion of individuals using open water sites and taps/boreholes over age. Shaded grey areas represent 95% confidence intervals.

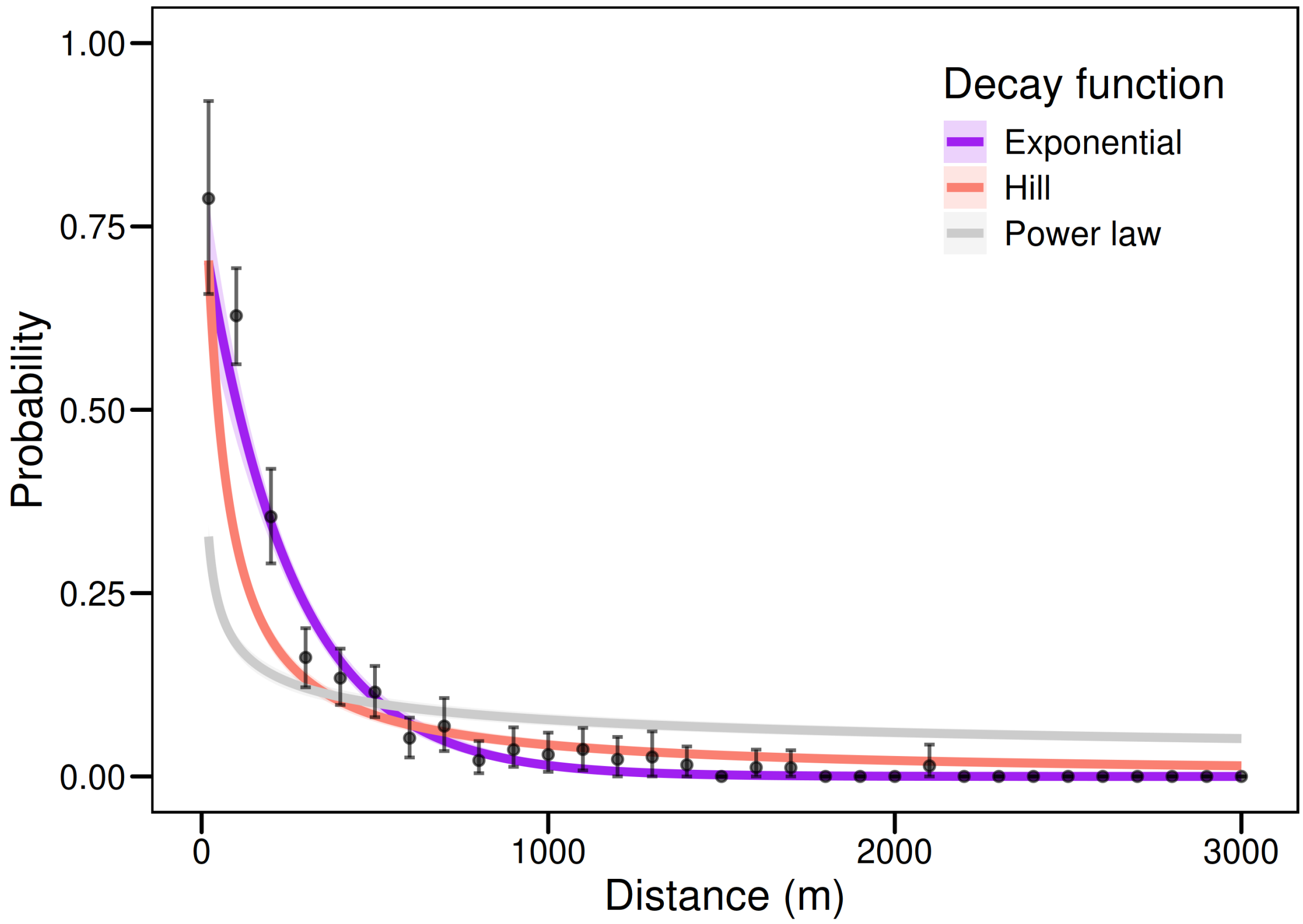

Fig. S4: **Comparison between different spatial decay functions.** Estimated spatial decays from Bayesian models using a power law decay, an exponential decay, and a Hill function. A model comparison based on expected log pointwise predictive density difference suggested that the exponential decay outperformed both the power and Hill functions ($p < 0.01$). Error bars represent the 2.5th and 97.5th percentile ranges calculated using 2000 replicates and plotted in 100 m increments. Points represent the respective means. Shaded grey areas represent 95% credible intervals.

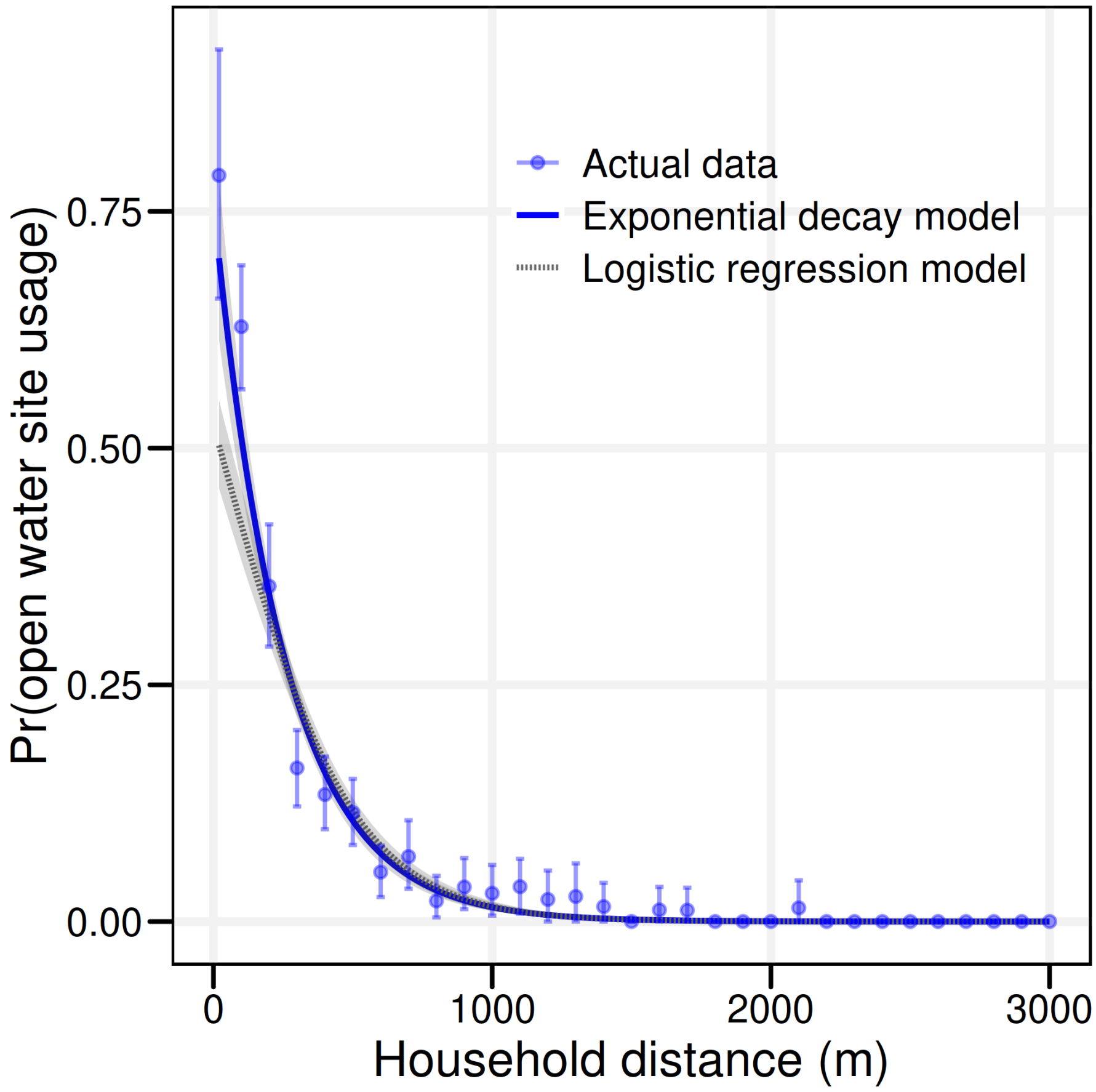

Fig. S5: **Comparison between exponential spatial decay and logistic regression models.** Lines and shaded areas represent predicted values and 95% credible intervals of predicted open water site usage probability from exponential spatial decay and logistic regression models, respectively. Blue error bars represent the 2.5th and 97.5th percentile ranges calculated using 2000 replicates and plotted in 100 m increments. Blue points represent the respective means. The exponential spatial decay model outperformed the logistic regression models based on expected log pointwise predictive density difference (ELPD difference = 152.6, $p < 0.01$). Shaded grey areas represent 95% credible intervals.

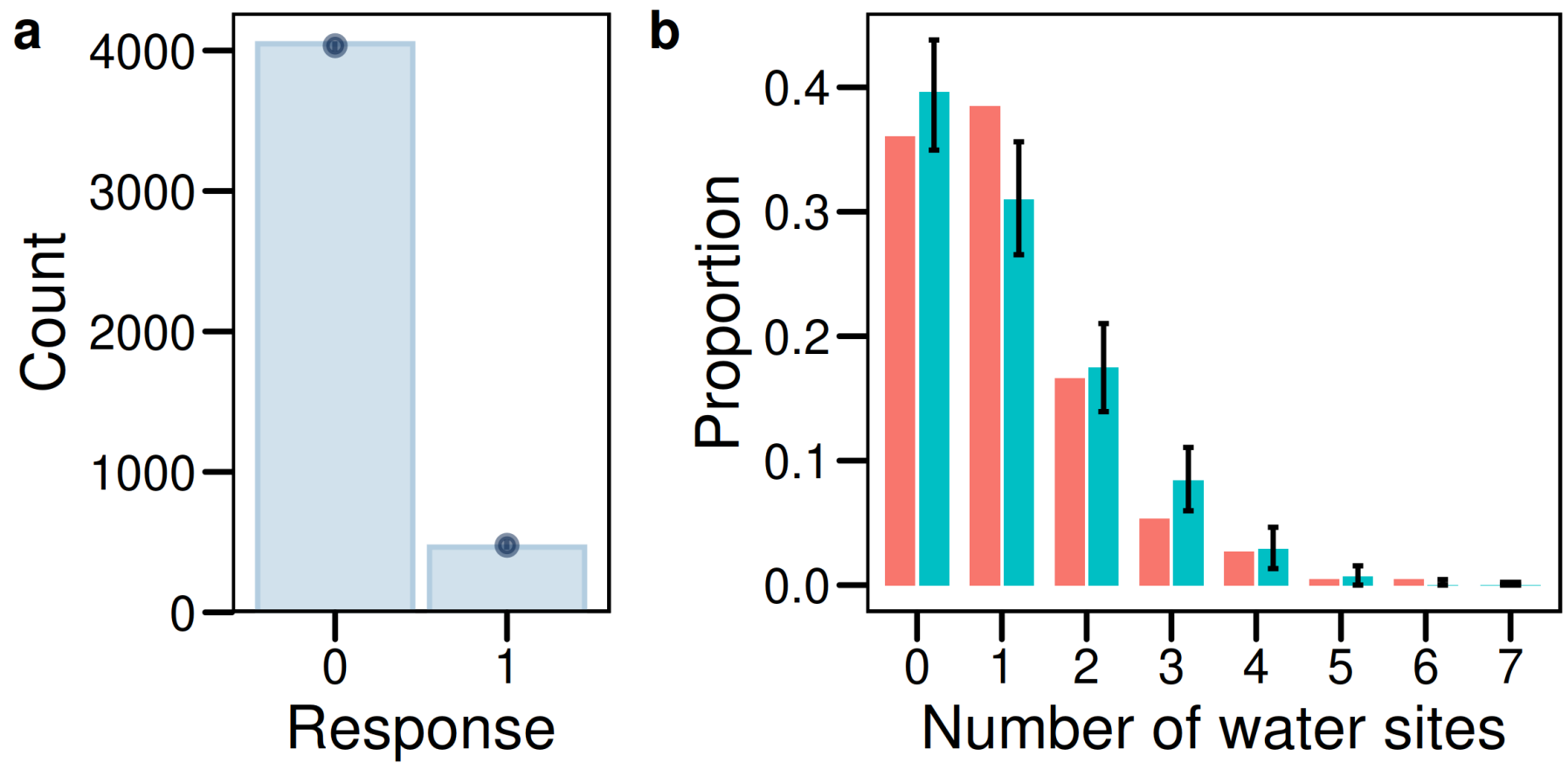

Fig. S6: **Assessing spatial decay model performance. a** True (blue bars) versus predicted number (black dots) of used versus unused open water sites. Response = 0 denotes open water sites not used, and response = 1 denotes used open water sites. Predictions are at the individual level, that is, usage is predicted separately for each individual-site pair. The heights of the bars indicate counts of the total number of individual-site pairs correctly and incorrectly classified. **b** True (red bars) versus predicted number (green bars) of open water sites used per individual, including a credible interval for predicted values (error bars) obtained by taking posterior draws of the predicted proportions in each bin.

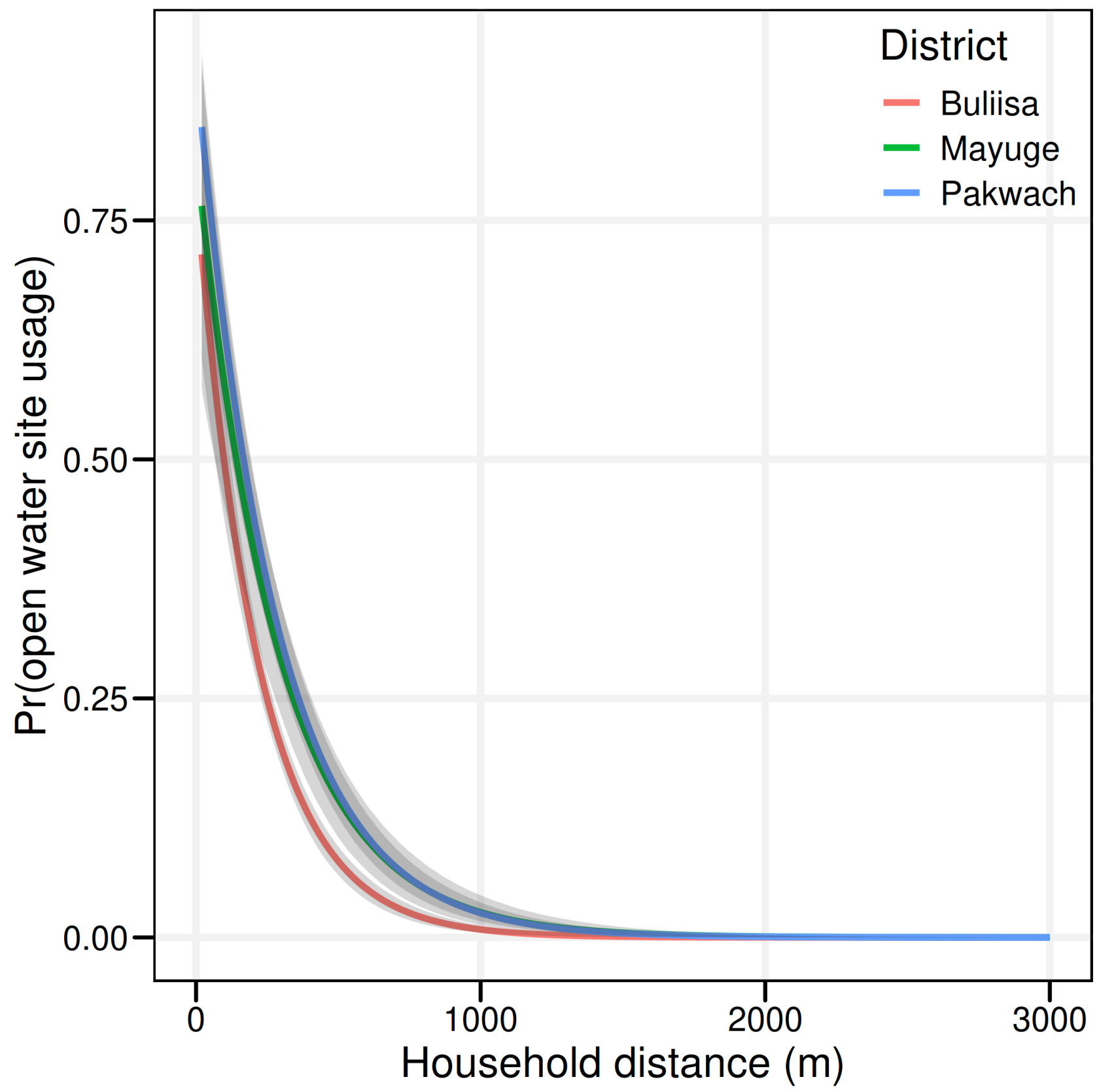

Fig. S7: **Spatial decay models of open water site usage incorporating district-specific intercepts and decay functions.** Shaded grey areas represent 95% credible intervals.

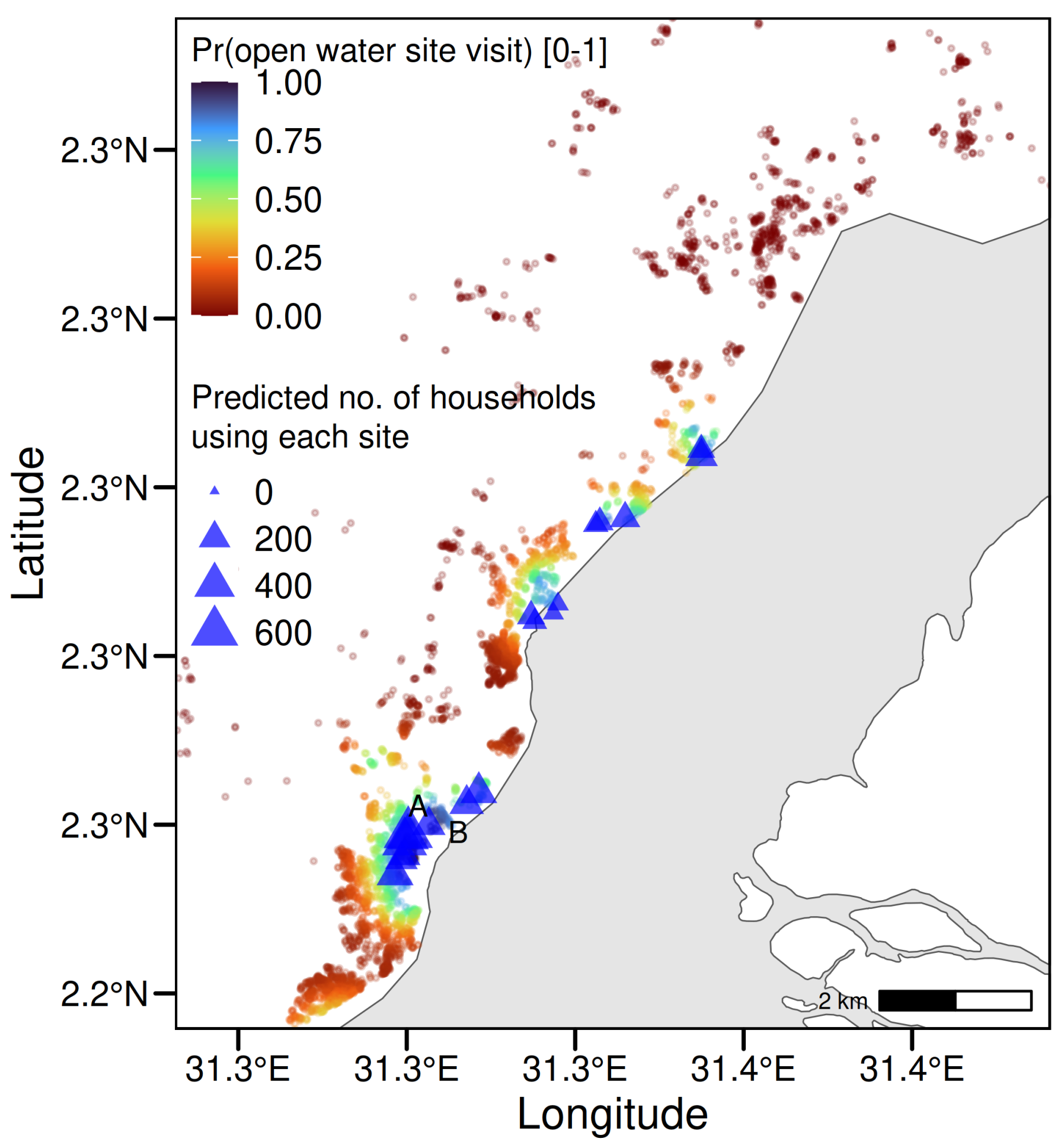

Fig. S8: **Applying spatial decay models to predict population-level water site usage.** Population-wide predictions of the open water site usage probability for each household based on 5177 buildings in Pakwach (each dot represents one building). Colours of the dots represent the predicted probability of using one or more open water sites. Blue triangles show all local water sites mapped by the SchistoTrack study, and their respective sizes indicate the number of households predicted to use each site (multiple assignments possible). Labels 'A' and 'B' denote two nearby open water sites predicted to have substantially different numbers of households using each (657 and 256, respectively).

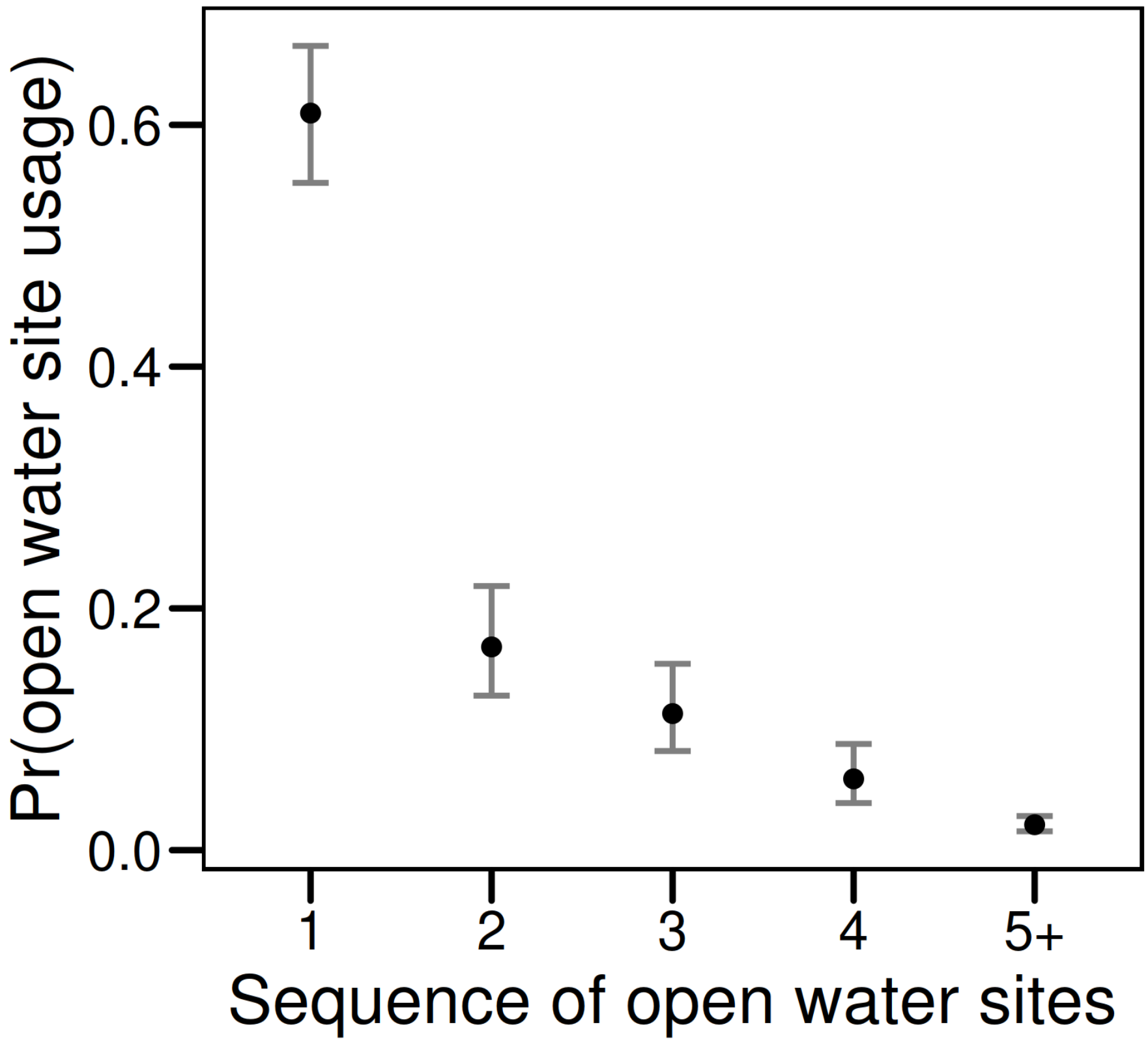

Fig. S9: **Open water site usage by sequence of sites.** Logistic regression model predicting open water site usage probability of an individual by the sequence/rank of open water sites, based on whether a given site was the closest, second closest, . . . , fifth closest/further away site from the household location. Black dots represent point estimates, error bars represent 95% credible intervals.

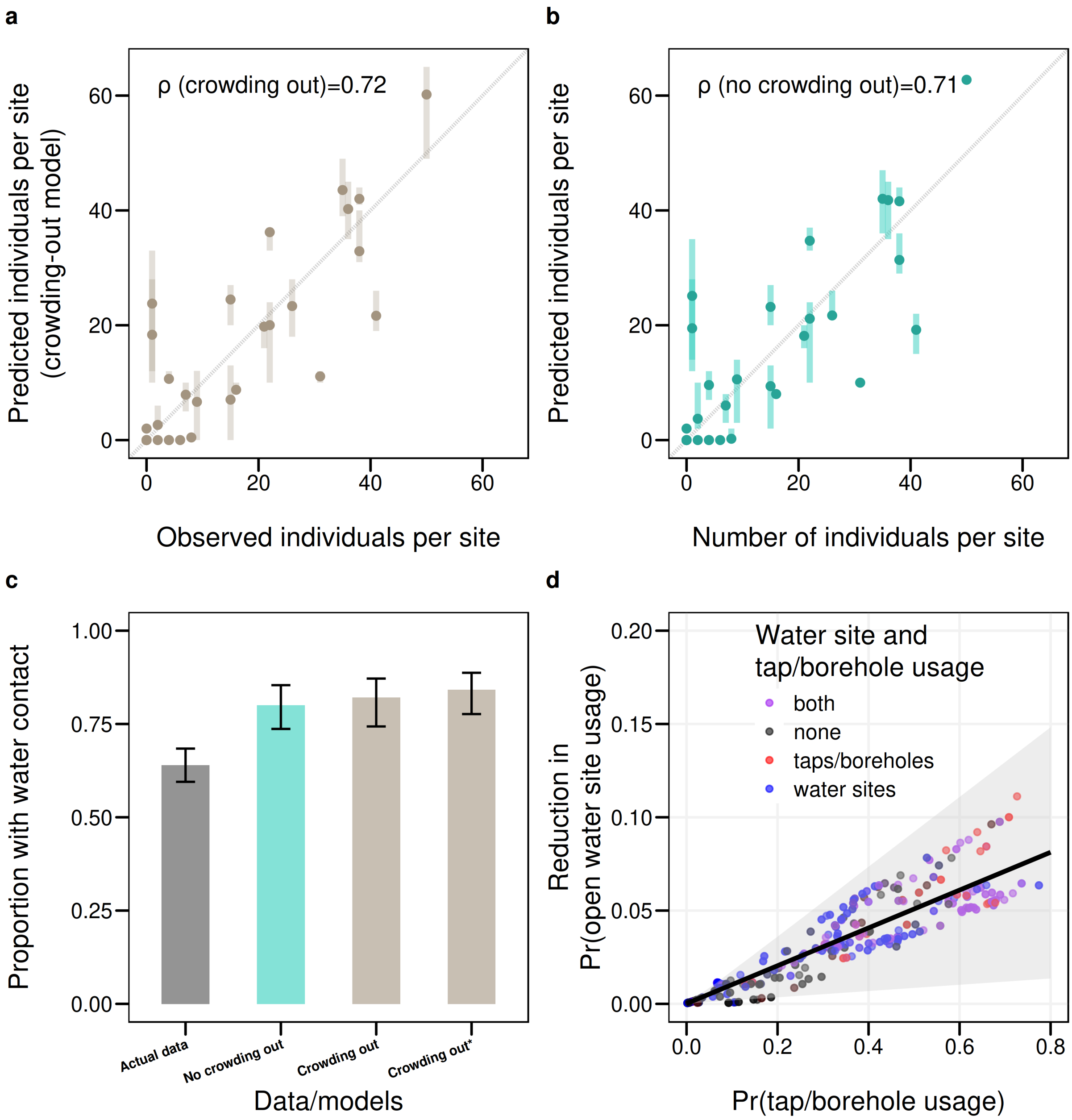

Fig. S10: **The influence of taps/boreholes on open water site usage probability. a,b** Predicted number of individuals using each open water site versus the true number. **c** Observed proportion of individuals with open water contact versus the proportion predicted in spatial decay models with and without crowding out. The 'crowding out*' model corresponds to a scenario in which the proportion of individuals was estimated under a counterfactual scenario where no taps/boreholes were present. Thus, the difference between the 'crowding out' versus 'crowding out*' models represents the estimated impact of taps/boreholes on open water site usage. **d** Scatter plot showing the predicted probability of tap/borehole usage versus the predicted probability of open water site usage for each individual. Colours represent true usage of open water sites and taps/boreholes for each individual. Again, this was estimated by comparing the observed values for tap/borehole coverage to a counterfactual scenario where no taps/boreholes were present. The black line and shaded band represent the posterior mean and 95% credible interval of the regression relationship. Vertical bars in **(a–c)** represent 95% confidence intervals.

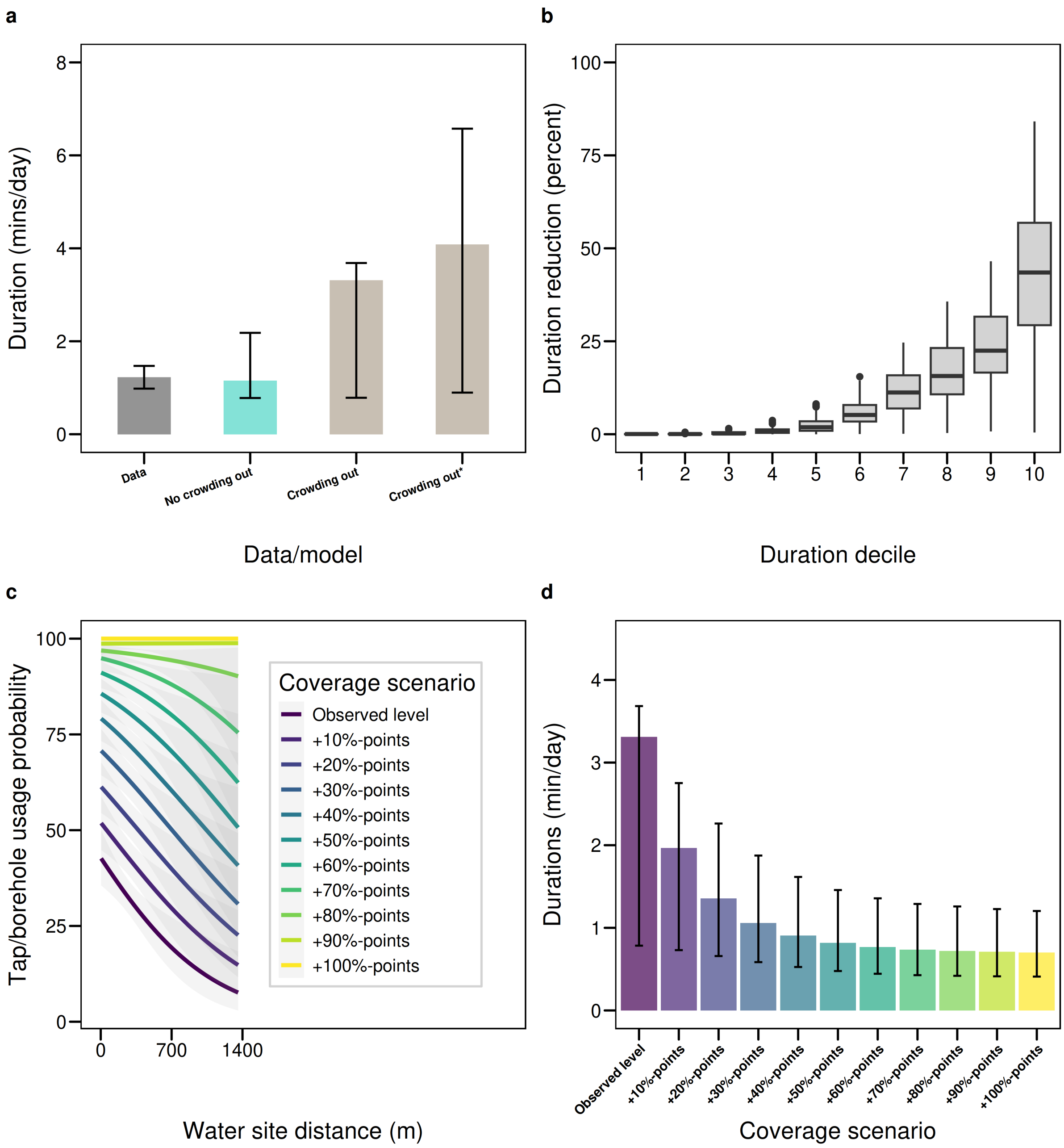

Fig. S11: **The influence of taps/boreholes on open water site usage duration. a** True duration of open water contact per site in min per day versus estimated durations predicted using the crowding-out and no crowding-out models. The 'crowding-out* model' represents a model in which open water site usage duration was estimated under a counterfactual scenario where no taps/boreholes were present. Error bars represent 95% credible intervals. **b** Relative reductions in open water site usage duration by duration decile. The central horizontal line indicates the median, and the box limits represent the interquartile range (IQR). The whiskers extend to the most extreme data points within $1.5 \times$ IQR of the box. Outliers are plotted as individual points. **c** Tap/borehole usage probability estimated from spatial decay model ('observed level', darkest line) and ten different hypothetical scenarios where the tap/borehole usage of individuals was increased by 10–100%-points from the observed level (with 100%-points representing universal usage of taps/boreholes). Shaded areas represent 95% credible intervals. **d** Estimated relative reductions in open water site usage duration under the scenarios shown in **(c)**. Vertical bars in **(d)** represent 95% credible intervals.

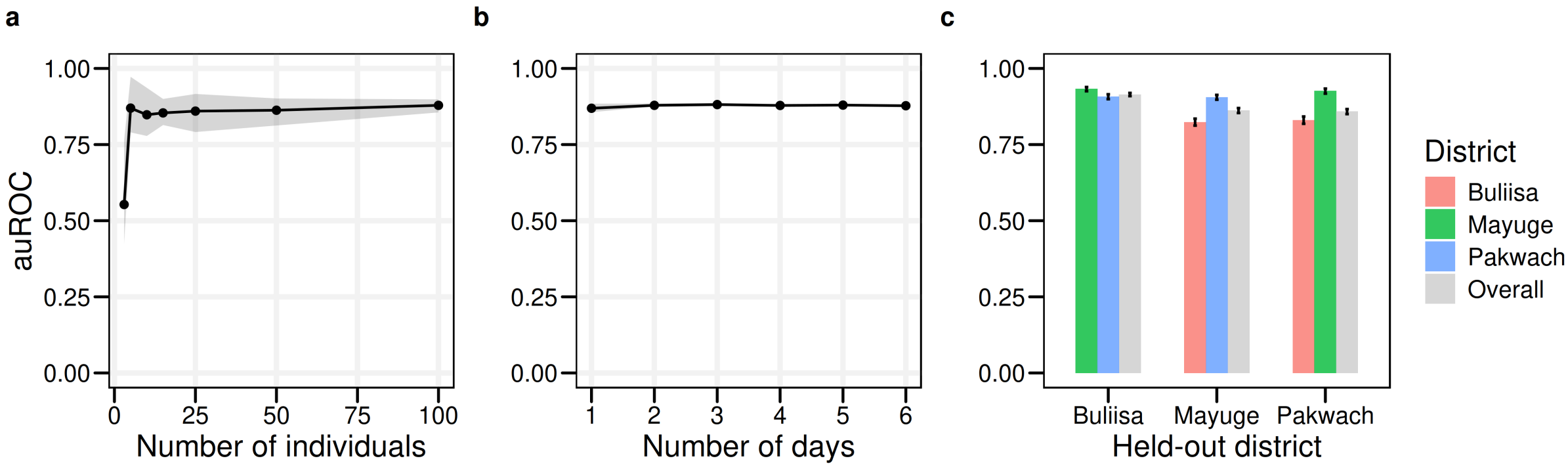

Fig. S12: **Minimum data needs for estimating the spatial decay function of open water contact. a** auROC by number of individuals used to estimate the spatial decay. **b** auROC by number of GPS logger days used to estimate the spatial decay. **c** Evaluating performance on held-out districts. For (**a**,**b**) estimation was performed by randomly sampling from the training data three times and evaluating the auROC on held-out test data. For (**c**), models were estimated on the full data from one district, and performance was evaluated separately for each of the held-out districts and for both held-out districts jointly (overall columns). The x-axis label in (**c**) indicates the held-out district. Shaded grey areas and vertical bars represent 95% credible intervals.

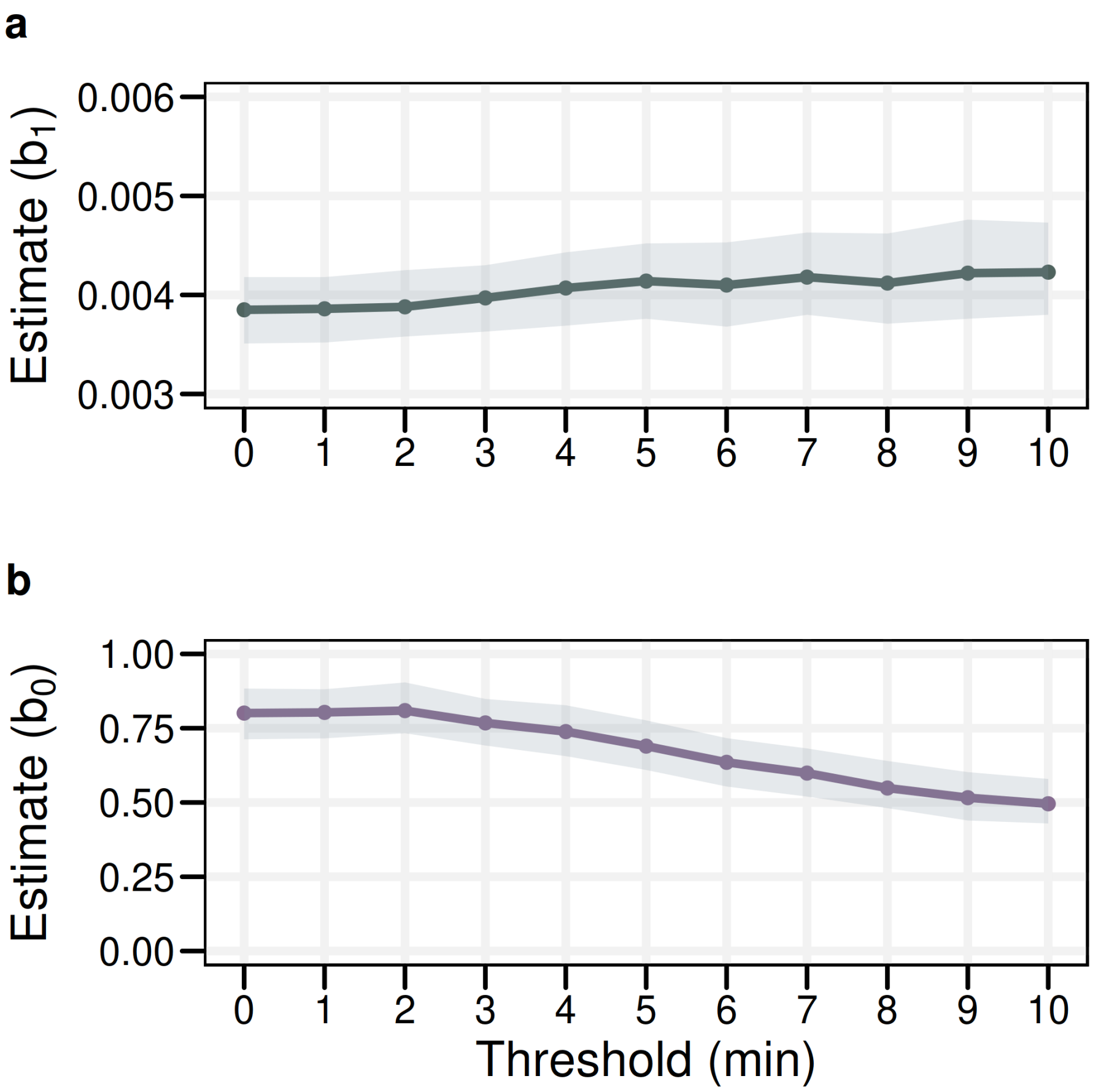

Fig. S13: **The impact of removing short-duration open water site and tap/borehole usage events.** The threshold value on the x-axis indicates the cut-off value below which events were removed (with a cut-off of zero denoting the dataset used for the main models, where no events were removed). Shaded grey areas represent 95% credible intervals.

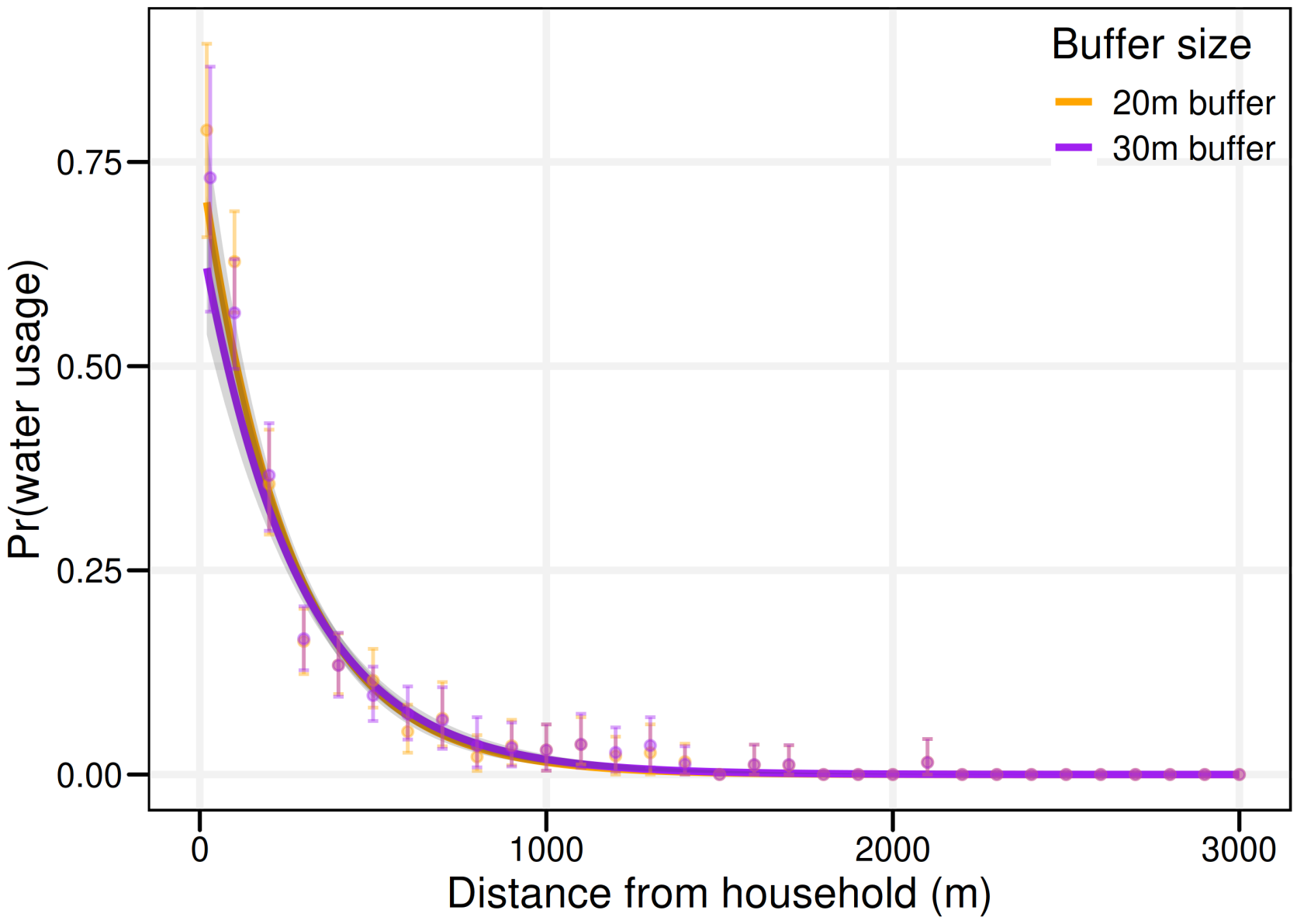

Fig. S14: **Robustness to buffer size choice.** Robustness of the spatial decay model of open water site contact to variation in the buffer size used to derive open water contact events from the GPS logger data. The yellow line represents the spatial decay model results using a 20 m buffer (buffer size used for all analyses). The purple line represents alternative results with a slightly larger buffer of 30 m. Error bars represent the 2.5th and 97.5th percentile ranges calculated using 2000 replicates and plotted in 100 m increments. Points represent the respective means.

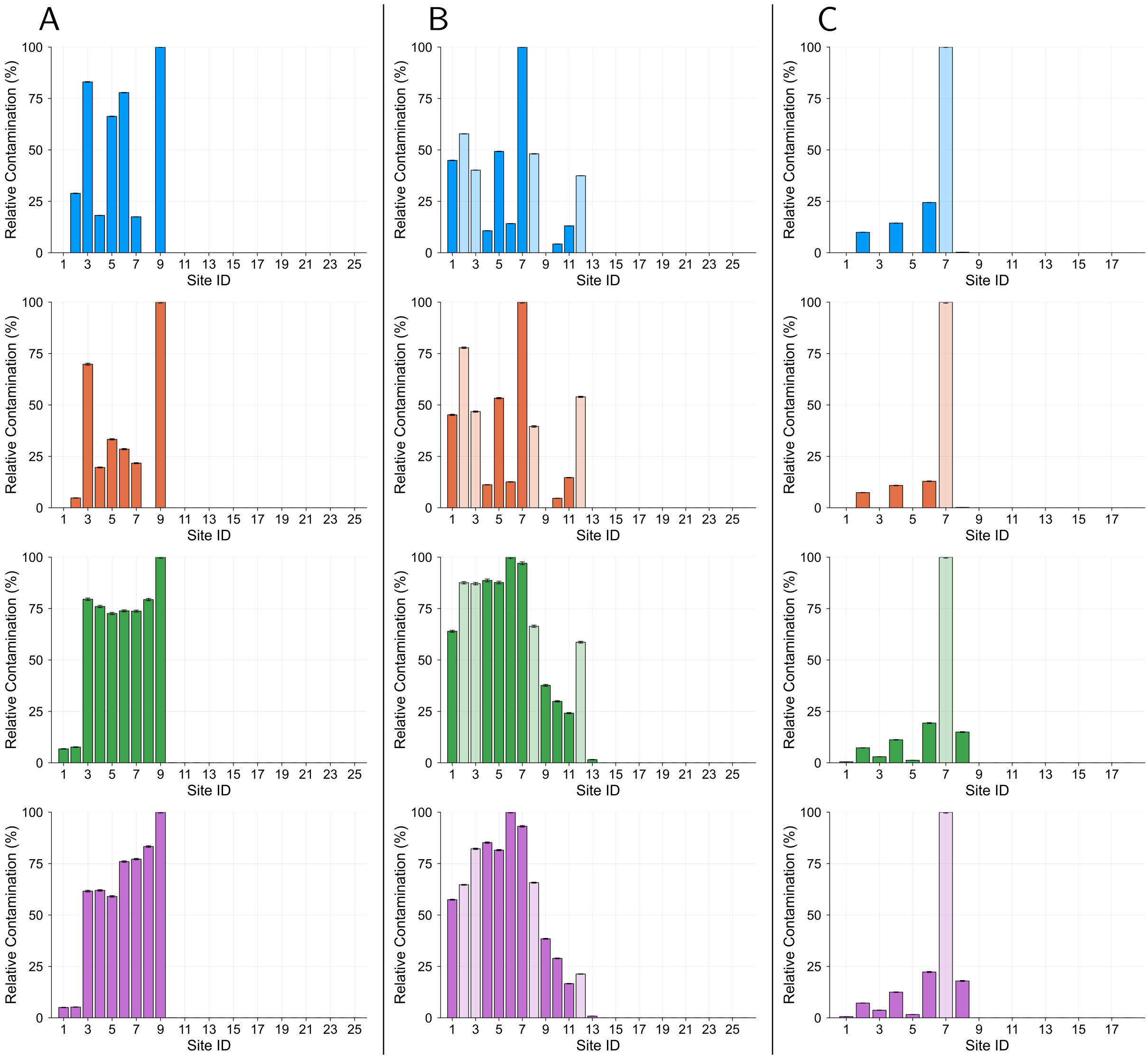

Fig. S15: **Relative miracidial contamination by site, district, and visitation rule.** The panels display measures of human-to-snail force of infection (approximated via cumulative miracidial contamination) at each site over 365 days, averaged over 100 replicates. To provide a measure of site relevance for transmission, we expressed values for each site as a percentage of the most contaminated site within each district (**A** Pakwach, **B** Buliisa, and **C** Mayuge). The four colours correspond to the different visitation rules (blue—rule (1), orange—rule (2), green—rule (3), and purple—rule (4)), illustrating that the identification of high-transmission sites is sensitive to the underlying open water site visitation assumptions used. Each vertical bar represents one open water site with the x-axis label denoting its site ID. Lighter-coloured bars denote open water sites where no snails were observed. While these sites do not currently contribute to transmission, high miracidial input indicates a potential risk if snail populations were to become established, highlighting priorities for surveillance. Error bars indicate 95% confidence intervals across 100 simulation replicates ($\pm 1.96 \times \mathrm{SD}/\sqrt{n}$) of the relative contamination values. Note that rule (1) is deterministic, so between-replicate variability for this rule is effectively zero.

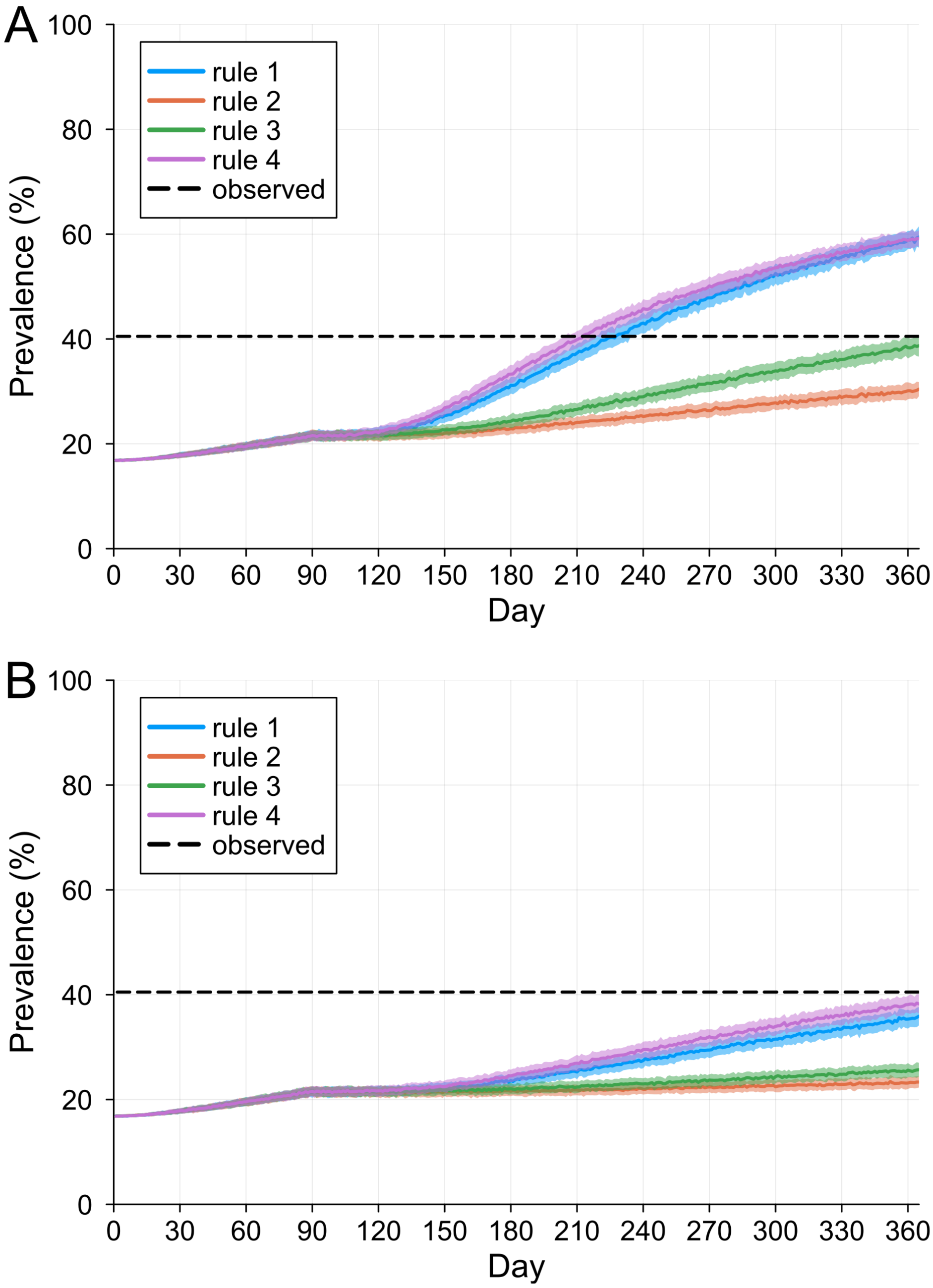

Fig. S16: **Sensitivity analysis for infection dynamics.** We varied the aggregation parameter as per Graham et al. [3], who presented the SCHISTOX model. In **A** the parameter is set to 0.14, and in **B** it is set to 0.04. A lower value is appropriate for low-prevalence settings. Fig. 6 shows the results with the parameter set to 0.24, the higher end of the range [3]. The simulations start from approximately the post-treatment infection prevalence (16.9%) and the dashed black line indicates the reinfection prevalence (40.5%) observed one year post-baseline. The shaded areas represent 95% confidence intervals, calculated based on 100 replicates for each decision rule for 365 days.

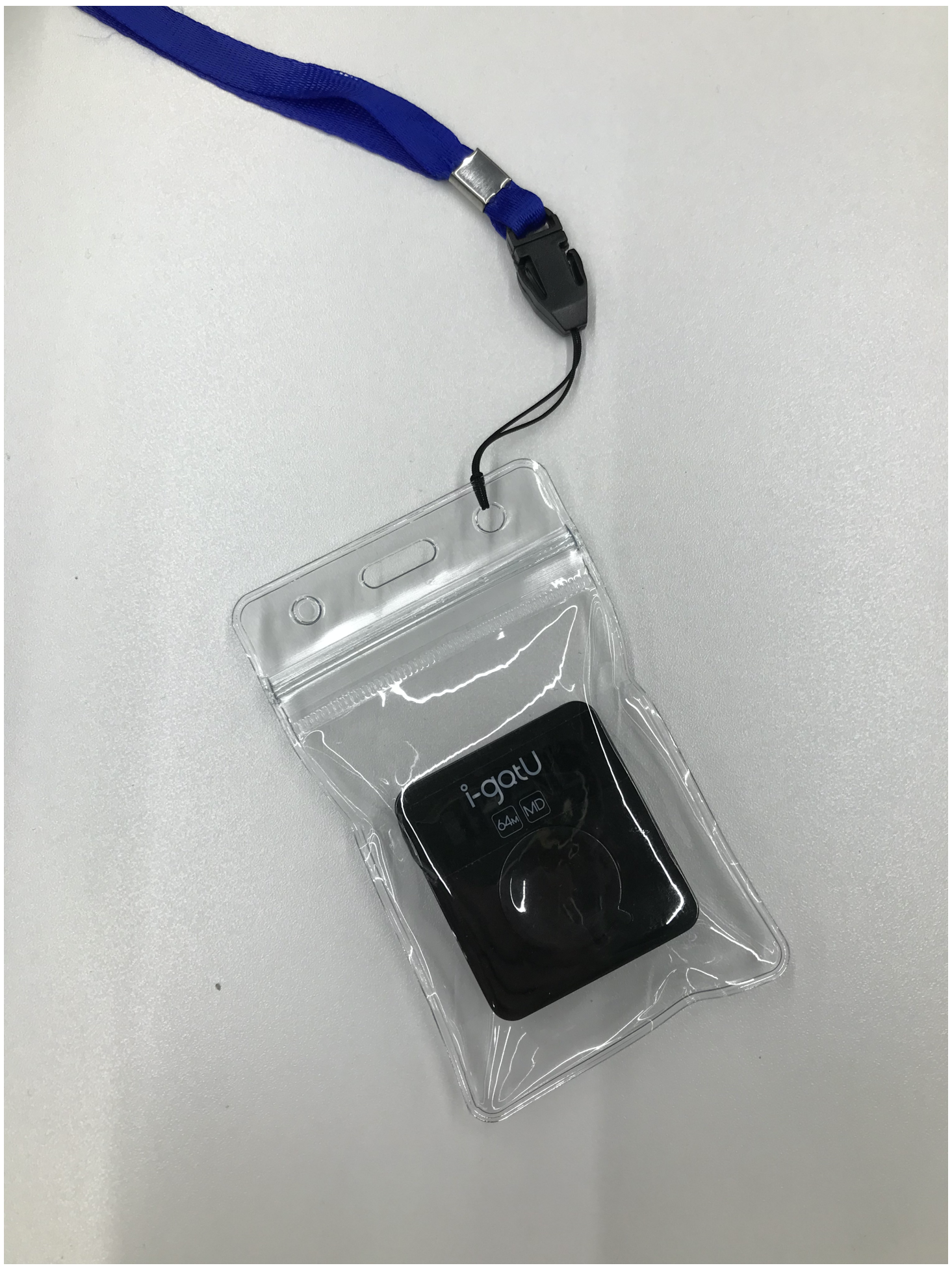

Fig. S17: **GPS logger in waterproof pouch and with attached lanyard.**

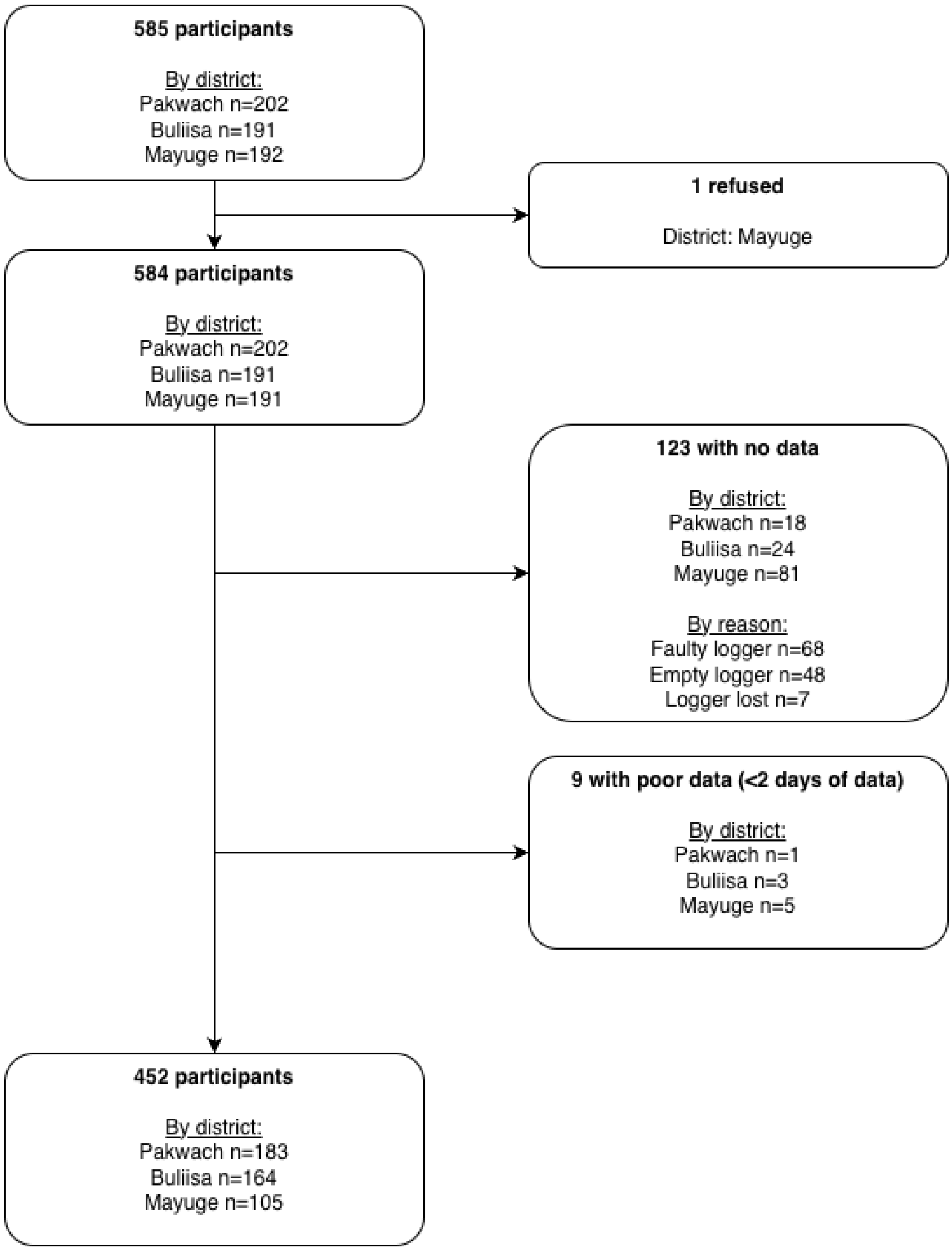

Fig. S18: **Participant flowchart.** Loggers were classified as being faulty when no data could be retrieved due to complete device failure. Loggers were classified as being empty when loggers could still be pulled using a computer, but contained no data.

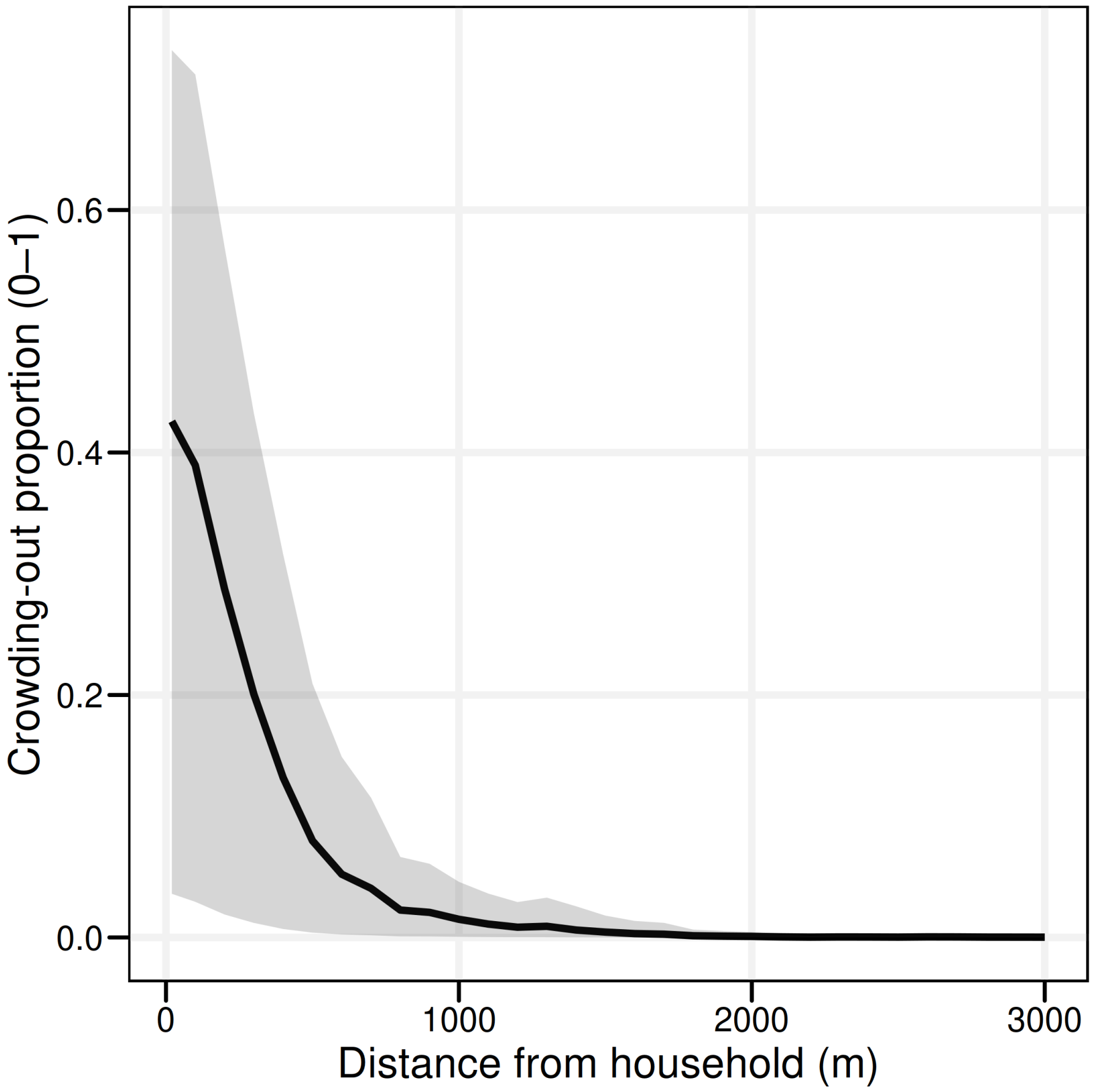

Fig. S19: **Crowding out of open water site usage duration over household distance.** Estimated percentage reduction in the duration of open water site usage over household distance. This was estimated by comparing durations of open water site usage, estimated in the presence of taps/boreholes, to a counterfactual scenario where no taps/boreholes were present, as shown in Fig. S11. Shaded grey areas represent 95% credible intervals.

S3 Supplementary tables

Table S1: Summary of key participant characteristics.

	Open water site usage			Tap/borehole usage		
Variable	No (n= 163)	Yes (n= 289)	P-value	No (n= 302)	Yes (n= 150)	P-value
Gender = male (%)	85 (52.1)	141 (48.8)	0.56	150 (49.7)	76 (50.7)	0.92
Age ≥ 15 years (%)	88 (54.0)	166 (57.4)	0.54	171 (56.6)	83 (55.3)	0.87
Occupation (%)			<0.01			0.60
none	97 (59.5)	162 (56.1)		176 (58.3)	83 (55.3)	
fishing	7 (4.3)	34 (11.8)		24 (7.9)	17 (11.3)	
fishmongering	2 (1.2)	20 (6.9)		15 (5.0)	7 (4.7)	
farming	47 (28.8)	46 (15.9)		65 (21.5)	28 (18.7)	
other	10 (6.1)	27 (9.3)		22 (7.3)	15 (10.0)	
Household drinking water source = improved (%)	44 (27.0)	182 (63.0)	<0.01	125 (41.4)	101 (67.3)	<0.01
Distance to the closest site (m) (median [IQR])	386.0 [247.1, 648.6]	91.6 [58.9, 247.0]	<0.01	230.8 [79.6, 418.4]	98.2 [60.4, 310.7]	<0.01
Distance to the closest tap/borehole (m) (median [IQR])	396.2 [135.2, 705.0]	179.3 [82.9, 404.5]	<0.01	355.4 [154.8, 863.1]	85.2 [47.2, 176.9]	<0.01
Radius of gyration (m) (median [IQR])	363.3 [171.9, 1247.8]	347.0 [127.8, 1032.4]	0.18	369.2 [152.6, 1092.6]	314.8 [134.7, 1065.3]	0.55

Notes: For binary variables, numbers and proportions [N (%)] are shown, and Chi-squared tests were used to test for group differences. For all non-normally distributed variables, median and interquartile range (median [IQR]) are shown, and Kruskal–Wallis Rank Sum Tests were performed to test for group differences.

Table S2: Logistic regression models predicting open water site usage.

Model	ELPD difference	Standard error	P-value
**Open water contact models**			
Distance to water sites	0.0	0.0	–
Closest water site (y/n)	-128.9	27.1	<0.001
Distance to water sites <430m (y/n)	-131.5	16.3	<0.001
Self-reported water contact (y/n), activity match (y/n), mobility tercile	-371.1	28.7	<0.001
Gender, age (<15, ≥15 years), occupation category, improved household drinking water source (y/n)	-374.6	27.7	<0.001

Notes: Bayesian logistic regression models using distance, sociodemographic proxies, or behavioural proxies (see Methods) to predict open water site usage. We evaluated model performance using expected log pointwise predictive density (ELPD), estimated via Bayesian leave-one-out cross-validation. Negative ELPD values signify lower predictive density, indicating that a model performs worse than the reference model (best-performing model). The p-value was estimated based on a two-sided comparison (t-test) to the best-performing model.

Table S3: Model selection for spatial decay models.

Model	ELPD difference	Standard error	P-value
**Open water contact models**			
District	0.0	0.0	–
Gender	-11.1	6.7	0.098
Activity match	-11.8	6.9	0.087
Global	-13.0	6.2	0.036
Age	-13.7	6.9	0.047
**Tap/borehole models**			
District	0.0	0.0	–
Safe water supply type	-7.7	5.7	0.177
Global	-10.1	6.0	0.092
Activity match	-10.3	5.8	0.076
Gender	-10.5	5.9	0.075
Age	-11.8	5.8	0.042
Occupation category	-13.9	5.9	0.018

Notes: Model comparison using expected log pointwise predictive density (ELPD), estimated via Bayesian leave-one-out cross-validation, to select the best performing spatial decay model for predicting open water site and tap/borehole usage. For all models other than the global model, slopes and intercepts were varied by the variable denoted in the 'model' column. ELPD differences are relative to the best-performing model. Negative ELPD values signify lower predictive density, indicating that a model performs worse than the reference model (best-performing model). The p-value was estimated based on a two-sided comparison (t-test) to the best-performing model. For the open water contact, due to convergence issues, it was not possible to include a spatial decay model with occupation as a covariate.

Table S4: Comparison of participants with sufficient GPS data versus those with missing data and the wider SchistoTrack study population.

Variable	GPS complete (n=452)	GPS missing (n=132)	Not selected for GPS (n=379)	P-value
Gender = male (%)	226 (50.0)	54 (40.9)	149 (39.3)	0.01
Age ≥ 15 years (%)	254 (56.2)	75 (56.8)	214 (56.5)	0.99
Self-reported water contact = yes (%)	231 (51.1)	58 (43.9)	169 (44.6)	0.12
Occupation (%)				0.19
none	259 (57.3)	84 (63.6)	225 (59.4)	
fishing	41 (9.1)	10 (7.6)	24 (6.3)	
fishmongering	22 (4.9)	2 (1.5)	19 (5.0)	
farming	93 (20.6)	31 (23.5)	73 (19.3)	
other	37 (8.2)	5 (3.8)	38 (10.0)	
Distance to the closest water site (m) (median [IQR])	206.1 [71.3, 386.3]	289.5 [148.6, 401.7]	228.3 [69.8, 416.1]	0.03
Household drinking water source = improved (%)	226 (50.0)	33 (25.0)	160 (42.2)	<0.01
District (%)				<0.01
Mayuge	105 (23.2)	86 (65.2)	129 (34.0)	
Buliisa	164 (36.3)	27 (20.5)	132 (34.8)	
Pakwach	183 (40.5)	19 (14.4)	118 (31.1)	

Notes: For binary variables, numbers and proportions [N (%)] are shown, and Chi-squared tests were used to test for group differences. For all non-normally distributed variables, median and interquartile range (median [IQR]) are shown, and Kruskal–Wallis Rank Sum Tests were performed to test for group differences.

Table S5: Comparison of participants by number of recorded GPS points, grouped into terciles.

Variable	**GPS data points per day (tercile)**			
	Bottom (n= 151)	Middle (n= 151)	Top (n= 150)	P-value
Gender = male (%)	70 (46.4)	76 (50.3)	80 (53.3)	0.48
Age ≥ 15 years (%)	77 (51.0)	80 (53.0)	97 (64.7)	0.04
Self-reported water contact = yes (%)	66 (43.7)	78 (51.7)	87 (58.0)	0.05
Occupation (%)				0.29
none	99 (65.6)	84 (55.6)	76 (50.7)	
fishing	8 (5.3)	17 (11.3)	16 (10.7)	
fishmongering	6 (4.0)	9 (6.0)	7 (4.7)	
farming	28 (18.5)	29 (19.2)	36 (24.0)	
other	10 (6.6)	12 (7.9)	15 (10.0)	
Distance to the closest site (m) (median [IQR])	213.4 [71.9, 395.9]	200.5 [74.6, 353.0]	204.6 [69.9, 385.6]	0.97
Household drinking water source = improved (%)	75 (49.7)	76 (50.3)	75 (50.0)	0.99

Notes: All participants were grouped into district-specific terciles (bottom, middle, top) based on their average number of GPS points per day. For binary variables, numbers and proportions [N (%)] are shown, and Chi-squared tests were used to test for group differences. For all non-normally distributed variables, median and interquartile range (median [IQR]) are shown, and Kruskal–Wallis Rank Sum Tests were performed to test for group differences.

Table S6: Differences in spatial decay model predictive performance across sociodemographic factors.

Variable	<Q25 (n= 81)	Q25–Q75 (n= 187)	>Q75 (n= 184)	P-value
Gender = male (%)	47 (58.0)	86 (46.0)	93 (50.5)	0.19
Age ≥15 years (%)	38 (46.9)	117 (62.6)	99 (53.8)	0.04
Occupation (%)				<0.01
none	49 (60.5)	97 (51.9)	113 (61.4)	
fishing	12 (14.8)	20 (10.7)	9 (4.9)	
fishmongering	2 (2.5)	16 (8.6)	4 (2.2)	
farming	14 (17.3)	33 (17.6)	46 (25.0)	
other	4 (4.9)	21 (11.2)	12 (6.5)	
Household drinking water source = improved (%)	58 (71.6)	121 (64.7)	47 (25.5)	<0.01
Distance to the closest site (m) (median [IQR])	102.8 [68.8, 223.3]	91.1 [57.7, 261.0]	335.2 [176.4, 624.2]	<0.01
Distance to the closest tap/borehole (m) (median [IQR])	193.2 [88.1, 315.2]	176.7 [63.4, 386.8]	398.8 [130.1, 743.7]	<0.01
Radius of gyration (m) (median [IQR])	386.2 [181.1, 929.9]	318.5 [121.9, 1074.7]	362.6 [159.8, 1175.1]	0.65

Notes: This table shows how predictive accuracy of the district-specific spatial decay model predicting open water site usage varied across sociodemographic factors. This was investigated by the quantile of the number of correctly predicted open water sites per individual, where the median was 92.3% (i.e. usage of 92.3% of all open water sites correctly predicted). Q25 encompasses individuals below the 25th percentile (84.6% correctly predicted). Q75 encompasses individuals $\geq 75^{\text{th}}$ percentile (>99.9% correctly predicted).

Table S7: **Overview of spatial decay models presented in this paper.** *Notes:* w = open water site usage, t = tap/borehole usage. Across formulas, i denotes the individual, k denotes the open water site or tap/borehole, and j denotes the covariate described in the covariates column.

**Type of model**	**Equation**	**Outcome**	**Decay function**	**Additional covariate(s)**	**Priors**	**Link function**	**In-text reference**
**Basic decay models (to select decay function *f(d)*)**							
Exponential decay model	$f(d) = b_0 \cdot e^{-b_1 \cdot d}$	w, t	Exponential	none	Unif$(0,1)$ for b_1	Identity link	-
Power law decay model	$f(d) = \frac{1}{(1+\beta \cdot d)^{-\gamma}}$	w, t	Power law	none	Unif$(0,1)$ for β, Unif$(0,20)$ for γ	Identity link	-
Hill function model	$f(d) = \frac{b_0}{1+\left(\frac{d}{b_1}\right)^n}$	w, t	Hill function	none	Unif$(0,1)$ for b_0, Normal$(0,5)$ for b_1, Normal$(1,2)$ for n	Identity link	-
**Decay models with covariates (to select covariates)**							
Decay models with one covariate j added	$Pr(\text{response}_{i,j,k} = 1) = b_{0,j} \cdot f_j(d_{i,j,k})$	w, t	Exponential	district, gender, age (<15 years, $\geq$ 15 years), occupation category, activity match for w models and district, gender, age (<15 years, $\geq$ 15 years), occupation category for t models	–	Identity link	Eq. 3
**Decay models of spatial factors influencing open water site usage (Fig. 3a–d)**							

Continued on next page

Table S7 – continued from previous page

Type of model	Equation	Outcome	Decay function	Additional covariate(s)	Priors	Link function	In-text reference
Distance-based decay model	$Pr(\text{response}_{i,j,k} = 1) = b_{0,j} \cdot f_j(d_{i,j,k})$	w	Exponential	district	Unif(0,1) for a_1, Unif(0,1) for b_1	Identity link	Eq. 3
Competing opportunity model	$Pr(\text{response}_{i,j,k} = 1) = b_{0,j} \cdot f_j(N_{i,j,k})$	w	Exponential	site order	Unif(0,1) for a_1, Unif(0,1) for b_1	Identity link	Eq. 3
Human-mobility tercile-based spatial decay model	$Pr(\text{response}_{i,j,k} = 1) = b_{0,j} \cdot f_j(d_{i,j,k})$	w	Exponential	district-specific mobility tercile	Unif(0,1) for a_1, Unif(0,1) for b_1	Identity link	Eq. 3
Crowding out model	$Pr(\text{open water site usage}_{i,k} = 1) = b_0 \cdot f_j(d_{i,k}) \cdot [1 - \alpha \cdot P(\text{tap/borehole usage}_i)]$	w	Exponential	tap/borehole usage probability	Unif(0,1) for a_1, Unif(0,1) for b_1, Unif(0,1) for α	Identity link	Eq. 5
**Decay models predicting open water site usage duration (Fig. 4b)**							
Distance-based decay model of duration	$\text{E}(t_{i,k}) = b_0 \cdot f(d_{i,k})$	w	Exponential	district	Unif(0,1) for b_1, Normal(1,9) truncated at [0,30] for b_0	Identity link	Eq. 4

References

[1] Reitzug F, Kabatereine NB, Byaruhanga AM, Besigye F, Nabatte B, Chami GF. Current water contact and *Schistosoma mansoni* infection have distinct determinants: a data-driven population-based study in rural Uganda. Nature Communications. 2024 Nov;15(1). https://doi.org/10.1038/s41467-024-53519-4.

[2] World Health Organization. WHO guideline on control and elimination of human schistosomiasis. Geneva: World Health Organization; 2022. Section: xxi, 108 p. Available from: https://apps.who.int/iris/handle/10665/351856.

[3] Graham M, Ayabina D, Lucas TC, Collyer BS, Medley GF, Hollingsworth TD, et al. SCHISTOX: An individual based model for the epidemiology and control of schistosomiasis. Infectious Disease Modelling. 2021;6:438–447. https://doi.org/10.1016/j.idm.2021.01.010.
