# Supplementary material for: Predicting schistosome transmission in rural Uganda using water contact data from wearable GPS devices
Source: Nat Health. 2026 May 22;1(8):850–64. doi: 10.1038/s44360-026-00118-w (PMC13433293; doi:10.1038/s44360-026-00118-w)
Supplement: Supplementary file 2 [file 44360_2026_118_MOESM2_ESM.pdf]

nature portfolio

Corresponding author(s): Goylette F. Chami

Last updated by author(s): Mar 6, 2026

Reporting Summary

Nature Portfolio wishes to improve the reproducibility of the work that we publish. This form provides structure for consistency and transparency in reporting. For further information on Nature Portfolio policies, see our Editorial Policies and the Editorial Policy Checklist.

Statistics

For all statistical analyses, confirm that the following items are present in the figure legend, table legend, main text, or Methods section.

n/a	Confirmed	
☐	☒	The exact sample size (*n*) for each experimental group/condition, given as a discrete number and unit of measurement
☒	☐	A statement on whether measurements were taken from distinct samples or whether the same sample was measured repeatedly
☐	☒	The statistical test(s) used AND whether they are one- or two-sided<br>*Only common tests should be described solely by name; describe more complex techniques in the Methods section.*
☐	☒	A description of all covariates tested
☐	☒	A description of any assumptions or corrections, such as tests of normality and adjustment for multiple comparisons
☐	☒	A full description of the statistical parameters including central tendency (e.g. means) or other basic estimates (e.g. regression coefficient) AND variation (e.g. standard deviation) or associated estimates of uncertainty (e.g. confidence intervals)
☐	☒	For null hypothesis testing, the test statistic (e.g. *F*, *t*, *r*) with confidence intervals, effect sizes, degrees of freedom and *P* value noted<br>*Give P values as exact values whenever suitable.*
☐	☒	For Bayesian analysis, information on the choice of priors and Markov chain Monte Carlo settings
☐	☒	For hierarchical and complex designs, identification of the appropriate level for tests and full reporting of outcomes
☐	☒	Estimates of effect sizes (e.g. Cohen's *d*, Pearson's *r*), indicating how they were calculated

Our web collection on statistics for biologists contains articles on many of the points above.

Software and code

Policy information about availability of computer code

Data collection	Open Data Kit (ODK) was used for data collection (ODK tablet app version v2021.3.1, ODK XLSForm Offline v1.9.0)
Data analysis	R version 4.1.0 was used for the statistical data analysis. Julia version 1.11.3 was used for building an individual-based transmission model.

For manuscripts utilizing custom algorithms or software that are central to the research but not yet described in published literature, software must be made available to editors and reviewers. We strongly encourage code deposition in a community repository (e.g. GitHub). See the Nature Portfolio guidelines for submitting code & software for further information.

Data

Policy information about availability of data

All manuscripts must include a data availability statement. This statement should provide the following information, where applicable:

- Accession codes, unique identifiers, or web links for publicly available datasets
- A description of any restrictions on data availability
- For clinical datasets or third party data, please ensure that the statement adheres to our policy

Due to the highly sensitive nature of the GPS logger data, restrictions from the data protection impact assessment, and ongoing nature of the SchistoTrack cohort, these data are not publicly available. Extensive metadata for all variables have been provided in the manuscript and supplement.

Research involving human participants, their data, or biological material

Policy information about studies with human participants or human data. See also policy information about sex, gender (identity/presentation), and sexual orientation and race, ethnicity and racism.

Reporting on sex and gender	We exclusively use the term gender, following the definitions laid out in the 'Sex and Gender Equity in Research – SAGER – guidelines' because in this study, information was elicited via self-reports and we sought to understand how gender (shaped by social and cultural norms) influences water contact behaviour.
Reporting on race, ethnicity, or other socially relevant groupings	We recorded data on self-reported occupation, gender, and age
Population characteristics	See behavioral & social study design section below
Recruitment	For the SchistoTrack baseline, we randomly sampled a total of 1,459 households (n=2885)—approximately 40 per village—in 38 villages across three districts, using village registers or mass drug administration (MDA) records. All households with at least one child and one adult residing in the village for at least six months of the year were eligible. For the GPS logger study presented in this paper, we subsequently sampled four villages per district (12 in total) based on their levels of water contact and occupational fishing, determined predominantly by the presence of a beach or landing site. None of the selected villages had piped water. A maximum of 50 participants per village were recruited, with a target of one adult–child pair (an adult [≥18 years] and one child [5–17 years]) per household.
Ethics oversight	Data collection and use were reviewed and approved by Oxford Tropical Research Ethics Committee (OxTREC 509-21), Vector Control Division Research Ethics Committee of the Uganda Ministry of Health (VCDREC146), and Uganda National Council of Science and Technology (UNCST HS 1664ES).

Note that full information on the approval of the study protocol must also be provided in the manuscript.

Field-specific reporting

Please select the one below that is the best fit for your research. If you are not sure, read the appropriate sections before making your selection.

☐ Life sciences ☒ Behavioural & social sciences ☐ Ecological, evolutionary & environmental sciences

For a reference copy of the document with all sections, see nature.com/documents/nr-reporting-summary-flat.pdf

Behavioural & social sciences study design

All studies must disclose on these points even when the disclosure is negative.

Study description	Cross-sectional, quantiative study of 452 individuals aged 5-82 years in Eastern and Western Uganda. We collected socio-demographics, biomedical information (including schistosome infection information), WASH information, environmental data, self-reported water contact data, and GPS-logger derived water contact, tap/borehole usage and human movement.
Research sample	Subsample of n=452 was drawn from a population-representative sample of n=2885. Participants were aged 5-82 years and 50% (226/452) were male and 50% (226/452) were female.
Sampling strategy	We selected 452 participants from the larger SchistoTrack study (n=2885) which had been randomly selected from village registers or MDA records. For the GPS logger study, participants were sampled from the SchistoTrack study study participant list to ensure a balance of age and gender, and fishermen were oversampled to ensure a diverse representation of open water contact and mobility patterns. We excluded participants that were physically immobile and thus unable to have water contact and we also excluded children who were sent away to boarding school. All participants were unaware of their infection status when they entered the GPS logger study.
Data collection	Household data collection was done by trained surveyors (either from Kampala or locally recruited) using tables and ODK software. Household-level information was collected prior to clinical data collection and participants were unaware of their infection status. All clinical assessments were conducted by technicians/nurses from the district or Kampala in mobile in-the-field labs. Wearable GPS loggers (i-gotU GT-600; Mobile Action Technology, Taiwan) were used to record the location data of participants at two-minute intervals between 5 a.m. and 8 p.m. local time over approximately ten consecutive days.
Timing	Data collection took place in January to February 2022 in Pakwach, Buliisa and Mayuge.
Data exclusions	From the 585 participants that were originally recruited were recruited for the study 133 were excluded from the analysis, mainly to device failure and loggers not being returned by participants after being handed out in Pakwach. After excluding participants with fewer than two complete days of GPS data. A flow chart detailing any data exclusions is provided in Fig S18.
Non-participation	One participants that was selected for the GPS logger study refused (see Flowchart Fig S18).

Randomization	Not applicable as this was an observational study.

Reporting for specific materials, systems and methods

We require information from authors about some types of materials, experimental systems and methods used in many studies. Here, indicate whether each material, system or method listed is relevant to your study. If you are not sure if a list item applies to your research, read the appropriate section before selecting a response.

Materials & experimental systems

n/a	Involved in the study
☒	☐ Antibodies
☒	☐ Eukaryotic cell lines
☒	☐ Palaeontology and archaeology
☒	☐ Animals and other organisms
☐	☒ Clinical data
☒	☐ Dual use research of concern
☒	☐ Plants

Methods

n/a	Involved in the study
☒	☐ ChIP-seq
☒	☐ Flow cytometry
☒	☐ MRI-based neuroimaging

Clinical data

Policy information about clinical studies

All manuscripts should comply with the ICMJE guidelines for publication of clinical research and a completed CONSORT checklist must be included with all submissions.

Clinical trial registration	Not applicable as this was an observational study.
Study protocol	Not applicable as this was an observational study.
Data collection	Data collection took place in January to February 2022 in Pakwach, Buliisa and Mayuge.
Outcomes	Primary exposure outcome was open water contact, primary infection outcome was schistosome infection, as indicated by Kato-Katz stool microscopy.

Plants

Seed stocks	Not applicable
Novel plant genotypes	Not applicable
Authentication	Not applicable
