# Supplementary material for: Predicting schistosome transmission in rural Uganda using water contact data from wearable GPS devices
Source: Nat Health. 2026 May 22;1(8):850–64. doi: 10.1038/s44360-026-00118-w (PMC13433293; doi:10.1038/s44360-026-00118-w)
Supplement: Supplementary file 3 [file 44360_2026_118_MOESM3_ESM.pdf]

nature portfolio

Peer Review File

Predicting schistosome transmission in rural Uganda using water contact data from wearable GPS

Corresponding Author: Professor Goylette Chami

This file contains all reviewer reports in order by version, followed by all author rebuttals in order by version.

Version 0:

Reviewer comments:

Reviewer #1

(Remarks to the Author)
In this work Reitzug and colleagues study the role of spatial distance in the usage of open water vs. tap/boreholes using a GPS tracking dataset collected from 452 study participants in Uganda. Their main result is that distance is a strong predictor of water usage site, with the large majority of water usage occurring within a short radius around participant households (~1km). The authors also propose an analysis of the impact of tap/borehole placement strategies on the avoidance of usage of open water sites.

Small-scale human mobility patterns can have significant consequences on infectious disease dynamics, and this study adds to a yet relatively limited set of studies that use GPS data to characterize these patterns, especially for neglected tropical diseases such as schistosomiasis. There are previous examples of such studies, however the strength of this study lies in its comparatively large sample size. As such, the work has potential to be of interest to the readership of the Journal. There are however several points which I believe currently strongly limit the results of the analysis in its current state, and therefore limit the potential reach of its conclusions.

Major points

1. Framing
From my take, there seems to be a mismatch between the framing of the work between the title, the introduction, the results and the discussion. The title and results sections put emphasis on water usage patterns per se, in relation to existing knowledge of human mobility patterns in general. In addition unsafe water usage is important for many diseases, as noted by the authors. This is reflected in the stated aims of the paper in L. 123-127 which do not mention a specific disease application. Almost all the results section focuses on water usage patterns, with a very minor section on epidemiologic outcomes in L. 530-537. On the other hand, the introduction and discussion put important emphasis on the specific aspects of water contacts and schistosomiasis, relating the outcomes of the study to schistosomiasis modeling studies, and less to the general aspects of human mobility of water usage vs. other types of mobility.

I understand the link between the two, as rightly mentioned by the authors in the introduction, I however believe that the very limited emphasis on epidemiologically-relevant outcomes in the results strongly limits the interpretation of the results for public health policy. A specific point on the reinfection data is given below. Linking the water usage results to schistosomiasis in the study area may be difficult because water contacts are not necessarily exposures if there are no (shedding) snail intermediate hosts.

I would therefore suggest the authors consider re-aligning the framing of the study on whether it is schistosomiasis-specific or not, possibly re-dimensioning the focus given on the disease in the different sections.

2. Modeling methods
2.1 Model formulations
In equation 2, it is unclear to me what the modeled outcome is. Is it whether there was any occurrence of water usage of a specific place by individual I throughout the study period? This is confusing to me in L. 1206-1210 which seem to indicate that each individual can only choose one water site at each time, although there may be multiple usages during the study

period. From what I understood the number of days of participation is not the same for all participants. Is this accounted for in the data likelihood?

Could the authors comment on why a discrete distribution is used to model average daily contact duration during the study period?

2.2 Model comparison

The core of the results builds on the comparison of different models predicting water usage and duration. Throughout the paper the authors base these comparisons on a testing set of 15% of the data, except for the choice of decay function which is based on ELPD. Both of these metrics aim at assessing predictive performance. I would therefore suggest the authors choose one and use the same throughout the paper. If would personally suggest ELPD, having a testing dataset is also fine. I would however not base all testing on a single holdout, but instead do a K-fold cross-validation scheme with proper accounting of the relative number of participants per district. I would also urge the authors to report uncertainty intervals and all metrics (ELPD, auROC, sensitivity, specificity), and detail in the methods the methodology to discriminate between models. There are in fact multiple instance where the terminology is unclear to me (for instance in saying that an auROC of 0.890 is larger than 0.888 without reporting uncertainty intervals and a definition of what a significant difference is, which to me in this case is effectively equivalent and would not choose a specific model based on it). I believe this is particularly important for the crowding-out model on which the simulations are based, as if the data does not support it better than the simple spatial decay model I believe it undermines its use in the simulations section.

I also wondered why the different decay functions were not compared in the other model version (Fig3. B-d), as these different model formulations may turn out to support other functions.

3.3 Simulations

The validity of the simulation study depends on how well the mechanistic effects producing water usage shifts area captured by the model. In its current state, the data does not seem to support the crowding-out model in a clear way (see point above), which makes me doubt on the reliability of its use for simulations. I would assume that some types of water usages may be more avoidable than others in the presence of a tap/borehole, and that this would be very valuable to account for in this analysis, possibly through the use of covariates. Another point is that it does not seem to me that the presence of tap water in the household itself was accounted for here, which may be an important effect modifier of the the addition of new tap/boreholes. I would ask the authors to comment on these points as to wether they believe their simulations reliable capture the variability in effect of adding tap/boreholes in shifting water usage patterns.

3. Validation of water usage results

Although the authors use the term “water usage”, it technically consists of inferences made from GPS tracks which cannot be considered as perfect (Paz-Soldan et al., 2013). The authors have published detailed work on water usage practices in the area, from what I understand from the same participants. I was therefore wondering if some aspects of the GPS-derived water usage data could be validate against questionnaire data, for instance the proportion reporting any contact during the season of the study. This would greatly strengthen the results in my opinion. Regardless, I think that more explicitly mentioning the limitations of the GPS data to infer water usage in the limitations paragraph would help the reader assess the study.

4. Schistosomiasis re-infection data

It was unclear to me how to interpret the results sentences on the reinfection data. This seems to be an important point, as it supports the public health relevance of the whole water usage study. Could the authors expand on these results more in terms of the aspects of water usage that relate to reinfection probability.

Other points

1. Could the authors please provide a full mathematical description of the models in the analysis, a table with equations for all spatial decay models would be very helpful.

2. Crowding out model. It was unclear to me what the probability of tap/borehole usage corresponds to in equation 5. Is it to the closes tap for person i?

3. Comparison of tap/borehole to logistic regression models. As almost all tap usages are with the closest one, I was curious to know how the spatial decay model fairs against logistic regression models.

4. Accounting for in-house tap water. From table S1 it seems like a good proportion of households had tap water in their home, which I suppose can have an important impact on water usage outside the household. Was this accounted for in the models? May this explain part of the variability in any water usage, which the models seem to consistently over-estimate (figure 6c).

5. Posterior retrodictive checks. I would like to see posterior retrodictive checks of the number of water contact points used by individual to compare to figure 1b.

6. Buffer sensitivity analysis. The authors use 30m to define a buffer for water usage. I would encourage the authors to provide a sensitivity analysis of buffer width on the main results of the paper.

7. Limitations. In the limitations I would also mention that this was a 10-day study in a specific period of the year, the dry season I suppose, and that these results may change markedly during the rainy season due to multiple factors including the distribution of water sites (temporary waterbodies) and changes in accessibility.

Line by line

• L: 88-92: it is unclear to me whether it is a common assumption that more mobility means more water contacts. Could the authors provide examples for these? In lines 725-726 the authors mention modeling studies, but I don't think that this was the assumption in those papers. If so, could the authors provide explicit where this assumption is made in these papers?
• Figure 1: I think that location intensity maps for all three districts would make a nice figure to show the raw data behind the analysis. As usage duration is an important outcome, could a histogram of these also be provided?
• L 217: Was this correlation true across age categories/occupations?
• Table S1: please also show the IQR for tap distances
• Fig S2: overlay with self-reported
• Figure 3: It would be clearer to interpret these with the corresponding model equations
• Terminology: in practice these are all logistic regression models, some log-linear and some non-linear in distance.
• Table S2 is logistic regression, is it a coincidence that it is the same auROC than reported for spatial decay model in line 326.
• L. 337-339: Is there a reason why these covariates could not interact? I also wondered whether the authors considered investigating the difference in the effects by district. One could also imagine a hierarchical model for the effects of covariates by district.
• Figure 4: In the dots in panel a please add the number of observations at each distance and possibly binomial Cis. The validation of observed v.s predicted durations could be done with a scatterplot as well, as it is hard to see here.
• L. 400: It is unclear to me how to compare these parameter values as the output units are different (probability of usage vs. number of minutes). and that the decay functions f are not bounded to 1 (but I understand the product of f and b0 is).
• Fig 5: Was there any variation of these results when considering each district separately?
• L. 473: how was the p value computed here?
• L. 473-475: please account for the uncertainty, that goes close to 0, in this statement
• Fig 6a-b: the correlations are the same in the crowding and non-crowding models, could the authors comment on this?
• L. 512: If feasible it would be interesting to have results on the other districts as well.
• Fig 8: what are the units of performance?
• Table S4: more likely to have tap water at home in missing data. Could the authors comment on this?
• Table S5: unclear if number of points also depends on displacement or just timing.
• L. 705-707: I believe that these are not comparable things (probability of movement to a place vs. fluxes), or maybe I am missing something?
• L. 709: It is unclear to me what water contact means here. Is it models
• L. 713: the covariate analysis is not convincing at this point
• L. 722: what about tracks in the water for sifhermen?
• L. 738: Is this for schistosomiasis? I believe that the references from Whoolhouse et al., Ciddio et al, and Mari et al., cited by the authors, are spatial models. I would also mention Mari et al. 2017.
• L. 741: all this paragraph is subject to the validity of the simulations model, which in my opinion is not fully supported by the model comparison results at this stage
• L. 769: this is not done here
• L. 803: this is in my opinion a major limitation, and should be expanded on (see Major points)
• L. 960: It is unclear to me whether a water usage is therefore being in proximity to the median point? It would be very useful to visualize these.
• L. 1180: Using a cutoff does not correspond to the data generating process implied by equations 2-5, and it is therefore unclear to me if this is a proper way to check model validity. Could the authors expand on this?
• L. 1194: unclear how this works
• L. 1206: It is unclear to me if this is in fact more difficult without a mathematical argument. May be worth removing.
• Fig S10: why is it 451 here and 452 in main text?
• In the code there seems to be a zero inflated neg binomial to model usage durations, but this is not mentioned in the main text, or maybe I am missing something?

References

Paz-Soldan, V.A., Reiner Jr, R.C., Morrison, A.C., Stoddard, S.T., Kitron, U., Scott, T.W., Elder, J.P., Halsey, E.S., Kochel, T.J., Astete, H. and Vazquez-Prokopec, G.M., 2014. Strengths and weaknesses of Global Positioning System (GPS) data-loggers and semi-structured interviews for capturing fine-scale human mobility: findings from Iquitos, Peru. PLoS neglected tropical diseases, 8(6), p.e2888. https://journals.plos.org/plosntds/article?id=10.1371/journal.pntd.0002888

Other study on water usage: Crow, B., Davies, J., Paterson, S. and Miles, J., 2013. Using GPS and recall to understand water collection in Kenyan informal settlements. Water international, 38(1), pp.43-60. 10.1080/02508060.2013.752315

Mari, L., Ciddio, M., Casagrandi, R., Perez-Saez, J., Bertuzzo, E., Rinaldo, A., Sokolow, S.H., De Leo, G.A. and Gatto, M., 2017. Heterogeneity in schistosomiasis transmission dynamics. Journal of theoretical biology, 432, pp.87-99.

(Remarks on code availability)

I was not able to review all the code, and some scripts seemed to be missing to reproduce the analysis.

Reviewer #2

(Remarks to the Author)
The investigators for this study collected an extensive dataset on tap/borehole usage and water contact activities among residents of 3 villages in Uganda. They developed a wide-ranging set of models assessing how different variables (mobility, age, sex, distance from water source) affected usage. Unfortunately, although the models developed by the team were highly sophisticated, there were no real biological questions with respect to effects on schistosome infection clearly hypothesized or answered. There were some model comparisons on open water usage related to household location in relation to water contact location and proximity of safe water (Fig 3) but no overarching study question. The authors did note (without showing the data) that increased open water usage was associated with increased infection rates after one year but show no data on whether the people who had a greater proportion of tap/borehole usage has any reduction in infection prevalence or intensity. Perhaps this is because even the most frequent users of improved water sources only reduced their open water contact by 23 and duration by 33%. Similarly, it would be helpful to know the +1 year infection status of the ~35% of people who never visited a water site during the 10 days of data collection. The authors evaluated the first day of recording for Hawthorne effects but if the individuals with no water contact are infected at similar levels as those who had open water contact during the 10-day tracking, it would argue that participants may have behaved differently while being tracked that they did the rest of the year.

It may be that there was no impact of tap/borehole access/use on schistosome infection. While this would certainly be a disappointing result for the investigators, it should still be clearly reported as it would likely temper expectations that programs focused on provision of water access will influence risk of schistosome infection. That does not mean they should not be installed; it just means that other interventions for schistosomiasis could be more effective. The authors do address this in passing ("Our results suggest caution in assuming that the provision of safe water would reduce schistosome transmission") but the message somewhat gets lost among the focus on the models over the biology. The non-effect (presumably) of the increased tap/borehole usage on infection levels should be noted in the abstract.

One curiosity is that bathing is not listed among the 11 water contact activities that the investigators collected. That activity may be the one most associated with infection risk (but perhaps also when participants were most likely to remove their tracker). For those who predominantly obtained their water from taps and boreholes, is that enough water for bathing purposes? It would be useful to understand bathing behaviors among the study group and how they may have affected the results.

The buffer size of the trackers was 20-30 meters. For people who have a tap in their home or compound, it seems like they would be in a perpetual contact event. How did the investigators address this?

How did the time 0 infection levels of study participants compare with their infection prevalence and intensity at one year? Were pre-treatment infection levels a greater or lesser predictor infection after one year that relative tap/borehole usage?

Did the investigators ask the study participants about the effects of cost on water usage? Even though the costs per jerry can or monthly borehole access were minimal by western standards, they are still a cost. How much did the fact that open water access is free affect the study outcomes? If cost as a factor, even the most optimal placement of improved water access may be inconsequential for effect on schistosomiasis transmission.

Minor corrections:
--WASH stands for water, sanitation, and hygiene, not just water and sanitation.
--In figure 1d, the color designations for tap/borehole vs open water site in the inset contradict the colors as defined for 1a.

(Remarks on code availability)
I do not have sufficient expertise to comment on the code.

Reviewer #3

(Remarks to the Author)
Thank you for sharing your work carried out in Uganda. Although an interesting study, the structure of the manuscript seems to differ from standard publications. In addition, involvement of more researchers from Uganda would have been ideal. Below are further comments for consideration.
- Shouldn't schistosomiasis appear in the title of the paper?
- Does 'safe and unsafe' need to appear in the title of the paper?
- In the spirit of equitable partnerships, it would have been ideal to have more co-authors from Uganda.
- Why was the age group of 5 to 82 years considered for the study?
- Abstract: it is generally discouraged to start sentences with figures (such as '64.4% .....).
- The aim of the study should be provided explicitly in the abstract before the methods.
- The rationale for 'Schistosomiasis' among other waterborne vectors / diseases is not clear in the abstract.
- Simulations provided in the results of the abstract are not adequately introduced in the methods.
- The conclusion in the abstract does not seem justified based on the findings therein.
- Line 77: WASH should be an acronym of water, sanitation and hygiene.

- The introduction needs to be strengthened by providing more background information regarding access to water, WASH, Schistosomiasis, etc. in the Ugandan local context.
- More details on the current existing evidence on the subject in Uganda and the gap that was targeted by the study is needed in the introduction. This is needed before introducing the community-based study.
- More details in the methods are needed including data collection tools, who collected data, quality control, justification of the study area, etc.
- More details on the community-based study are also needed.
- How were the water sources selected?
- The aim of the study should be provided in the introduction before the methods.
- It is not clear why methods are presented in the results (for example lines 171 to 175).
- It is also not clear why there is discussion during presentation of results (for example lines 221 / 223).
- The manuscript provides vast results, hence appear too many for a single paper. The authors may consider including only the most crucial findings to address their primary objective in this paper.
- A separate paragraph with study limitations (and strengths) before the conclusion is needed.
- Why are the methods appearing after the conclusion?
- Uganda National Council for (not of) Science and Technology

(Remarks on code availability)
Not applicable as the code was not provided.

Version 1:

Reviewer comments:

Reviewer #2

(Remarks to the Author)
The authors have adequately addressed the overall concerns of the reviewers from the first version of the manuscript. In terms of the application of the findings to control programs, while it may be possible to track a small number of people in a given village and predict which water sites may be most likely to contribute to transmission, this will have to be repeated for each village and the eventual number of sites at which intervention will be needed may again be extensive.

In future studies, it would be interesting to determine which water contact activities are adequately addressed by boreholes and which are more likely to take place at open water sites. Not only volume of water needed may affect this distinction but also a desire for privacy for certain activities (e.g., bathing) that may not be possible at public taps and boreholes. The studies from Saint Lucia show that some water contact activities (e.g., washing) are communal in nature, perhaps in part answering the observation in this study that borehole/tap access does not necessarily result in reduced open water usage. But the reassuring news is that Saint Lucia is on the verge of interrupting transmission once adequate water access has become available.

(Remarks on code availability)
my background is not sufficient to address the quality of the code. However, it appears that reviewer 1 is able to do this.

Reviewer #3

(Remarks to the Author)
I am satisfied with the revisions made based on my comments.

(Remarks on code availability)

Reviewer #4

(Remarks to the Author)
I reviewed the paper to assess revisions in response to Reviewer 1’s comments. This paper fills an important gap in the schistosomiasis modeling literature: how fine-scale data on human movement can enhance modeling of water contact, and in turn, infections. While the paper has been refocused on schistosomiasis, which is appropriate, the overall approach could be useful to a wide range of other environmentally-mediated infectious diseases given that many transmission models for such diseases do not formally incorporate spatial information about exposures and contact patterns. Overall, the authors have thoroughly addressed Reviewer 1’s comments. I have a few minor comments.

The authors have clarified the choice of water contact model used in the IBM and clearly described model comparisons with consistent metrics based on ELPD, a form of Bayesian leave-one-out assessment of predictive fit. The revised sections on reinfection from the IBM are clear.

The newly added validation of water usage does indeed strengthen the paper; however, the low correlation between GPS-based open water contact and survey-based could be more explicitly mentioned in the limitations section of the Discussion.

Related to comment 11, in Figure S6a, the caption is not clear to me. It states that the predictions were at the individual level by individual-site pair, but the plot shows two points. Are these over-plotted, or are these summaries of the predictions?

The points on generalisability seem over-stated to me. The authors found that logger data from 15 individuals over 10 days would have been sufficient, but later they state that the 10-day period might not be representative of water usage patterns or generalisable to other seasons. I think these points should be presented alongside each other in the Discussion. Estimates of the minimum required people and days for GPS data collection were based on predictive performance metrics derived from a potentially non-representative dataset. They might not be appropriate for guiding the sample size of future studies, particularly given the data collection within one season and relatively small geographic area included in this study. Related to this, the paper refers to “transportability” as well as “generalisability”. Given that the analysis holding out one district at a time includes the same three districts used in the study, the term generalisability is more appropriate. “Transportability” would be appropriate if the results were extended to other populations not included in the original study.

(Remarks on code availability)
n/a

Open Access This Peer Review File is licensed under a Creative Commons Attribution 4.0 International License, which permits use, sharing, adaptation, distribution and reproduction in any medium or format, as long as you give appropriate credit to the original author(s) and the source, provide a link to the Creative Commons license, and indicate if changes were made.
In cases where reviewers are anonymous, credit should be given to 'Anonymous Referee' and the source.
The images or other third party material in this Peer Review File are included in the article’s Creative Commons license, unless indicated otherwise in a credit line to the material. If material is not included in the article’s Creative Commons license and your intended use is not permitted by statutory regulation or exceeds the permitted use, you will need to obtain permission directly from the copyright holder.
To view a copy of this license, visit https://creativecommons.org/licenses/by/4.0/

Reviewer Comments and Responses

Reviewer #1

Comment 1: Framing

From my take, there seems to be a mismatch between the framing of the work between the title, the introduction, the results and the discussion. The title and results sections put emphasis on water usage patterns per se, in relation to existing knowledge of human mobility patterns in general. In addition, unsafe water usage is important for many diseases, as noted by the authors. This is reflected in the stated aims of the paper in L. 123-127 which do not mention a specific disease application. Almost all the results section focuses on water usage patterns, with a very minor section on epidemiologic outcomes in L. 530-537. On the other hand, the introduction and discussion put important emphasis on the specific aspects of water contacts and schistosomiasis, relating the outcomes of the study to schistosomiasis modeling studies, and less to the general aspects of human mobility of water usage vs. other types of mobility. I understand the link between the two, as rightly mentioned by the authors in the introduction, I however believe that the very limited emphasis on epidemiologically-relevant outcomes in the results strongly limits the interpretation of the results for public health policy. A specific point on the reinfection data is given below. Linking the water usage results to schistosomiasis in the study area may be difficult because water contacts are not necessarily exposures if there are no (shedding) snail intermediate hosts. I would therefore suggest the authors consider re-aligning the framing of the study on whether it is schistosomiasis-specific or not, possibly re-dimensioning the focus given on the disease in the different sections.

Response: Thank you for these comments that have resulted in a substantial rewrite of our paper and addition of new experiments to better align the results with the public health impact. You mentioned that there seemed to be a mismatch between the framing of the work in the title, the introduction, the results and the discussion. To address this mismatch, we have focused the paper entirely on schistosomiasis, and we make this focus clear in the title and abstract now as well. We also completely overhauled the introduction to be schistosomiasis-specific.

Your second major point relates to the very limited emphasis on epidemiologically relevant outcomes. We have made two major changes to address this point. First, we now report more extensively on infection outcomes and have added a new results section entitled ‘Relationship of water usage with schistosome (re)infection’, which appears early on in the results to highlight how GPS-derived water contact measures are related to (re)infection outcomes. We also provide more extensive descriptive statistics and comparisons between GPS-derived and self-reported water contact measures and their associations with infection outcomes in newly added supplementary materials. The second major change, and most substantial change that required additional data and experiments, as well as the addition

of a new co-author with expertise in mathematical modelling, was to build a spatially explicit individual-based schistosome transmission model that is informed by the model-based estimates of water contact derived from the GPS data. Critically, we found that the use of spatial-decay informed assumptions in schistosome transmission models not only enables a spatially explicit focal approach (important for targeting of interventions) but also outperforms even conventional empirical assumptions of how individuals choose which open water sites to use and how this affects reinfection. Models with spatial decay (as learned from our wearable GPS loggers) are able to best predict reinfection prevalence within our endemic communities. With these changes, we believe that our paper now has a significantly stronger emphasis on epidemiologically relevant outcomes and therefore a much-enhanced public health relevance.

Comment 2: Model formulations

In equation 2, it is unclear to me what the modelled outcome is. Is it whether there was any occurrence of water usage of a specific place by individual I throughout the study period? This is confusing to me in L. 1206-1210 which seem to indicate that each individual can only choose one water site at each time, although there may be multiple usages during the study period. From what I understood the number of days of participation is not the same for all participants. Is this accounted for in the data likelihood? Could the authors comment on why a discrete distribution is used to model average daily contact duration during the study period?

Response: Thank you for this comment. In this paper, the modelled outcome is any occurrence of water contact at site j for individual i during the study period, with no constraint on the number of sites individuals can visit, as individuals can and do visit multiple sites (as shown in the descriptive data). We appreciate that the wording in lines 1206-1210 could have been confusing, and we have reworded those lines to make it clear that our models do not constrain individuals to just visit one open water site.

You are also correct to note that the number of days of participation is not the same across all participants. We investigated the influence of the number of observation days on our spatial decay results in **Fig. S12b**. Among study participants, who had a minimum of two days of data though the additional number of days varied, we showed that only a minimum of one observation day from all participants was needed to train the spatial decay model of open water contact (**Fig. S12b**), illustrating that our models were robust even when trained with substantially less data than we had available. Given the low number of observation days needed, we did not specifically adjust for this in the likelihood, especially as our aim is to use the GPS data as a representation of individuals' typical water contact patterns, not just patterns within a set number of days.

We chose a discrete model because the duration data showed strong overdispersion and many zeros, which continuous distributions handle poorly. After examining the empirical distribution, we found that a zero-inflated negative binomial (ZINB) provided a markedly

better fit than a Gamma family model: the Gamma struggles with the heavy right tail in our data and is not appropriate for data that have zeros in the response, as per the 'brms' package documentation. Although a hurdle-Gamma model could technically handle zeros, it assumes that zero and non-zero durations are generated by entirely separate processes, which does not match how these contacts arise. To use the ZINB, we discretised durations by rounding to the nearest minute. This affected fewer than 1% of observations (mostly very short contacts), so the information loss is minimal, especially given that minutes are already a fine time scale for these data. Overall, we believe the ZINB formulation captures both the overdispersion and the excess zeros in a way that aligns with the underlying behaviour of the contacts.

Comment 3: Simulations

The validity of the simulation study depends on how well the mechanistic effects producing water usage shifts area captured by the model. In its current state, the data does not seem to support the crowding-out model in a clear way (see point above), which makes me doubt on the reliability of its use for simulations. I would assume that some types of water usages may be more avoidable than others in the presence of a tap/borehole, and that this would be very valuable to account for in this analysis, possibly through the use of covariates. Another point is that it does not seem to me that the presence of tap water in the household itself was accounted for here, which may be an important effect modifier of the the addition of new tap/boreholes. I would ask the authors to comment on these points as to wether they believe their simulations reliable capture the variability in effect of adding tap/boreholes in shifting water usage patterns.

Response: We fully agree with you that any simulation results hinge on the ability of the crowding out model to adequately capture the water contact dynamics in a way that outperforms simple models based on spatial decay only. As you helpfully point out, given that the crowding model did not outperform the simpler spatial decay model, we have removed all further analyses that are based on the crowding out model from the main text. To investigate the question you raised here, i.e., whether some types of activities are more avoidable than others, we have implemented a new variant of the crowding out model where we estimate the crowding out coefficient a separately for individuals who self-reported engaging in different open water contact activities (grouped into recreational, occupational and domestic (as per activity grouping in our previous paper Reitzug et al. 2024) which is reported in the **Supplementary Text S1.6**. This model investigates the hypothesis that some activities, especially domestic open water contact activities such as fetching drinking water, may be more amenable to crowding out with safe water access than occupational activities such as fishing. However, we found that activity-specific crowding out coefficients were 35-39% with 95% CIs that overlapped with the overall crowding out coefficient of 33%. We thus found no evidence that specific activities were more amenable than overall open water contact. We have also adjusted the language in the methods and results sections to make it clear that no household in the GPS logger study had piped water at the household location, i.e. all households had to visit taps/boreholes or open water sites.

Comment 4: Model comparison

The core of the results builds on the comparison of different models predicting water usage and duration. Throughout the paper the authors base these comparisons on a testing set of 15% of the data, except for the choice of decay function which is based on ELPD. Both of these metrics aim at assessing predictive performance. I would therefore suggest the authors choose one and use the same throughout the paper. If would personally suggest ELPD, having a testing dataset is also fine. I would however not base all testing on a single holdout, but instead do a K-fold cross-validation scheme with proper accounting of the relative number of participants per district. I would also urge the authors to report uncertainty intervals and all metrics (ELPD, auROC, sensitivity, specificity), and detail in the methods the methodology to discriminate between models. There are in fact multiple instances, where the terminology is unclear to me (for instance in saying that an auROC of 0.890 is larger than 0.888 without reporting uncertainty intervals and a definition of what a significant difference is, which to me in this case is effectively equivalent and would not choose a specific model based on it). I believe this is particularly important for the crowding-out model on which the simulations are based, as if the data does not support it better than the simple spatial decay model I believe it undermines its use in the simulations section. I also wondered why the different decay functions were not compared in the other model version (Fig3. B-d), as these different model formulations may turn out to support other functions.

Response: We agree that uncertainty estimates were needed. We have followed your suggestion and now report ELPD as our primary metric for model selection. For each model pair we computed the difference in ELPD and its standard error using leave-one-out cross-validation (LOOCV), and models were regarded as clearly different when $| \text{D ELPD} | > 1.96 * \text{SE(D ELPD)}$, corresponding to a two-sided p-value < 0.05, to address your concern that we were not able to distinguish whether one model performed significantly better than another model. We additionally report auROCs to provide more conventional, interpretable metrics of classification accuracy. For these metrics, we use stratified 5-fold cross-validation and 95% CIs using the recommended DeLong method, where stratification is done for districts for proper accounting of the relative number of participants per district, as suggested. We also agree with your assessment that the ELPD model comparison does not support the crowding out model being better than the simple spatial decay model and we have removed all simulations based on the crowding out model that were part of our original submission. We have also de-emphasised all other parts of the crowding out analyses and moved them into supplements (but kept them in the supplement just to make the point that even if these counterfactual scenarios are assumed to be true, the impact of safe water provision on open water contact remains limited).

Regarding your comment on using different decay functions in the other model version (**Fig 3. b-d** in the previous version): we see models b-d as incremental variations on the basic simple decay model. We followed a sequential model-building process whereby we first identified the best-fitting decay function and then built on this finding when incorporating additional complexity into the models. We are more explicit about the sequential nature of

the model-building process in the updated version of the Methods section entitled ‘Spatial decay models’.

Comment 5: Validation of water usage results

Although the authors use the term "water usage", it technically consists of inferences made from GPS tracks which cannot be considered as perfect (Paz-Soldan et al., 2013). The authors have published detailed work on water usage practices in the area, from what I understand from the same participants. I was therefore wondering if some aspects of the GPS-derived water usage data could be validated against questionnaire data, for instance the proportion reporting any contact during the season of the study. This would greatly strengthen the results in my opinion. Regardless, I think that more explicitly mentioning the limitations of the GPS data to infer water usage in the limitations paragraph would help the reader assess the study.

Response: Thank you for these suggestions to strengthen the results of the paper. We have now included a detailed validation section of GPS-derived open water site usage against survey-reported open water contact patterns in the **Supplementary Text S1.4**. We found that among study participants, 63.9\% (289/452) used one or more open water sites, based on the GPS data over the 10-day observation period. In the survey data, 51.1\% (231/452) among those same participants reported they had any contact with open waterbodies in the past week. The correlation between those two measures of open water contact was Spearman’s rho = 0.10 (p=0.03). We also acknowledge the limitations of GPS data for approximating water contact towards the end of the discussion, as open water contact is inferred from GPS logger positions and not directly observable, there is a risk that our estimates of open water contact have noise (though we have no reason to believe them to be systematically biased).

Comment 6: Schistosomiasis reinfection data

It was unclear to me how to interpret the results sentences on the reinfection data. This seems to be an important point, as it supports the public health relevance of the whole water usage study. Could the authors expand on these results more in terms of the aspects of water usage that relate to reinfection probability.

Response: Thank you for this comment. We completely agree with you that it is important to relate the model-based estimates of water site usage with the reinfection data, and we have substantially revised the parts of the paper that make the link between water contact and infection. In the results section entitled ‘Using model-based open water site contact probabilities to inform open water contact in IBMs’ we show both descriptively and through an individual-based model (IBM) how our model results significantly and positively correlate with and predict reinfection, respectively. We also added additional **Supplementary Text S1.1** that provides more extensive results about how GPS-derived and self-reported water contact data are related to reinfection intensity. What is most interesting is that the IBMs

informed by spatial decay information from our main model produce more realistic estimates of one-year reinfection prevalence at the community level (**Fig. 7**).

Comment 7:

Could the authors please provide a full mathematical description of the models in the analysis, a table with equations for all spatial decay models would be very helpful.

Response: Thank you very much. We have added a new **Supplementary Table S7** that provides a comprehensive overview of all models that appear in this study.

Comment 8:

It was unclear to me what the probability of tap/borehole usage corresponds to in equation 5. Is it to the closes tap for person i?

Response: We added additional information in the methods to highlight that, as in the other models, the probability of tap/borehole usage is estimated separately for each individual and open water site combination, not just the closest site.

Comment 9:

As almost all tap usages are with the closest one, I was curious to know how the spatial decay model fairs against logistic regression models.

Response: Thank you for this suggestion. Analogously to what we report in **Table S2**, we also constructed a simple logistic regression model where we assigned all individuals to using the closest tap/borehole. Based on LOOCV, this model performed significantly less well than the exponential spatial decay model of tap/boreholes usage (ELPD difference = –152.6, p-value < 0.001).

Comment 10:

From table S1 it seems like a good proportion of households had tap water in their home, which I suppose can have an important impact on water usage outside the household. Was this accounted for in the models? May this explain part of the variability in any water usage, which the models seem to consistently over-estimate (figure 6c).

Response: Thank you for this comment. 'Household drinking water source = improved' in **Table S1** refers to whether households self-reported fetching their drinking water from public taps/boreholes, as no households had taps or within-home safe water access. We have added a clarifying sentence in the methods section to highlight that there were no participants with safe water access within their household compounds. All 'improved drinking water sources' were public facilities (taps/boreholes) requiring travel from the household.

Comment 11:

I would like to see posterior retrodictive checks of the number of water contact points used by individual to compare to figure 1b.

Response: Thank you for this comment. Our understanding is that you would like to see a figure that compares the number of open water sites an individual is predicted to use based on the spatial decay model estimates to the observed number of such contacts shown in **Fig. 1b**. We have now created a Supplementary Figure (**Fig. S6a**) that shows how well the predictive distribution fits the observed data. We also provide a comparison of the predicted versus actual number of open water sites used per individual in **Fig. S6b**.

Comment 12:

The authors use 30m to define a buffer for water usage. I would encourage the authors to provide a sensitivity analysis of buffer width on the main results of the paper.

Response: Thank you very much for this comment. We provide a sensitivity analysis in **Fig. S14** where we compare buffers of 20m and 30m. Based on the GPS accuracy (8.6m) and accuracy of site GPS points (5-10m), we believe that 20m and 30m represent the reasonable upper and lower buffer bounds, respectively, for this analysis. Based on this robustness analysis, we now report all our main analyses with the more conservative buffer as this avoids false positives (i.e. transient movement in proximity to sites that does not represent a true contact event). We also note that this buffer is more conservative than any existing GPS logger studies on schistosomiasis; Eyre et al. (2020) used a 30m buffer around the shoreline while Seto et al. (2012) used a 100m buffer around the shoreline. In addition, we also remove all open water contact events within 20m from the household location to avoid misclassifying activities taking place at home as water contact.

Comment 13:

In the limitations I would also mention that this was a 10-day study in a specific period of the year, the dry season I suppose, and that these results may change markedly during the rainy season due to multiple factors including the distribution of water sites (temporary waterbodies) and changes in accessibility.

Response: We agree with your explanation and have included these characteristics of the study as limitations in the Discussion.

Comment 14:

L: 88-92: it is unclear to me whether it is a common assumption that more mobility means more water contacts. Could the authors provide examples for these? In lines 725-726 the authors mention modeling studies, but I don't think that this was the assumption in those

papers. If so, could the authors provide explicit where this assumption is made in these papers?

Response: In the papers we mentioned (Ciddio et al. 2017 and Hardwick et al. 2021) there are in-built assumptions around the relationship between human mobility and water contact. For instance, Hardwick et al. write "we have developed and studied spatial models of human movement to infectious focal points" (p. 11) with mobility data approximated using building footprints. Hence, their model posits a direct relationship between mobility and contact with water sites. Ciddio et al. also posit a direct relationship between mobility and water contact. They write "The first layer of spatial connectivity is defined by human-to-water contact patterns, which link the two sets of nodes of our network through the $nv \times nw$ contact matrix, $W= [W_{vw}]$. In the absence of comprehensive surveys on water use in the study area, contacts between human villages and water points have been estimated from proxies of human mobility" (p. 410). Thus, the matrix W is derived from mobility data and posits an equivalence between mobility and water contact.

Reviewer #2

Comment 1:

The investigators for this study collected an extensive dataset on tap/borehole usage and water contact activities among residents of 3 villages in Uganda. They developed a wide-ranging set of models assessing how different variables (mobility, age, sex, distance from water source) affected usage. Unfortunately, although the models developed by the team were highly sophisticated, there were no real biological questions with respect to effects on schistosome infection clearly hypothesized or answered. There were some model comparisons on open water usage related to household location in relation to water contact location and proximity of safe water (Fig 3) but no overarching study question.

Response: We have substantively revised the paper to focus on the public health impact, and have developed an individual-based transmission model to test the informativeness of including spatial decays learned from wearable GPS logger data. As we now clarify at the end of the introduction, "The main aim of this study was to model open water contact and tap/borehole usage with fine-scale spatial data to develop assumptions around which individuals have contact with which open water sites. The assumptions were validated by developing spatially explicit IBMs of schistosome reinfection. A secondary aim was to evaluate whether models that incorporate WASH usage outperform models with only spatial information for predicting human water contact at open water sites."

By incorporating a transmission model that draws upon our model-based estimates of open water site usage, we now demonstrate the relevance of water site usage information for predicting one-year schistosome reinfection. We do this by showing how our model results improve upon simpler, conventional approaches for integrating water contact into transmission models (**Fig. 7**), and also provide the first spatially-explicit model to inform focal interventions for schistosomiasis control.

Comment 2:

The authors did note (without showing the data) that increased open water usage was associated with increased infection rates after one year but show no data on whether the people who had a greater proportion of tap/borehole usage has any reduction in infection prevalence or intensity. Perhaps this is because even the most frequent users of improved water sources only reduced their open water contact by 23 and duration by 33%. Similarly, it would be helpful to know the +1 year infection status of the ~35% of people who never visited a water site during the 10 days of data collection.

Response: Thank you for this comment. You are correct to point out that while we reported on the association between open water usage and reinfection in the results subsection, we did not show any data on how tap/borehole usage was associated with

reinfection outcomes. To address this, we have now added additional results in the results section entitled 'Association of open water contact with schistosome infection outcomes', where we report correlations between reinfection intensities and the duration of tap/borehole usage. We also provide a comparison of reinfection intensity and prevalence between individuals with and without any open water contact (using both GPS-derived and self-reported measures of water contact) in **Supplementary Text S1.1**. Given that we focus on a 10-day observation window, even if GPS loggers perfectly captured water contacts within the study period, due to variation in these patterns and in environmental risk factors, they may not be expected to perfectly correlate with infection outcomes. Specifically, we found that among people who were classified as having no open water contact based on GPS data and were not found to be infected after treatment, 24.6% (35/142) became reinfected after one year. Similarly, among people who were classified as having no open water contact based on self-reports, 37.0% (67/181) became reinfected after one year. Given that people can remain infected even years after they have ceased to be exposed or that one exposure event may be sufficient for someone to become infected, it is not entirely surprising that there are infected individuals who are not having any current water contact. With these new results, we have attempted to provide a fuller picture of how water contact and tap/borehole usage were associated with reinfection in this study. Our transmission modelling analysis also shows that a model with more realistic incorporation of water contact based on the GPS data showed better ability to predict reinfection compared to simple assumptions, such as assigning individuals always using the closest site (**Figs. 8-9**).

Comment 3:

The authors evaluated the first day of recording for Hawthorne effects but if the individuals with no water contact are infected at similar levels as those who had open water contact during the 10-day tracking, it would argue that participants may have behaved differently while being tracked that they did the rest of the year.

Response: We have used several strategies to minimise the impact of Hawthorne effects on our results. We excluded the first day of observation to enable participants to get used to wearing the logger before we collected data. We compare overall wear-compliance between different demographic groups, showing that there were no clear non-compliance patterns based on self-reported water contact patterns or occupation. Your comment suggests that if individuals with and without water contact are infected at similar levels, it follows that participants must have behaved differently while being tracked than they did the rest of the year. While this remains possible, we have now included more extensive comparisons between self-reported and GPS-derived water contact data, which we report in the **Supplementary Text S1.4**. We also note that measures of water contact are not necessarily strongly correlated with infection outcomes. In our previous paper from the SchistoTrack study (Reitzug et al. 2024), we demonstrated that self-reported typical water contact (which we validated with direct water contact observation data, arguably a gold-standard exposure measure) was only very weakly correlated with infection status. Therefore, a weak correlation between water contact and infection does not necessarily

mean that the exposure measure is unreliable, as water contact is only one element of schistosome exposure. And, given the length of schistosome infections, individuals without current water contact can still be infected. Of course, we cannot conclude with certainty that our 10-day observation period is representative of general water contact patterns, and we have highlighted this as a limitation, including a potential limitation of using our models in the rainy season, but we chose this dry season period because this is the time when we know that water contact is highest. Therefore, even if contact patterns change seasonally, we have almost certainly captured the most relevant timepoint for schistosome transmission.

Comment 4:

It may be that there was no impact of tap/borehole access/use on schistosome infection. While this would certainly be a disappointing result for the investigators, it should still be clearly reported as it would likely temper expectations that programs focused on provision of water access will influence risk of schistosome infection. That does not mean they should not be installed; it just means that other interventions for schistosomiasis could be more effective. The authors do address this in passing ("Our results suggest caution in assuming that the provision of safe water would reduce schistosome transmission") but the message somewhat gets lost among the focus on the models over the biology. The non-effect (presumably) of the increased tap/borehole usage on infection levels should be noted in the abstract.

Response: Thank you for this comment. We have incorporated your suggestion and state in the abstract that there was no evidence for an impact of tap/borehole usage on open water site usage, as evidenced from the crowding out model not outperforming a simple spatial decay model (suggesting that crowding out does not influence the dynamics determining the probability of open water contact). We also revise the discussion to indicate that although there is no evidence of WASH diverting open water contact, more research is needed to determine whether individual activities are important to divert and whether WASH is essential for public health regardless of its relationship with open water contact.

Comment 5:

One curiosity is that bathing is not listed among the 11 water contact activities that the investigators collected. That activity may be the one most associated with infection risk (but perhaps also when participants were most likely to remove their tracker). For those who predominantly obtained their water from taps and boreholes, is that enough water for bathing purposes? It would be useful to understand bathing behaviours among the study group and how they may have affected the results.

Response: Thank you for this comment. Bathing was indeed one of the 11 water contact activities we collected and analysed, and we have changed the text to highlight this. You are right that participants may be naturally inclined to remove GPS loggers during bathing activities. As part of our community sensitisation activities, we therefore explicitly

encouraged participants to keep wearing loggers during such activities or at least leave the loggers at the water site (because this enables swimming without a logger to be classified as a water contact event). As the GPS data does not provide information on the exact activities individuals engage in, it remains challenging to further explore this using the GPS data, and we acknowledge this as a limitation. However, we already reported a detailed analysis of bathing behaviours in SchistoTrack in our previous publication from the same study (Reitzug et al. 2024). Our previous detailed descriptive analysis using the self-reported information on bathing from the SchistoTrack study (reported in Tables S3-7 in Reitzug et al. 2024) provides comprehensive information on bathing behaviour within the study. The self-reported data suggest that bathing is not a particularly common activity. In terms of water usage from taps/boreholes, this is not necessarily related to bathing behaviour or usage of open water sites for other purposes. We would expect people to still engage in bathing even when they use public taps/boreholes. In SchistoTrack, among people who used taps/boreholes, 6.7% (10/150) reported bathing with/without soap and among people not using taps/boreholes, 5.0% 15/302) reported bathing with/without soap (p=0.46).

Comment 6:

The buffer size of the trackers was 20-30 meters. For people who have a tap in their home or compound, it seems like they would be in a perpetual contact event. How did the investigators address this?

Response: No household in our study had access to safe water sources within their home or compound. All taps/boreholes were public facilities located outside residential areas. We clarify this context in the main text now. To ensure robustness of our classification of water contact events, we have now removed all contact events that occurred within ≤20m of the household (n=6, 0.1% of all events) to avoid a misclassification of being at home as a water contact event. This spatial separation ensures that proximity to taps/boreholes represents deliberate water collection activities rather than incidental household proximity to sites.

Comment 7:

How did the time 0 infection levels of study participants compare with their infection prevalence and intensity at one year? Were pre-treatment infection levels a greater or lesser predictor infection after one year that relative tap/borehole usage?

Response: Thank you for this comment. We have added descriptive statistics around baseline infection and reinfection levels among GPS logger study participants in the Supplementary Text to answer your first question. We have also added a Sankey diagram in the Supplement (**Fig. S2**) to show how participants' infection status, classified based on WHO Kato-Katz infection intensity categories (light, moderate, heavy infection), changed between the baseline and one-year follow-up. Performing a simple logistic regression with baseline infection intensity and GPS-derived tap/borehole usage to predict 1-year

reinfection, baseline infection intensity shows a small but statistically significant increase in the likelihood of reinfection, while tap/borehole usage has no meaningful effect.

Comment 8:

Did the investigators ask the study participants about the effects of cost on water usage? Even though the costs per jerry can or monthly borehole access were minimal by western standards, they are still a cost. How much did the fact that open water access is free affect the study outcomes? If cost as a factor, even the most optimal placement of improved water access may be inconsequential for effect on schistosomiasis transmission.

Response: Thank you for this comment. You are right that cost is an important factor that may influence observed tap/borehole usage patterns. In SchistoTrack, cost data was collected during the comprehensive infrastructure mapping of all taps/boreholes. We report those costs in the section 'Patterns of open water contact and tap/borehole usage' within the results. However, we note that there was no variation in those costs between villages and districts and that variation was based on the kind of infrastructure; boreholes were paid for with a monthly fee, while taps were paid for per jerry can filled. Among the models shown in **Table S3**, we present one model that predicts the distance decay separately for taps and boreholes. This is relevant because if the different pricing of taps and boreholes creates different propensities to use taps and boreholes, we would expect to see such a model performing well. However, as shown in **Table S3**, this model does not perform particularly well. In the discussion, we have added a short reference to costs to highlight that these are influencing factors of usage and that more research is needed to understand how costs affect usage, given there was no variation in our study.

Reviewer #3

Comment 1:

Shouldn't schistosomiasis appear in the title of the paper?

Response: Thank you for this comment. We have revised the title to explicitly reference schistosomiasis.

Comment 2:

Does 'safe and unsafe' need to appear in the title of the paper?

Response: Thank you for this comment. We have changed the title and removed reference to safe and unsafe water contact in the title.

Comment 3:

In the spirit of equitable partnerships, it would have been ideal to have more co-authors from Uganda.

Response: We acknowledge the importance of equitable research partnerships and collaborative authorship. The study PIs (GFC from Oxford and NBK from Uganda) have been working together for over 16 years in Uganda. Our selection of study authors was based on ICJME criteria. However, now that the manuscript includes new data and experiments, we have included two additional Ugandan authors who have contributed to this work.

Comment 4:

Why was the age group of 5 to 82 years considered for the study?

Response: In SchistoTrack, we included only individuals aged five years and older, as this represented the minimum age for praziquantel eligibility at the time of the study implementation, as per WHO guidelines. This sampling approach resulted in a broad age range of 5-82 years, which provides valuable insights into water contact patterns across the entire age spectrum of the affected population.

Comment 5:

Abstract: it is generally discouraged to start sentences with figures (such as '64.4% .....').

Response: Thank you. The abstract has been revised, and no sentence starts with a numeral.

Comment 6:

The aim of the study should be provided explicitly in the abstract before the methods.

Response: We have revised the introduction to clearly state the main study aim. "The main aim of this study was to model human water contact and tap/borehole usage with fine-scale spatial data to develop assumptions around which individuals have contact with which open water sites. The assumptions were validated by developing spatially explicit IBMs of schistosome reinfection. A secondary aim was to evaluate models that incorporate WASH usage outperform models with only spatial information for predicting human water contact by open water site."

Comment 7:

The rationale for 'Schistosomiasis' among other waterborne vectors / diseases is not clear in the abstract.

Response: Thank you for this comment. We have substantially changed the paper to focus only on schistosomiasis, and we have modified the abstract to address your comment. We now clarify in the abstract that we focus on schistosomiasis as it is a major waterborne disease and is characterised by fine-scale heterogeneity, which requires granular water usage data to better characterise the transmission dynamics.

Comment 8:

Simulations provided in the results of the abstract are not adequately introduced in the methods.

Response: We have removed the simulations from the paper. This was done because the crowding out model, which incorporated tap/borehole usage, did not outperform a simpler spatial decay model without crowding out. Therefore, we had no basis to claim that the simulation based on the crowding out model best represented the ground truth.

Comment 9:

The conclusion in the abstract does not seem justified based on the findings therein.

Response: We have changed the conclusion based on refocusing the paper around schistosomiasis and believe that our new conclusion is justified based on our findings.

Comment 10:

Line 77: WASH should be an acronym of water, sanitation and hygiene.

Response: We now ensure consistent use of the complete WASH definition (water, sanitation and hygiene) throughout the manuscript.

Comment 11:

The introduction needs to be strengthened by providing more background information regarding access to water, WASH, Schistosomiasis, etc. in the Ugandan local context.

Response: Thank you for this comment. We agree that more information on schistosomiasis and WASH would be helpful to contextualise this study. Therefore, we have added a new section at the beginning of the methods section to provide details on WASH, national schistosomiasis prevalence and MDA in Uganda, and the prevalence of schistosomiasis in the SchistoTrack study. As we seek to keep our introduction more general and believe that this study has relevance beyond Uganda, we believe that this information is best placed in the dedicated study context section within the Methods.

Comment 12:

More details on the current existing evidence on the subject in Uganda and the gap that was targeted by the study is needed in the introduction. This is needed before introducing the community-based study.

Response: Thank you for raising this issue. Within the 'study context' section, we have now added information on schistosomiasis in Uganda by highlighting the failure of MDA to achieve the schistosomiasis control targets laid out by the WHO and how the SchistoTrack study can contribute novel research evidence that could help to strengthen control programmes and monitoring.

Comment 13:

More details in the methods are needed including data collection tools, who collected data, quality control, justification of the study area, etc.

Response: Thank you for this comment. We have added a justification of the study setting in the 'study context' section. We also added a new section on the household survey data to clearly lay out who collected the data, which quality control measures were in place and which software and devices were used to administer the questionnaires.

Comment 14:

More details on the community-based study are also needed.

Response: Thank you. We have expanded the information about the community-based study in the 'Study setting and participant sampling' section. We are also providing an additional reference to a previous study from our group (Puthur et al. 2025;

https://bmjpublichealth.bmj.com/content/3/2/e002151) that provides additional detail on the larger SchistoTrack study in which this study was nested. Briefly, SchistoTrack is a cohort which was implemented as a fully community-based study embedded within 38 rural villages across three districts along Lake Albert, Lake Victoria and the River Nile in Uganda. Households were randomly sampled from local village or MDA registers, and all data collection, including surveys, clinical examinations and treatment, was conducted at central community locations such as schools and churches with support from village health teams, local leaders, and the Uganda Ministry of Health. SchistoTrack also coordinated closely with nearby government health facilities to ensure referrals and continuity of care for participants identified with health problems during community visits.

Comment 15:

How were the water sources selected?

Response: We have provide more details on how water sites were selected in a dedicated section entitled 'Mapping of village infrastructure, open water sites and taps/boreholes: "Trained malacologists from the Division of Vector-Borne and Neglected Tropical Diseases, Uganda Ministry of Health, mapped all open water sites used by study communities that were relevant for schistosome transmission, including sites outside village perimeters but accessed by community members with the help of local guides, such as village chairmen or community health workers. This comprehensive mapping approach ensured inclusion of all relevant water contact sites used by the study population, regardless of their proximity to residential areas, and is described in detail by Iacovidou et al. (2025)."

Comment 16:

The aim of the study should be provided in the introduction before the methods.

Response: The study aim is clearly provided in the last sentences prior to the Results section.

Comment 17:

It is not clear why methods are presented in the results (for example lines 171 to 175).

Response: Thank you for this comment. We have moved the radius of gyration formula and all details about its calculation to the Methods section and ensured that no other methods are presented within the results.

Comment 18:

It is also not clear why there is discussion during presentation of results (for example lines 221 / 223).

Response: Thank you for your response. We have removed this discussion sentence and have created a dedicated, new section about comparisons between self-reported and GPS-derived water contact in **Supplementary Text S1.4**.

Comment 19:

The manuscript provides vast results, hence appear too many for a single paper. The authors may consider including only the most crucial findings to address their primary objective in this paper.

Response: We have streamlined the paper by removing the simulations based on the crowding out models from the main paper and refocusing the paper more on schistosomiasis by adding a transmission model and reporting additional information regarding infection and reinfection within the study to make a stronger link between water contact and infection outcomes. At the same time, we have moved some of our previous results into the Supplementary Materials to keep the paper to an appropriate length.

Comment 20:

A separate paragraph with study limitations (and strengths) before the conclusion is needed.

Response: The manuscript includes a limitations paragraph before the conclusion. We have carefully reviewed and expanded this section based on your feedback to ensure comprehensive coverage of study limitations and appropriate acknowledgement of study strengths.

Comment 21:

Why are the methods appearing after the conclusion?

Response: This reflects the journal's formatting requirements or submission template. We will ensure methods are appropriately positioned according to the target journal's guidelines for the revised submission.

Comment 22:

Uganda National Council for (not of) Science and Technology

Response: Corrected.

Reviewer Comments & Responses

Reviewer #2 (Remarks to the Author):

Reviewer 1, comment 1: The authors have adequately addressed the overall concerns of the reviewers from the first version of the manuscript. In terms of the application of the findings to control programs, while it may be possible to track a small number of people in a given village and predict which water sites may be most likely to contribute to transmission, this will have to be repeated for each village and the eventual number of sites at which intervention will be needed may again be extensive.

In future studies, it would be interesting to determine which water contact activities are adequately addressed by boreholes and which are more likely to take place at open water sites. Not only volume of water needed may affect this distinction but also a desire for privacy for certain activities (e.g., bathing) that may not be possible at public taps and boreholes. The studies from Saint Lucia show that some water contact activities (e.g., washing) are communal in nature, perhaps in part answering the observation in this study that borehole/tap access does not necessarily result in reduced open water usage. But the reassuring news is that Saint Lucia is on the verge of interrupting transmission once adequate water access has become available.

Response: Thank you for your review of the updated version and your comment regarding the generalisability of the study findings. We agree with you that in terms of modelling local transmission dynamics using spatial decay models there is still a need to collect detailed malacological data to incorporate the environmental risk component (even in a hypothetical instance where the human decay model perfectly generalises to a new setting).

We also concur with your assessment that distinguishing between the specific water contact activities which can be expected to be reduced by the availability of taps/boreholes (i.e. crowded out) versus those that cannot is an important area for future research. We explored this in Supplementary Text section S1.6 where we did not find significant differences in crowding out (and crowding out duration) of open water contact via taps/boreholes based on whether individuals self-reported having domestic, recreational or occupational open water contact. This analysis assumes that we can infer the type of open water contact in the UPS data based on self-reports. We acknowledge that this method is approximate and highlight the inability to determine the precise type of activity from the UPS data as a limitation. This leaves an important need for future research to investigate the crowding out potential of open water contact with other methods, such as diaries, direct open water contact and tap/borehole contact observation and qualitative methods. We also note in the limitation section that the WASH delivery models (household versus public provision) require further research as they may be critical for the ability of safe water infrastructure to reduce open water contact. The Jordan et al. 1972 article you refer to (and which we have added as a reference in the discussion) is very interesting because the authors note, consistent with our finding, that "[i]n rural St Lucia, public taps provide for those domestic needs normally met by water collected and carried home from the nearby river; however, no reduction in prevalence or incidence of schistosomiasis could be detected when such a system was provided" (p. 58). Yet, when a piped water supply was installed for each household, the authors found a substantial reduction in open water contact, suggesting that importance of household-level safe water supplies (which were not present in our study area).

Reviewer #2 (Remarks on code availability):

My background is not sufficient to address the quality of the code. However, it appears that reviewer 1 is able to do this.

Reviewer #3 (Remarks to the Author):

Reviewer 3, comment 1: I am satisfied with the revisions made based on my comments.

Response: Thank you for your helpful comments throughout the review process.

Reviewer #4 (Remarks to the Author):

Reviewer 4, comment 1: I reviewed the paper to assess revisions in response to Reviewer 1's comments. This paper fills an important gap in the schistosomiasis modeling literature: how fine-scale data on human movement can enhance modeling of water contact, and in turn, infections. While the paper has been refocused on schistosomiasis, which is appropriate, the overall approach could be useful to a wide range of other environmentally-mediated infectious diseases given that many transmission models for such diseases do not formally incorporate spatial information about exposures and contact patterns. Overall, the authors have thoroughly addressed Reviewer 1's comments. I have a few minor comments.

Response: Thank you for these comments. We very much appreciate your assessment of the paper's implications beyond schistosomiasis. We have added a clause to the discussion section (in italics) to highlight this: "A similar spatial decay pattern has been suggested for water usage within the context of the NTD trachoma, but has not been rigorously evaluated using field data, *highlighting the need for future research on whether spatial decay models generalise to other environmentally mediated infectious diseases*."

Reviewer 4, comment 2: The authors have clarified the choice of water contact model used in the IBM and clearly described model comparisons with consistent metrics based on ELPD, a form of Bayesian leave-one-out assessment of predictive fit. The revised sections on reinfection from the IBM are clear.

Response: Thank you for this comment.

Reviewer 4, comment 3: The newly added validation of water usage does indeed strengthen the paper; however, the low correlation between UPS-based open water contact and survey-based could be more explicitly mentioned in the limitations section of the Discussion.

Response: Thank you for this comment. We have followed your suggestion and added new sentences in the limitations section stating "We validated GPS-derived open water contact using self-reported water contact and found a weak positive correlation. As the survey-based recall window and the UPS logger collection period within SchistoTrack were not perfectly aligned, future research using directly comparable measurement periods of open water contact is needed to more thoroughly evaluate the direct comparability of UPS-derived and survey-based open water contact."

Reviewer 4, comment 4: Related to comment 11, in Figure S6a, the caption is not clear to me. It states that the predictions were at the individual level by individual-site pair, but the plot shows two points. Are these over-plotted, or are these summaries of the predictions?

Response: Thank you for highlighting this issue. We have added a clarifying sentence (in italics) to state that "Predictions are at the individual level, that is, usage is predicted separately for each individual-site pair. *The heights of the bars indicate counts of the total number of individual-site pairs correctly and incorrectly classified*." We hope this provides clarity on how to interpret Figure S6a.

Reviewer 4, comment 5: The points on generalisability seem over-stated to me. The authors found that logger data from 15 individuals over 10 days would have been sufficient, but later they state that the 10-day period might not be representative of water usage patterns or generalisable to other seasons. I think these points should be presented alongside each other in the Discussion. Estimates of the minimum required people and days for UPS data collection were based on predictive performance metrics derived from a potentially non-representative dataset. They might not be appropriate for guiding the sample size of future studies, particularly given the data collection within one season and relatively small geographic area included in this study. Related to this, the paper refers to "transportability" as well as "generalisability". Given that the analysis holding out one district at a time includes the same three districts used in the study, the term generalisability is more appropriate. "Transportability" would be appropriate if the results were extended to other populations not included in the original study.

Response: Thank you very much for these comments. To address your points about generalisability across seasons from the 10-day window and potential non-representativeness of the UPS data, we have modified the sentence (in italics) in the discussion to incorporate these as limitations: “We also demonstrated the feasibility of using publicly available building footprints or WorldPop data to predict population-level open water contact, but note that generalisability of these models likely depends on local infrastructure, social structure, norms around open water usage, *the population-representativeness of the GPS data*, *and the extent to which the season of observation is reflective of open water contact patterns at that location.*” Thank you for highlighting the nuance around generalisability versus transportability. We appreciate this comment and have removed all mentions of “transportability”, as you suggested.

Reviewer #4 (Remarks on code availability):

n/a
